# Supplementary figures and images for: Advanced Prediction of Hepatic Oncogenic Transformation in HBV Patients via RNA-Seq Data Analysis and Deep Learning Techniques
Source: Int J Mol Sci. 2024 Sep 11;25(18):9827. doi: 10.3390/ijms25189827 (PMC11432201; doi:10.3390/ijms25189827)

**Rolling Average Coverage Plot by Base**

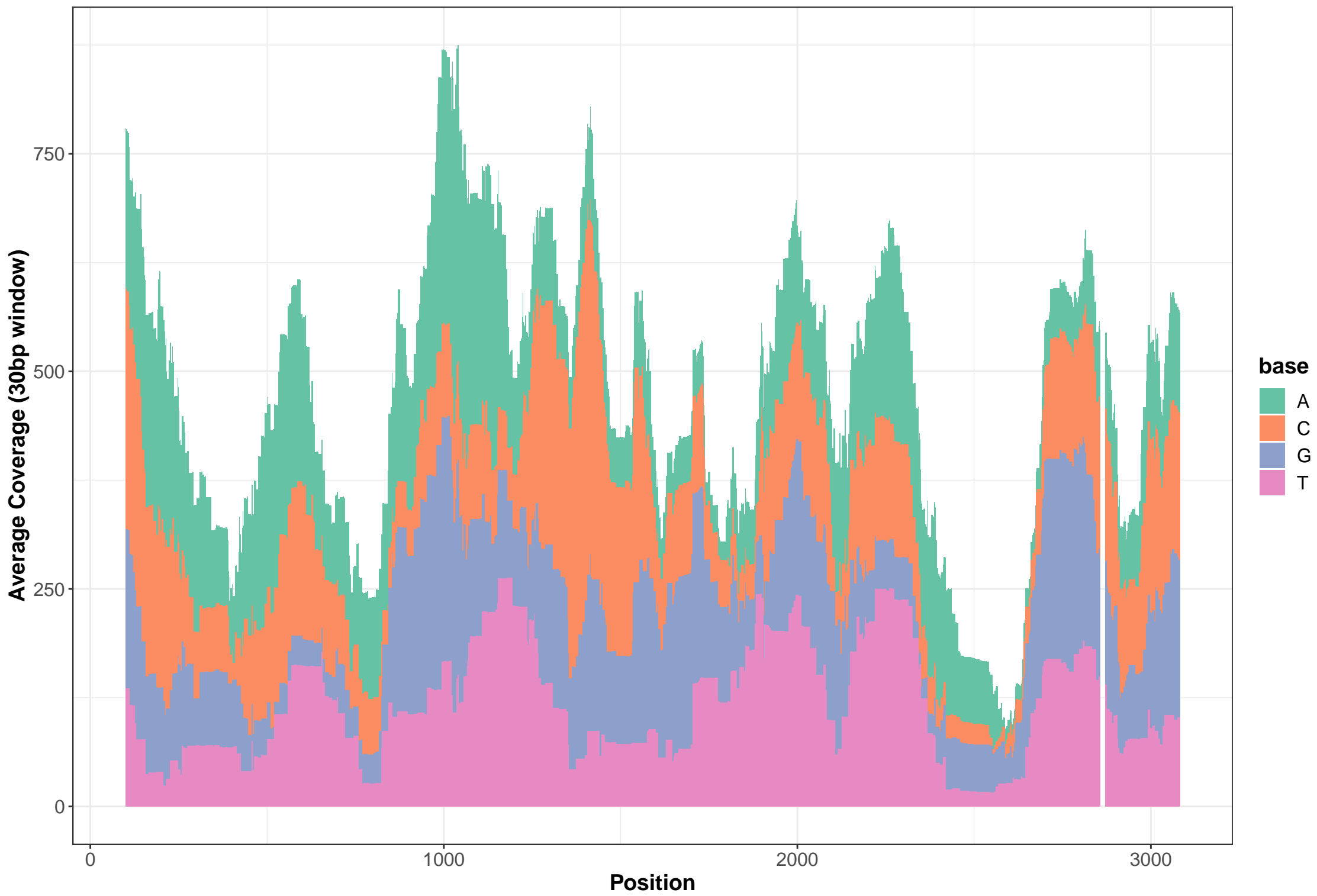

Supplement: Supplementary file 1 [file ijms-25-09827-s001.zip › Supplementary Materials S1.pdf]

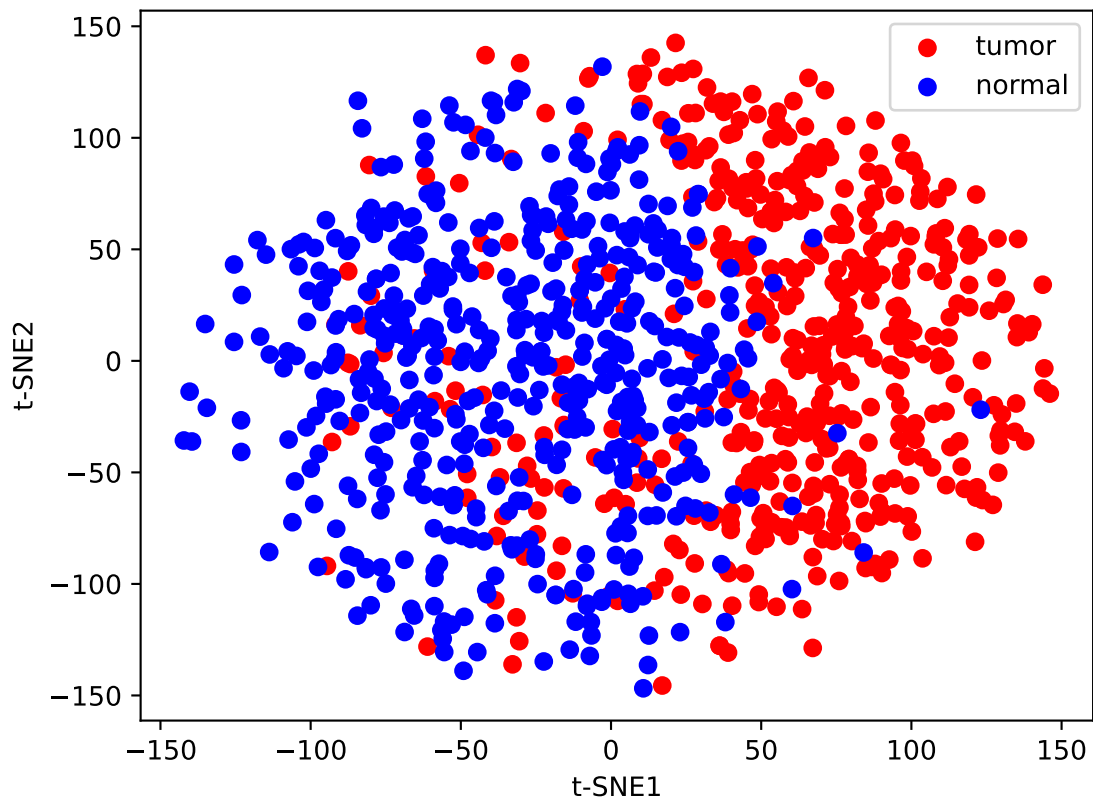

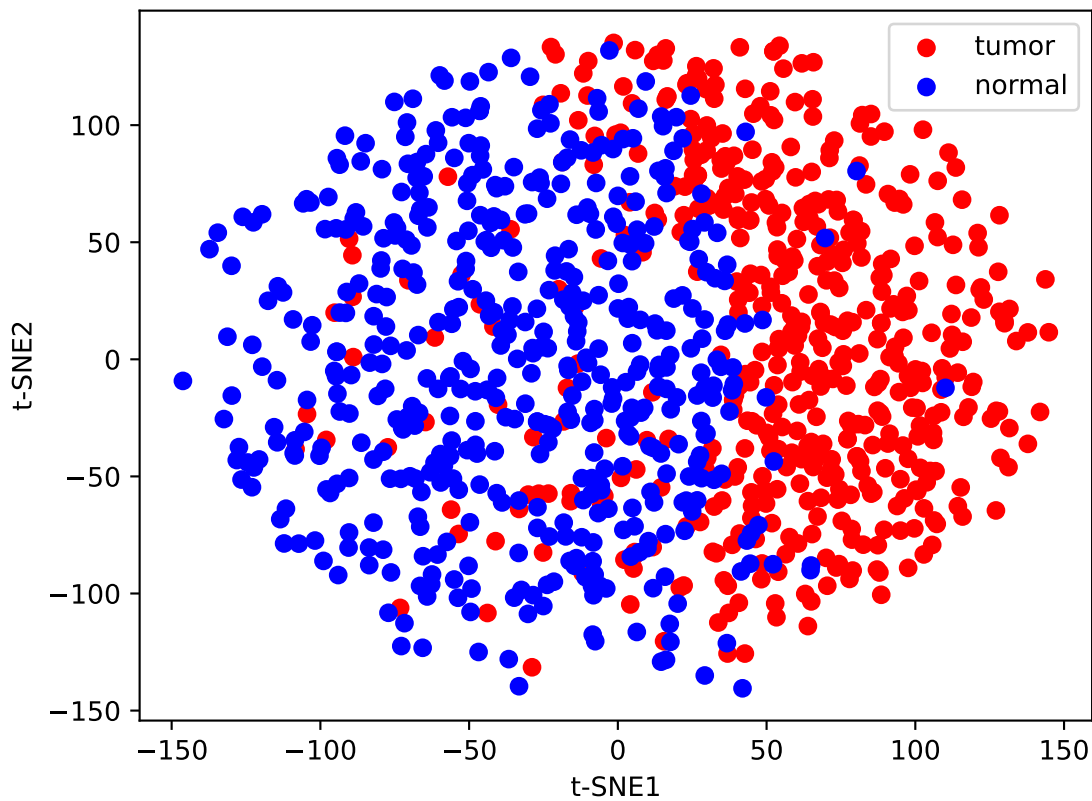

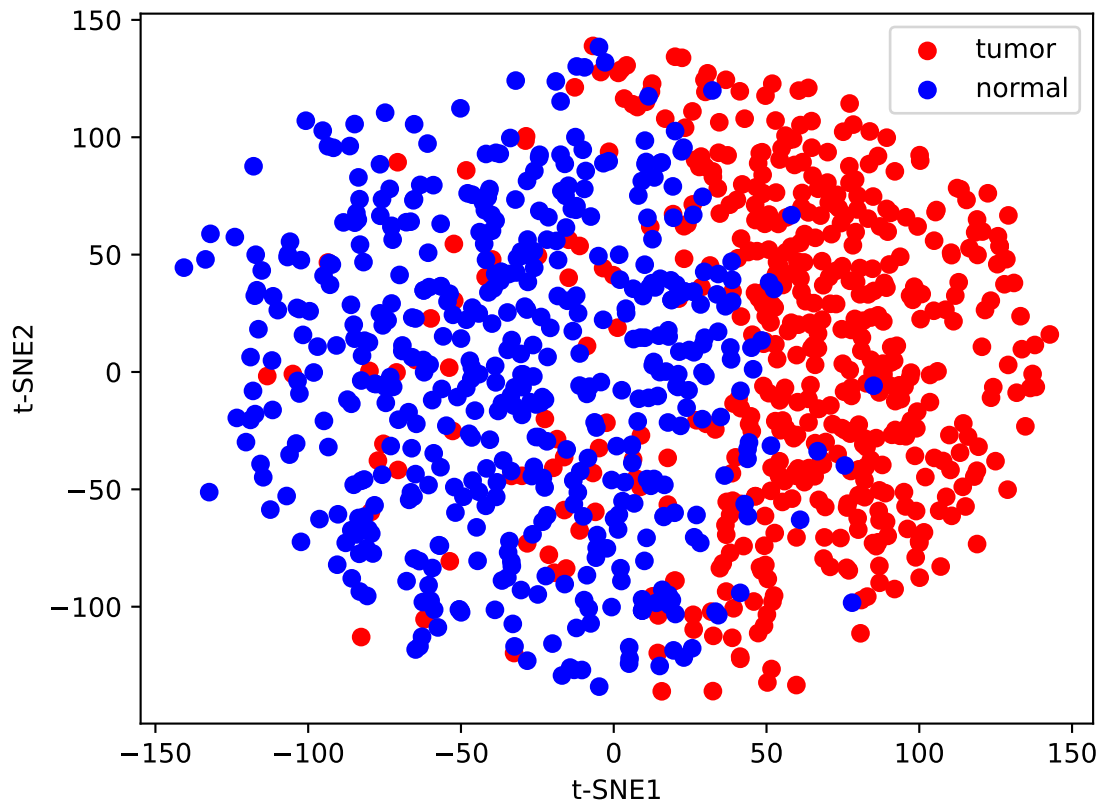

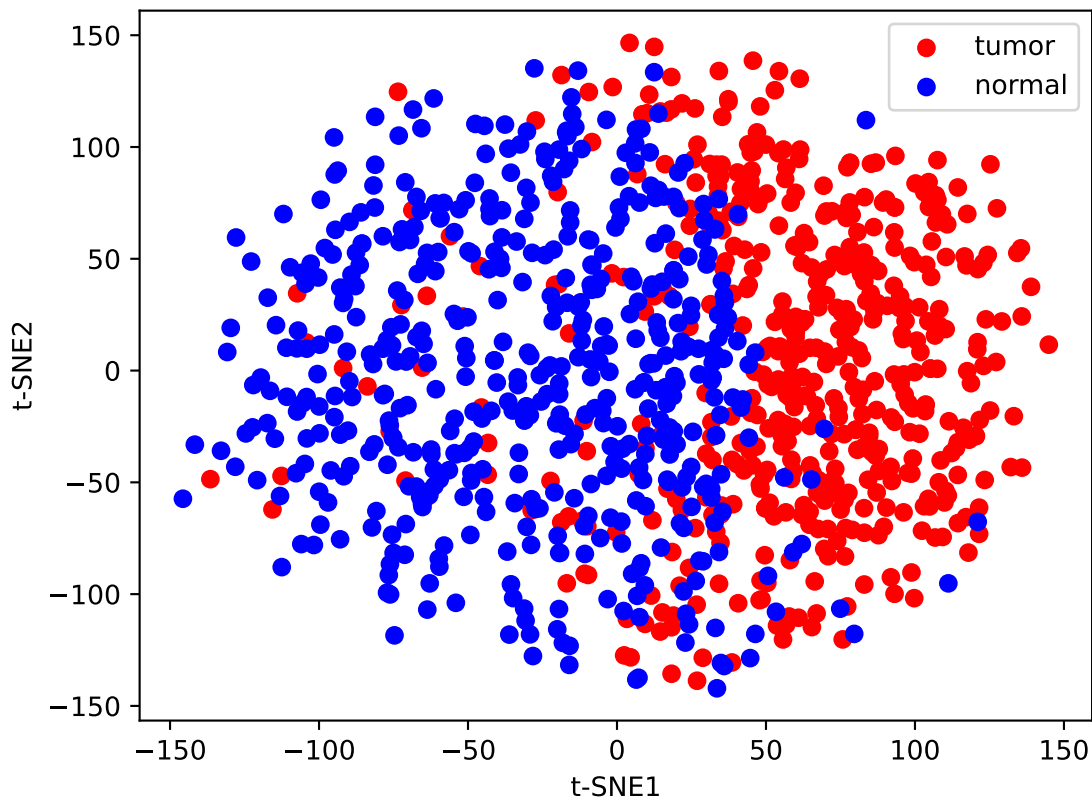

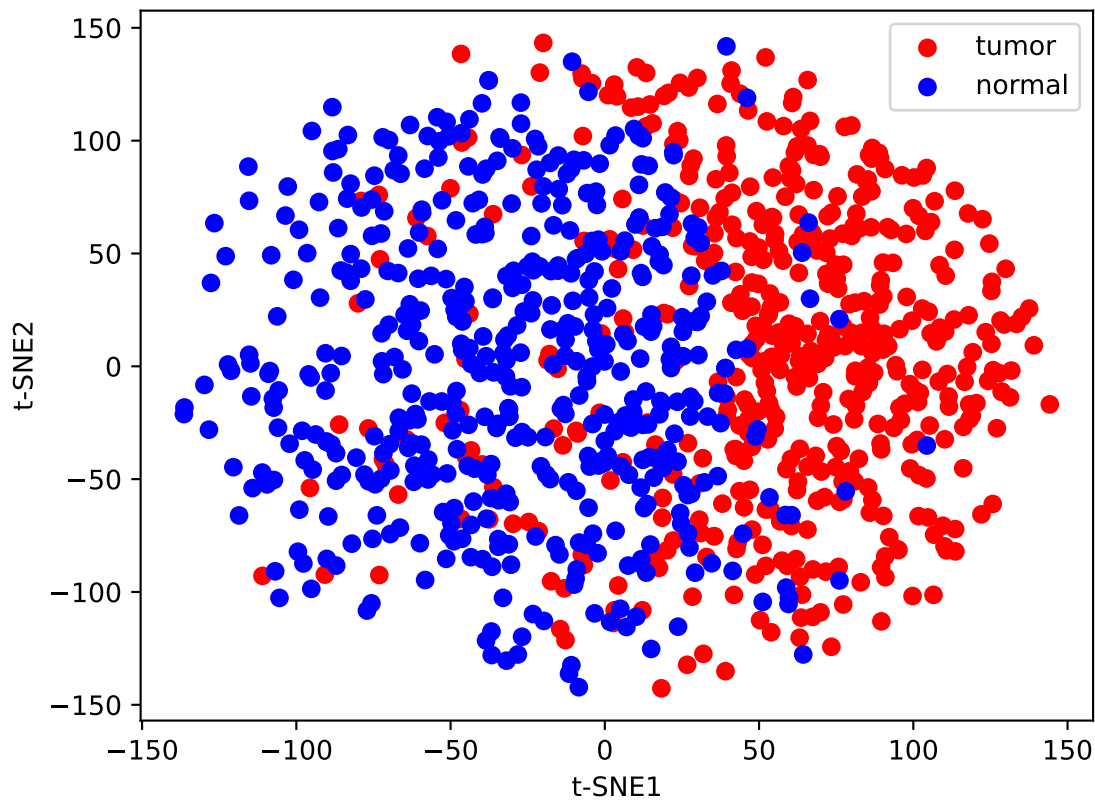

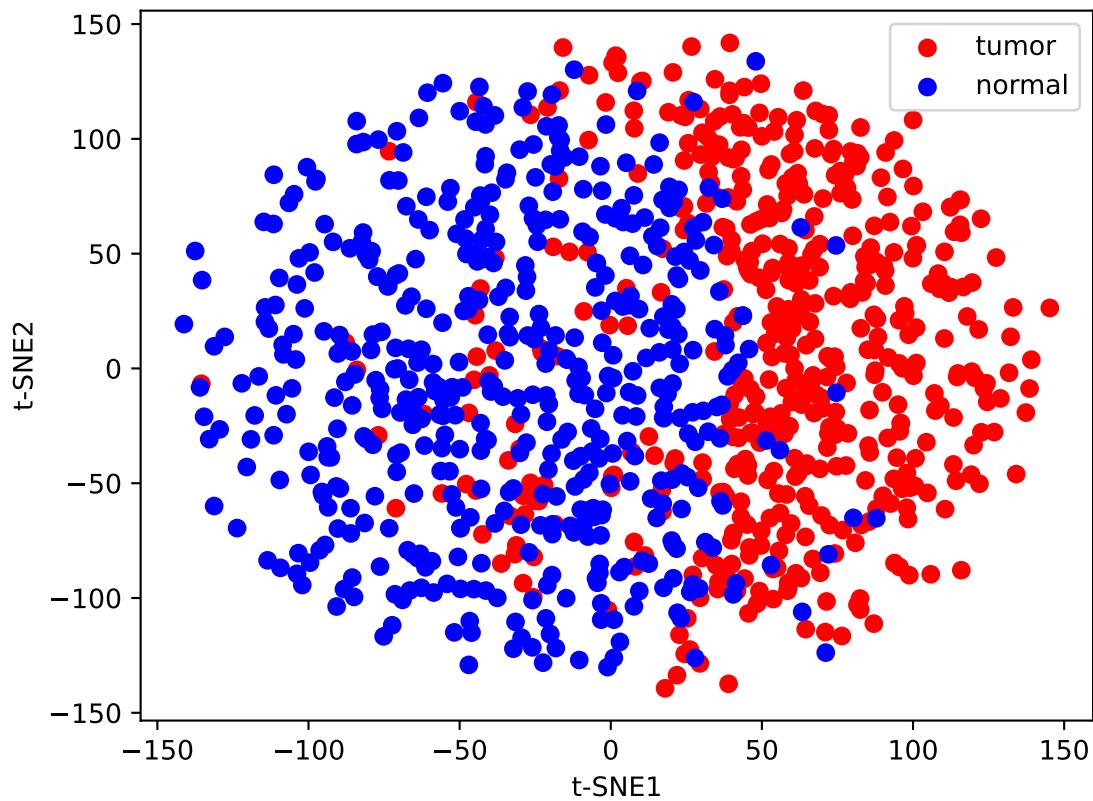

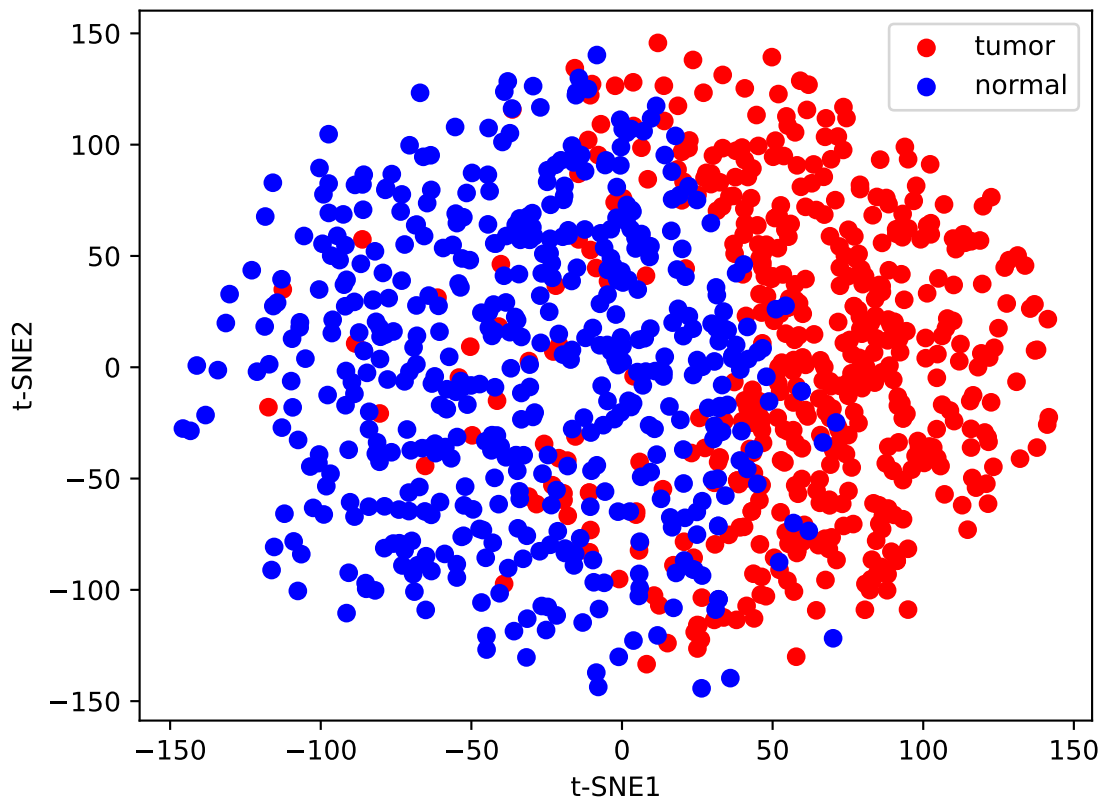

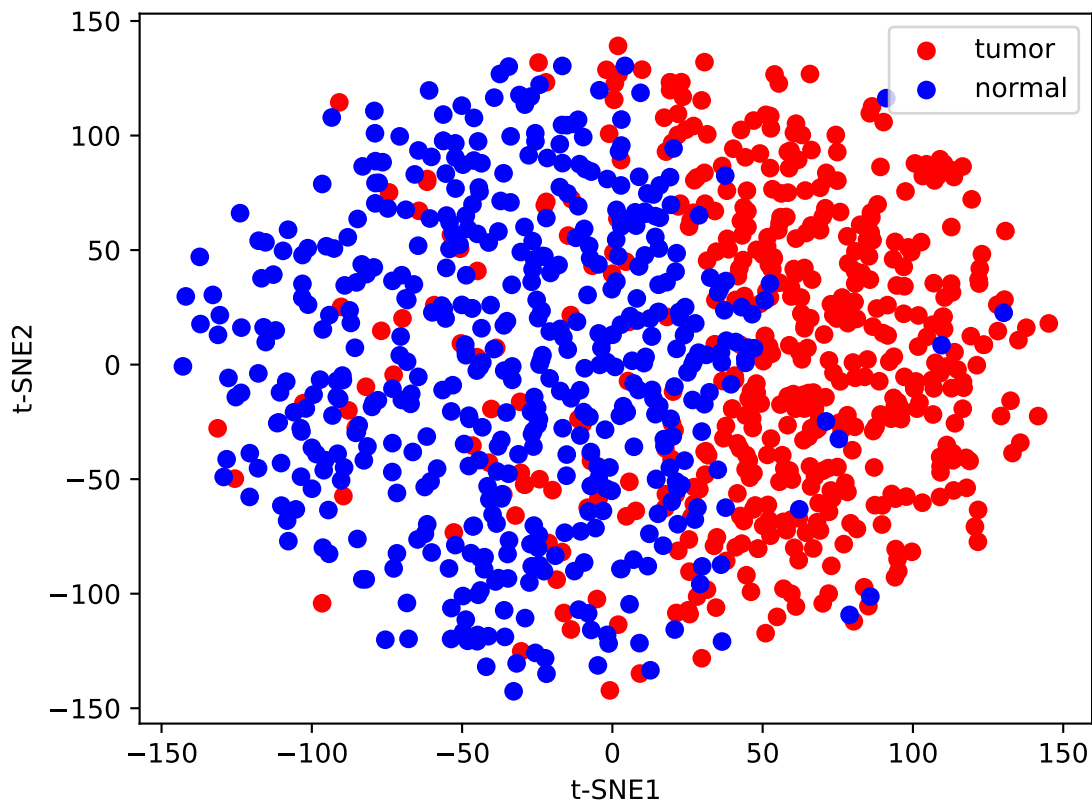

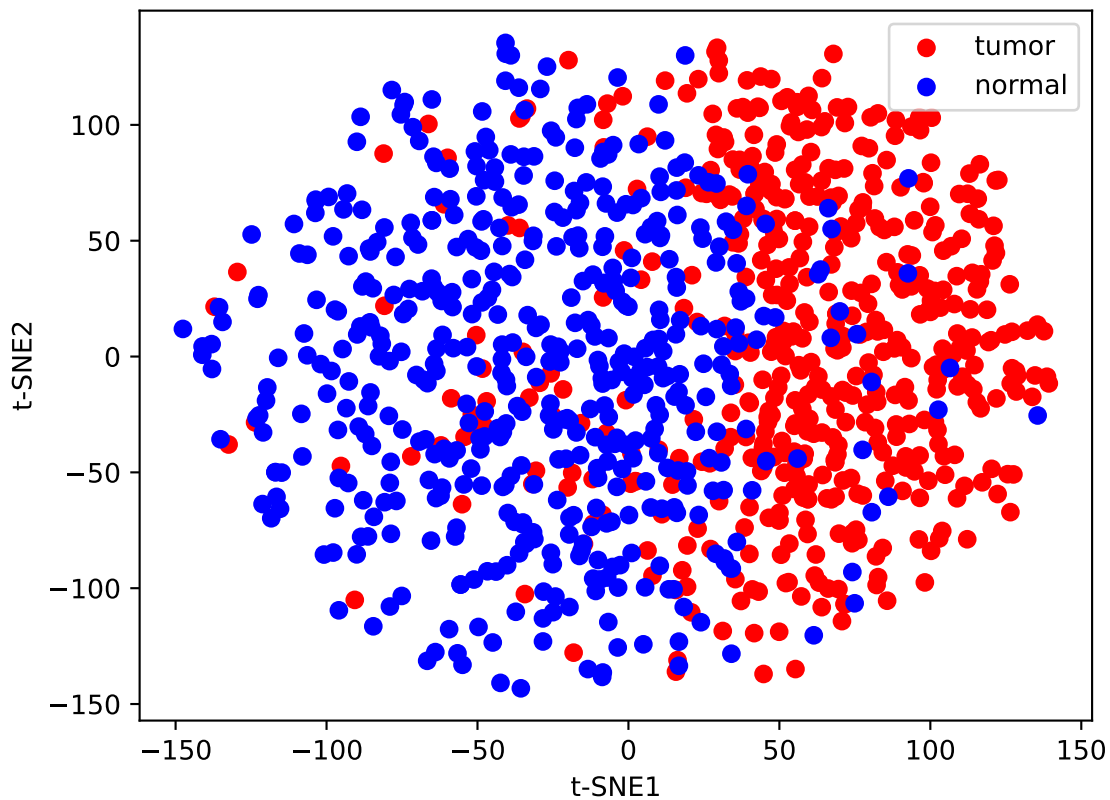

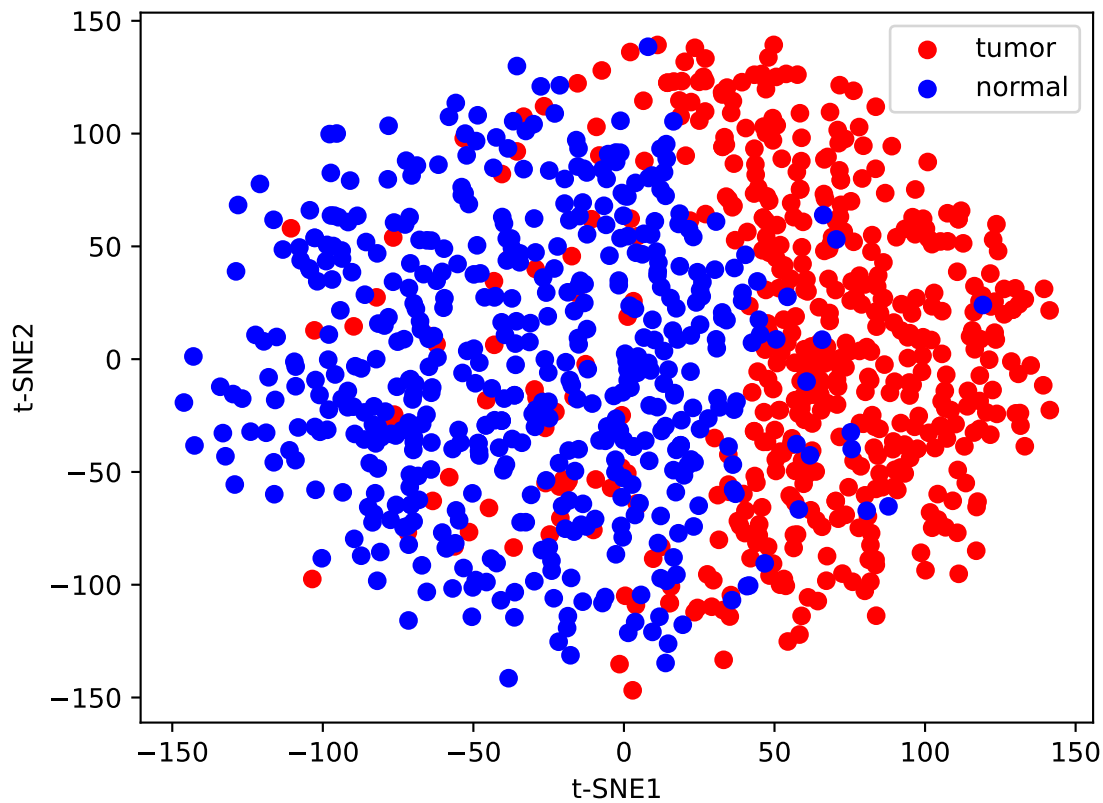

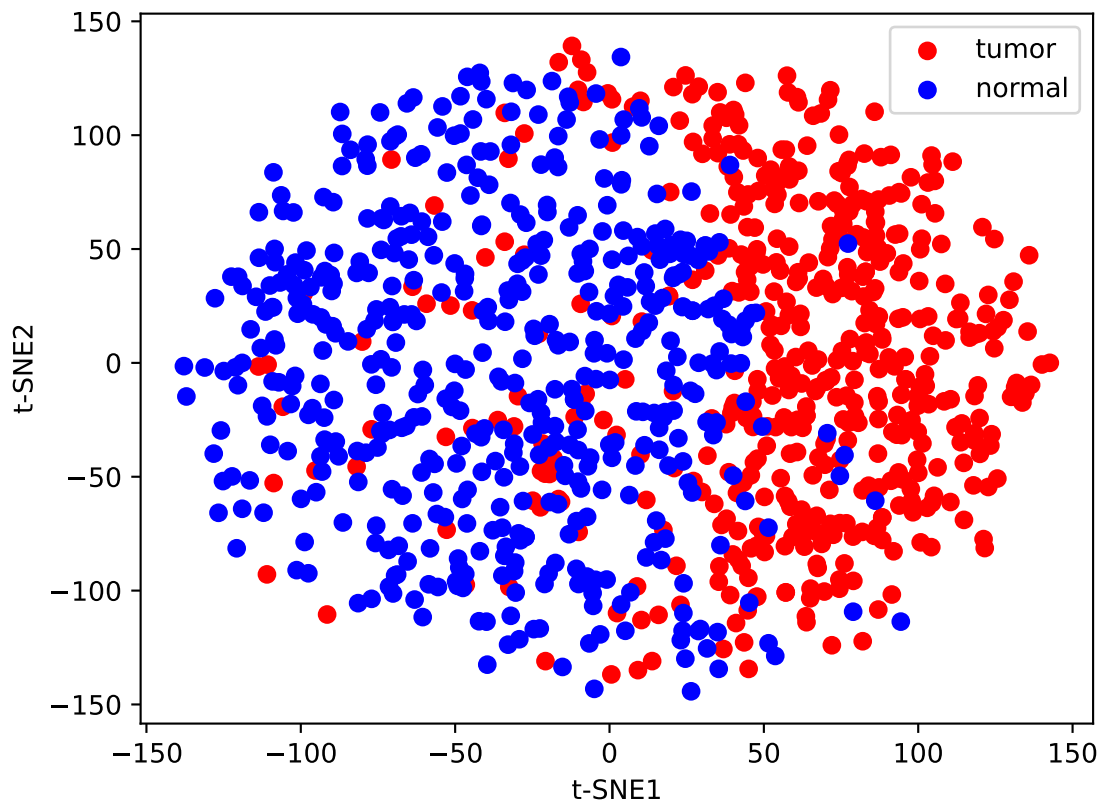

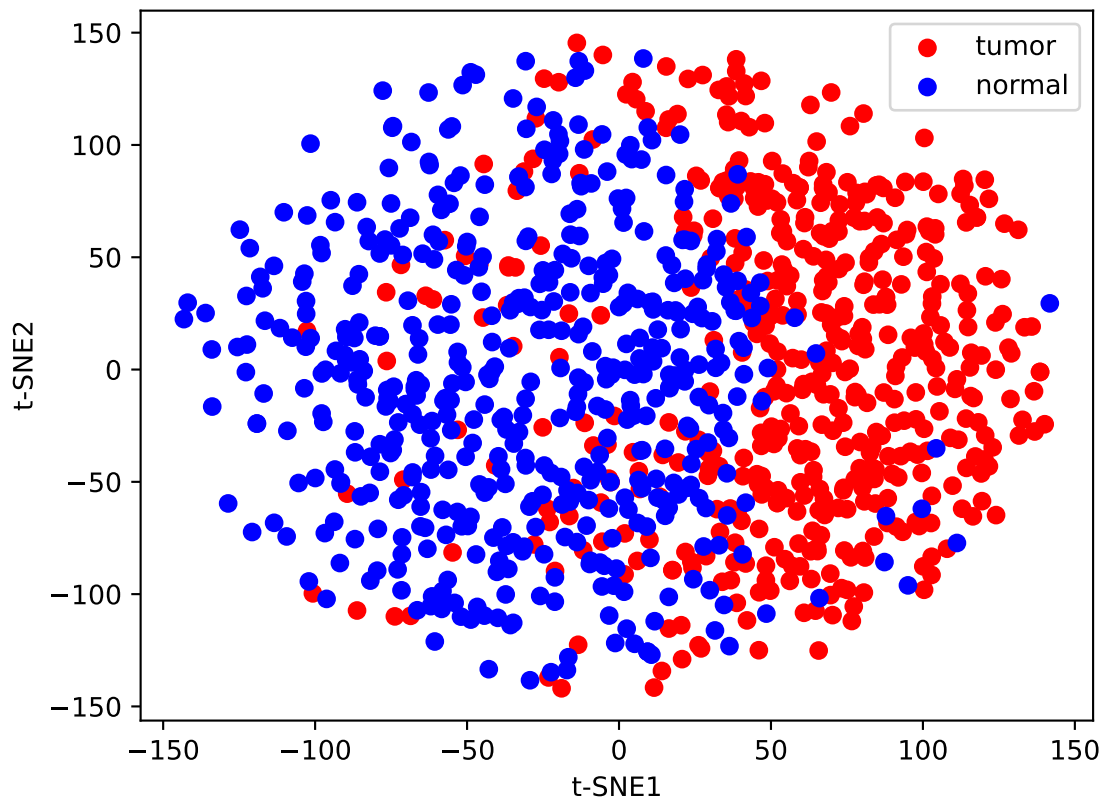

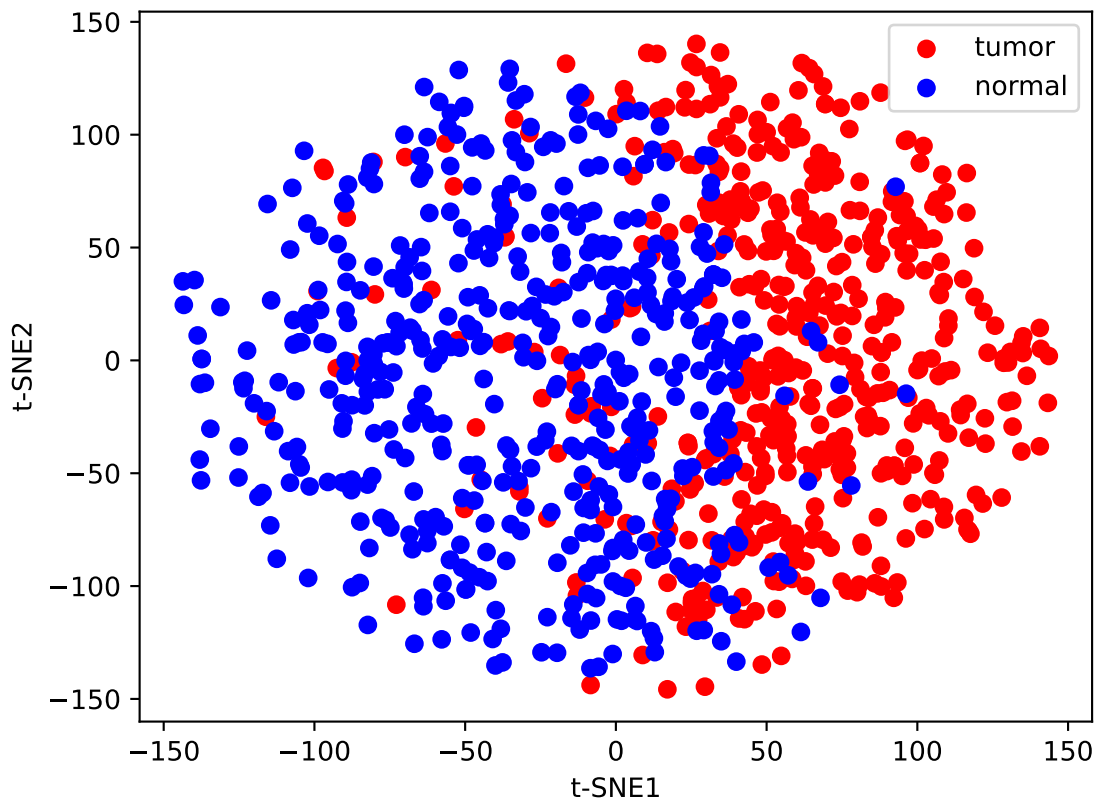

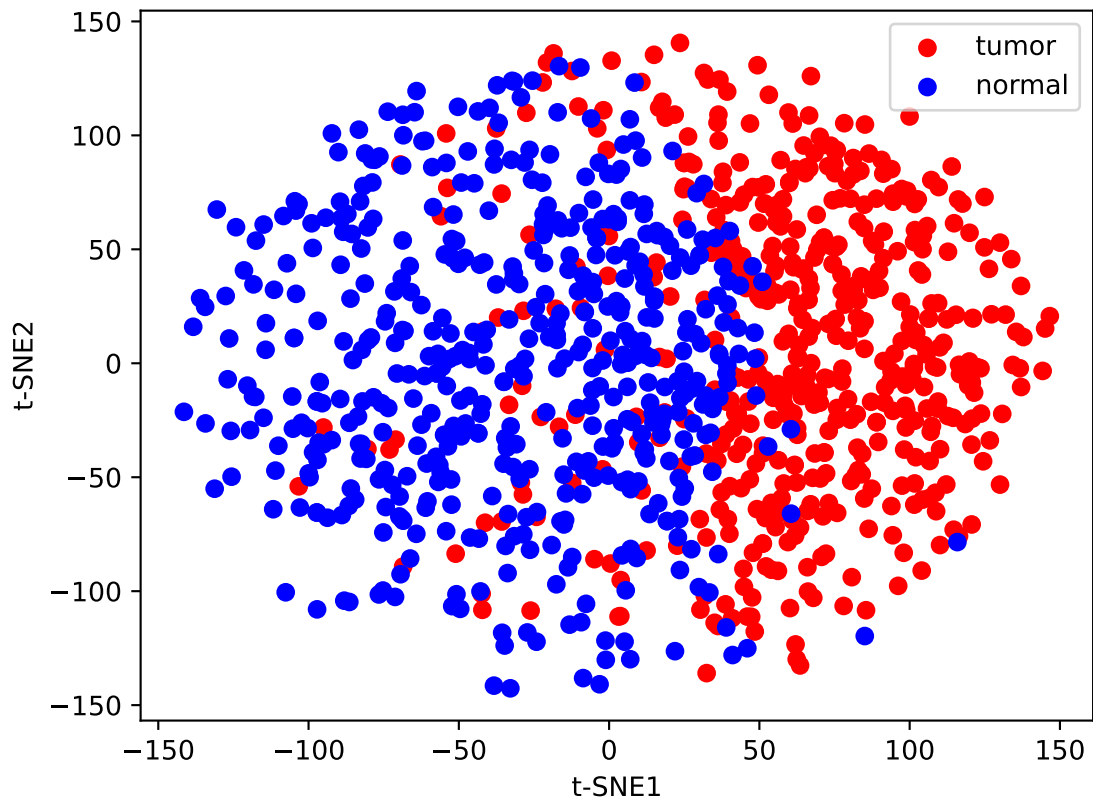

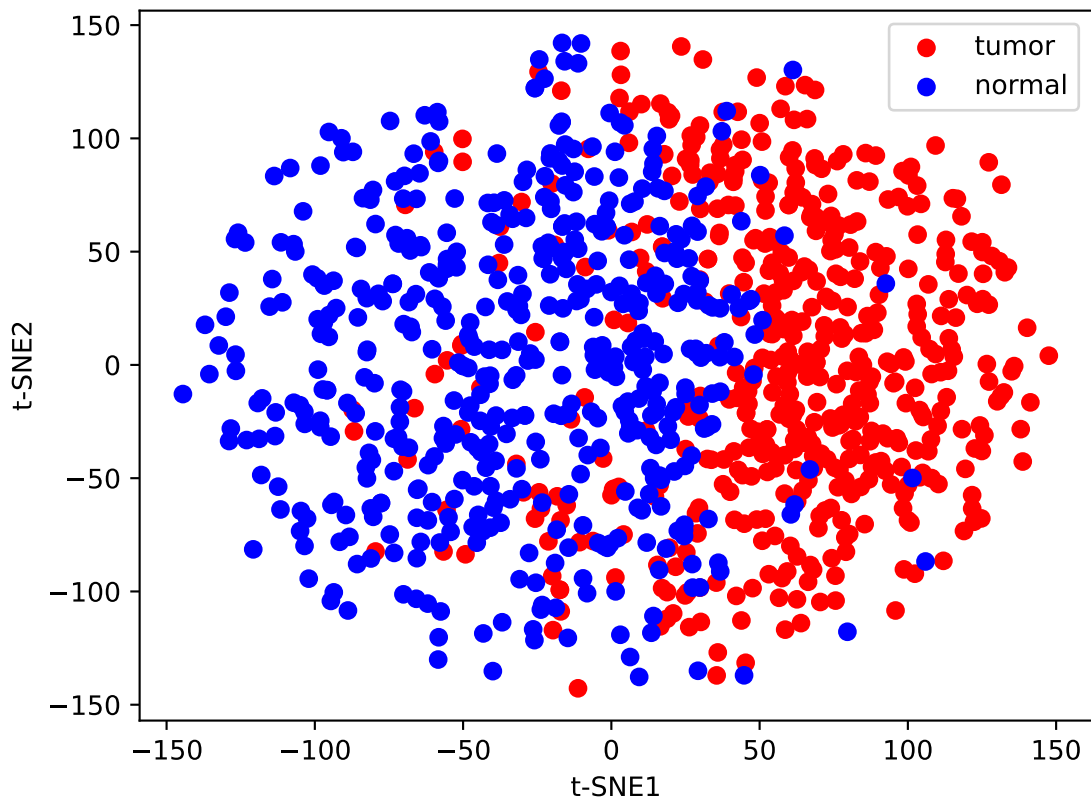

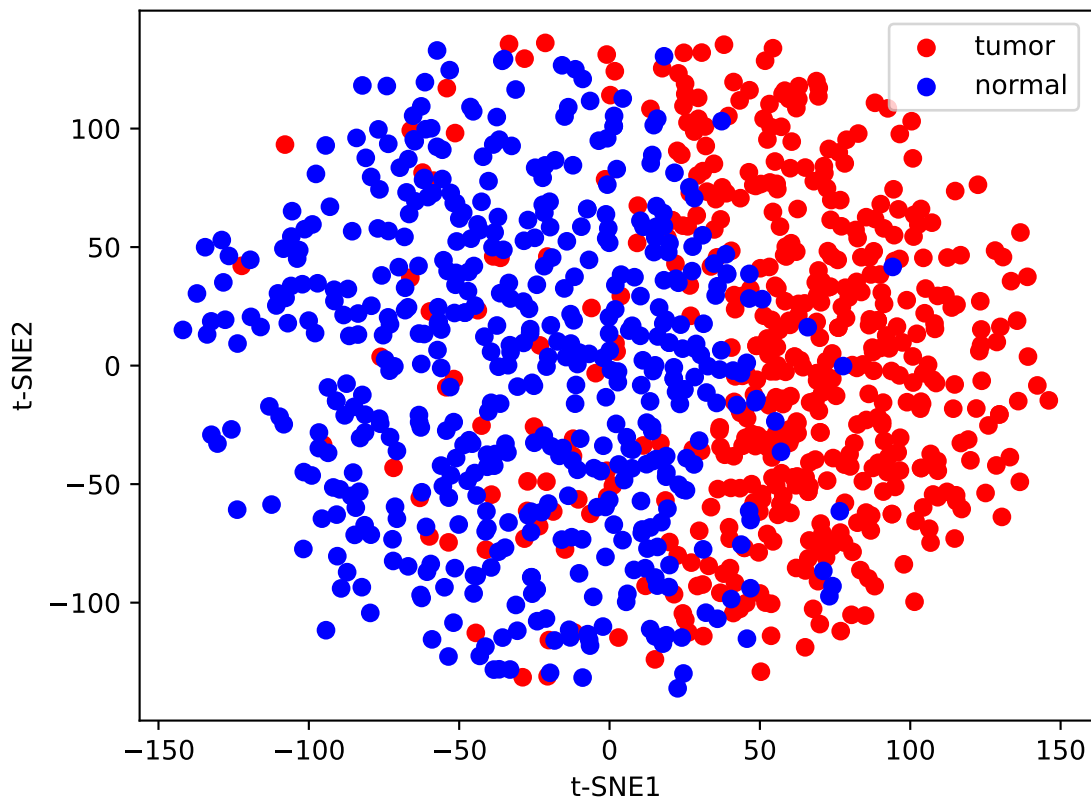

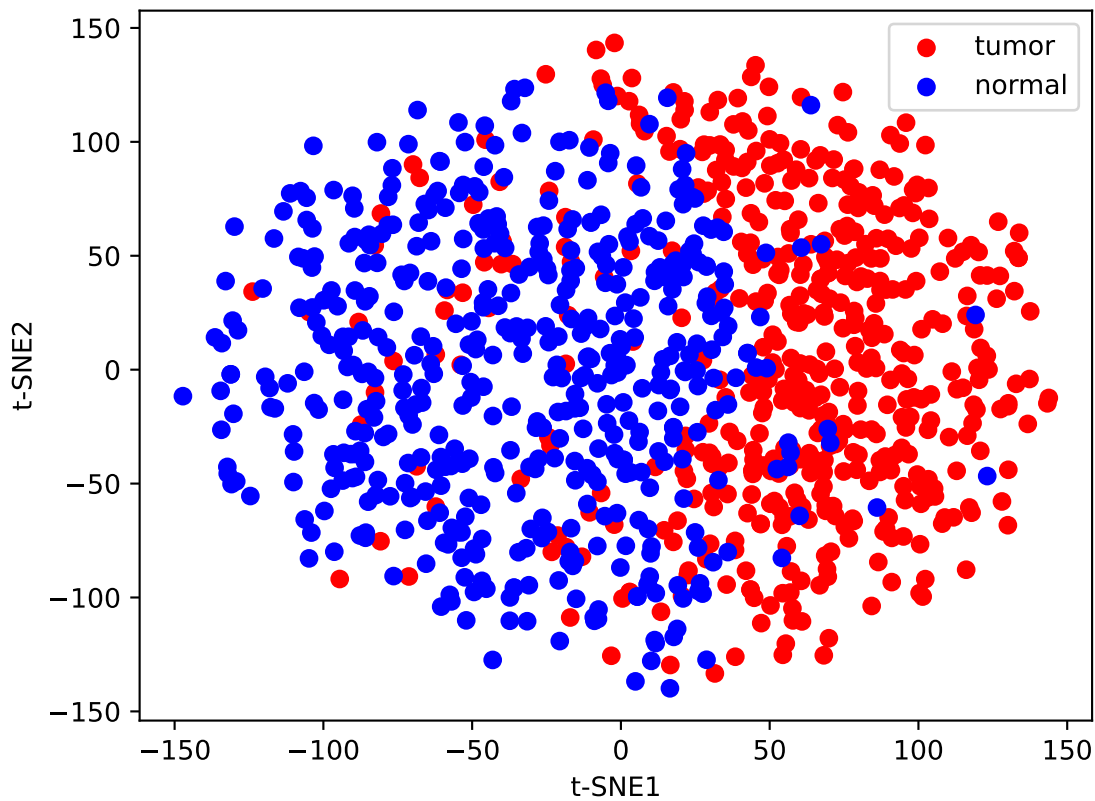

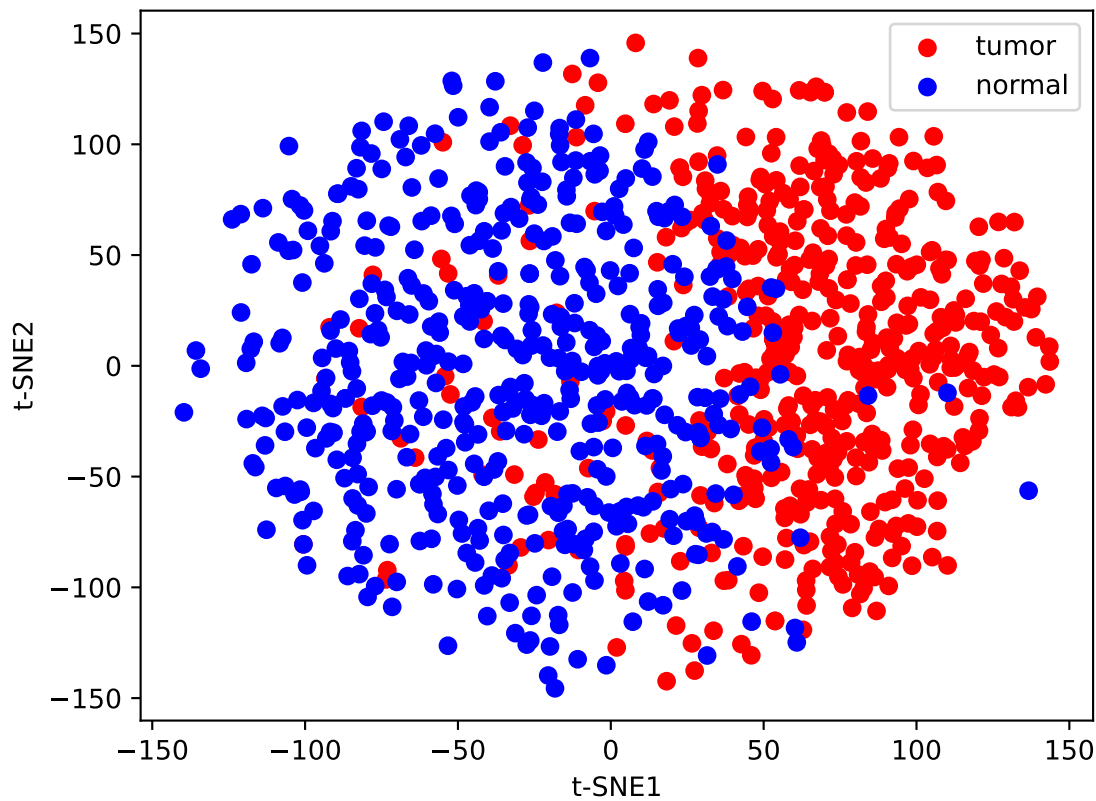

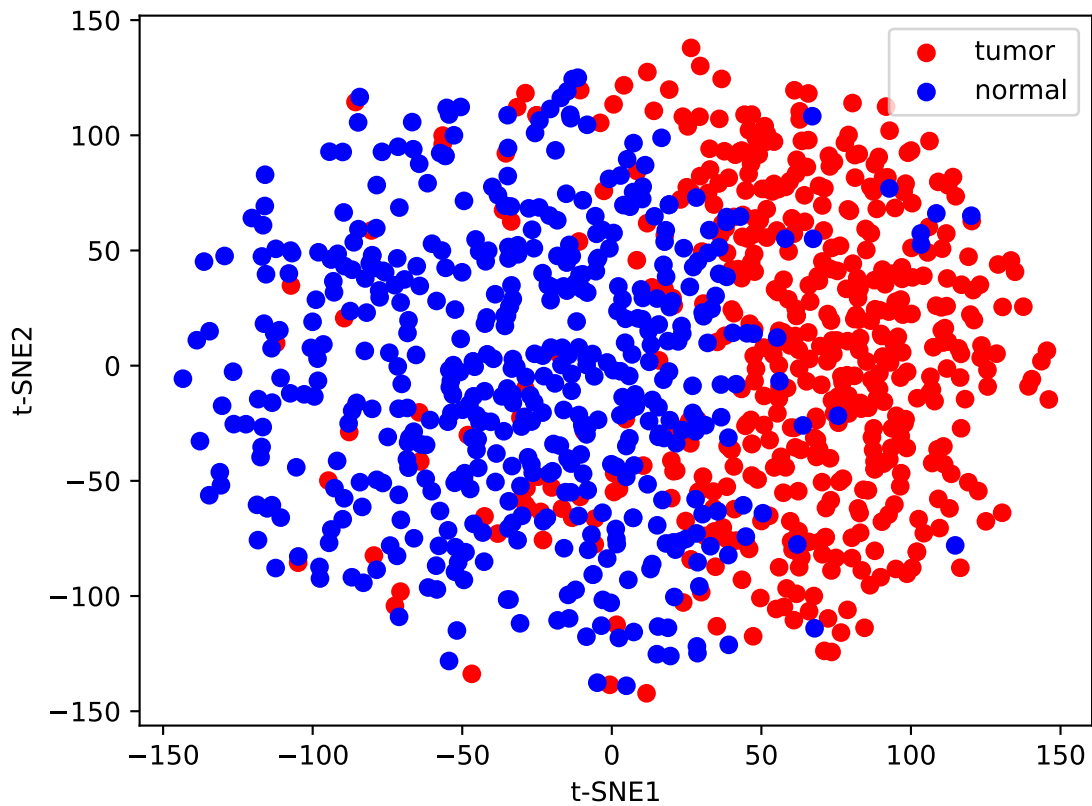

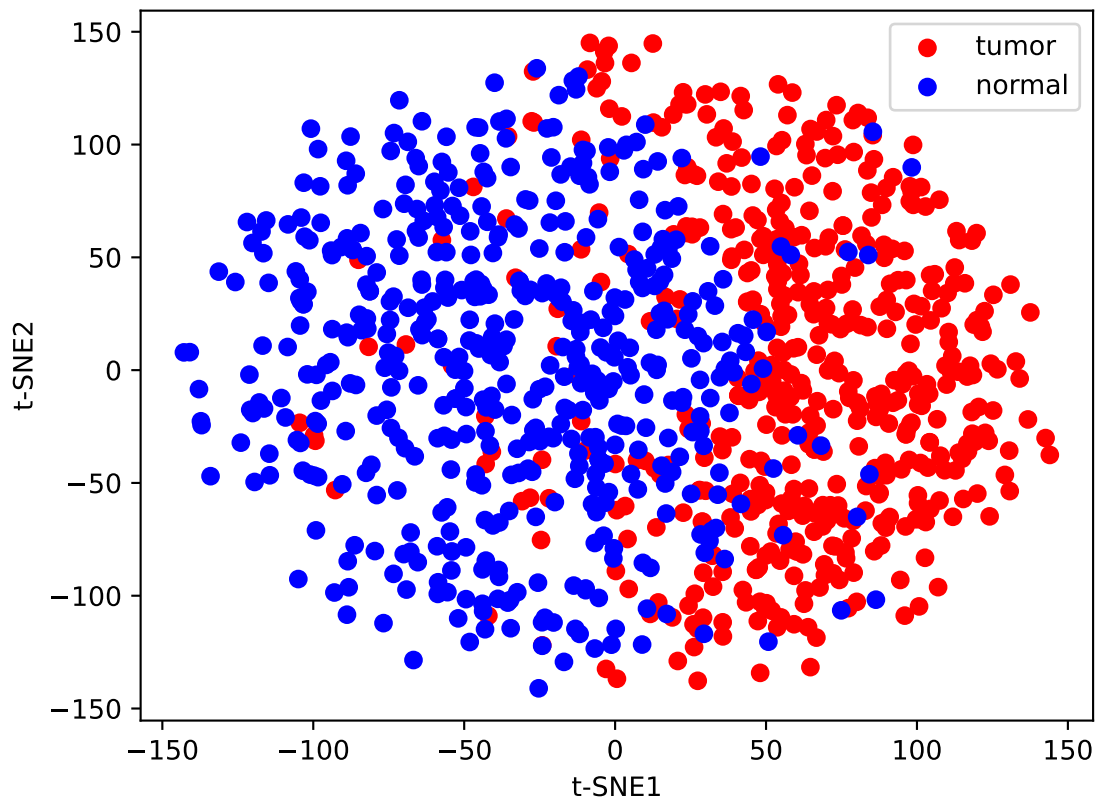

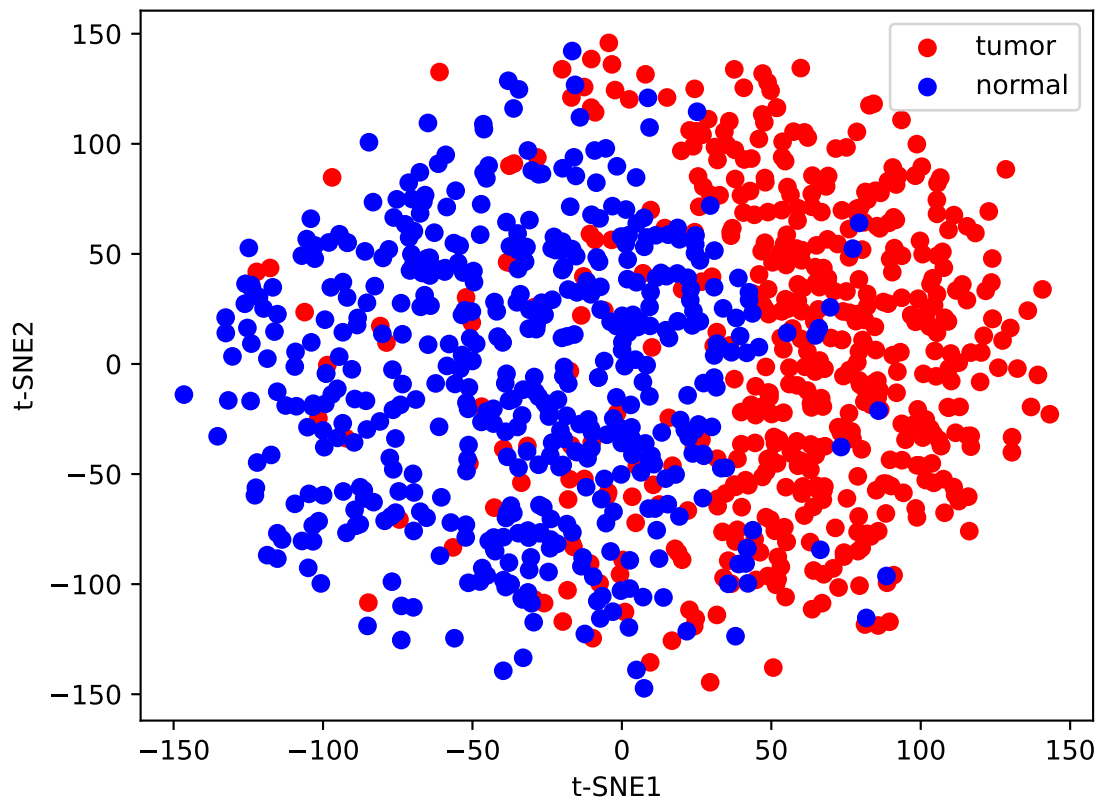

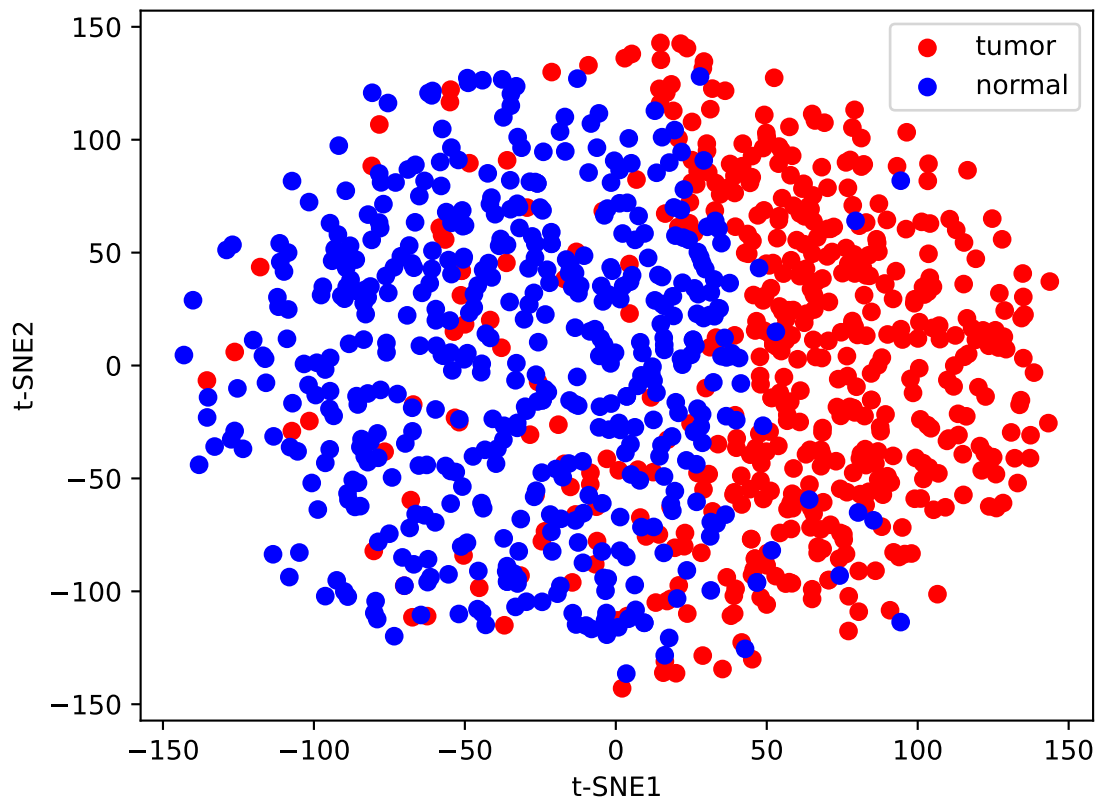

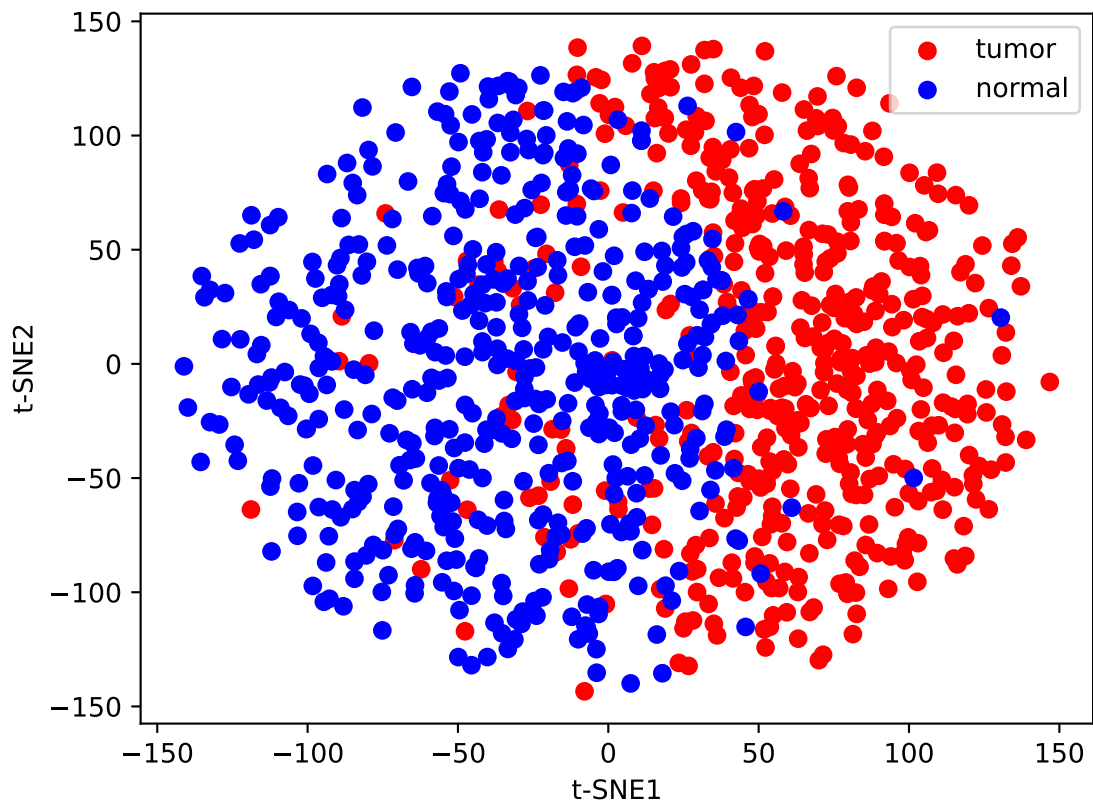

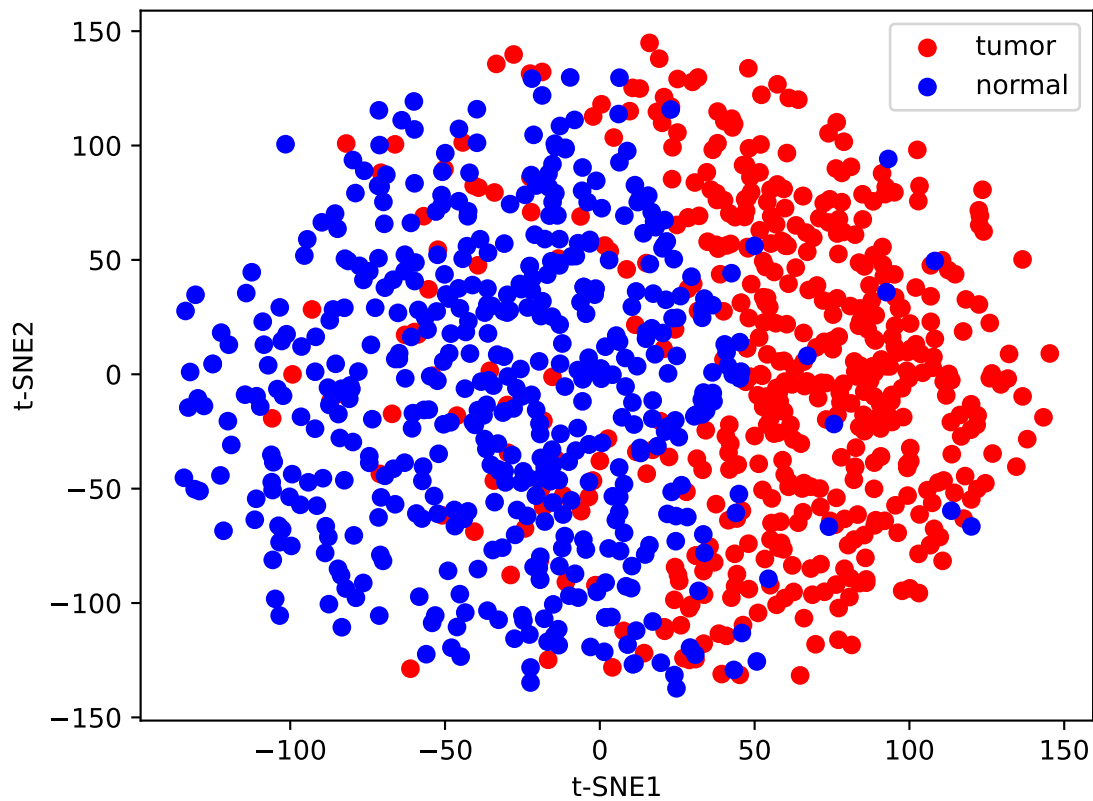

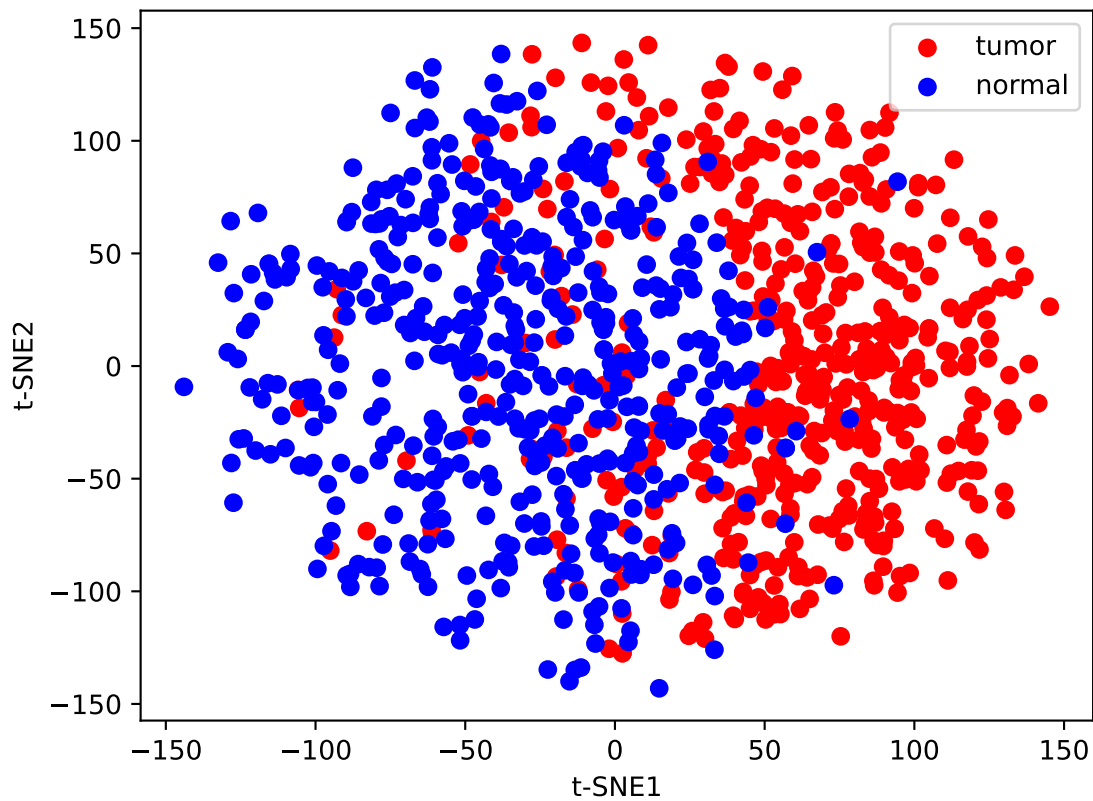

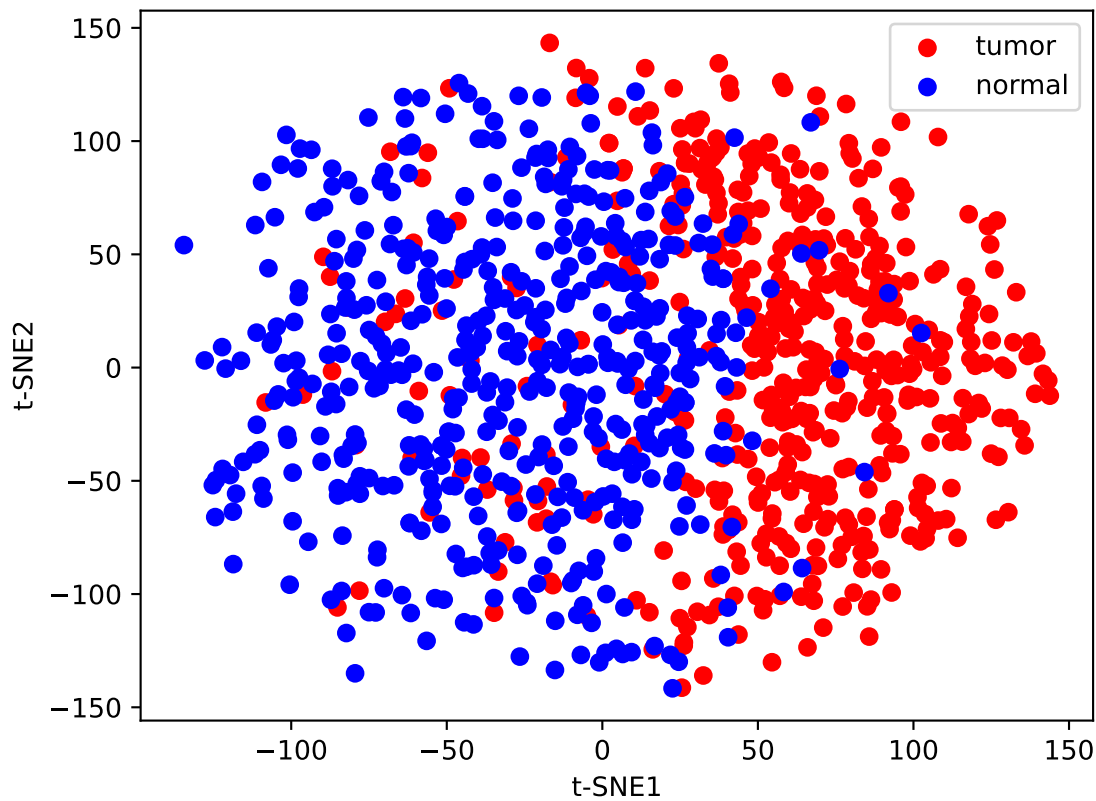

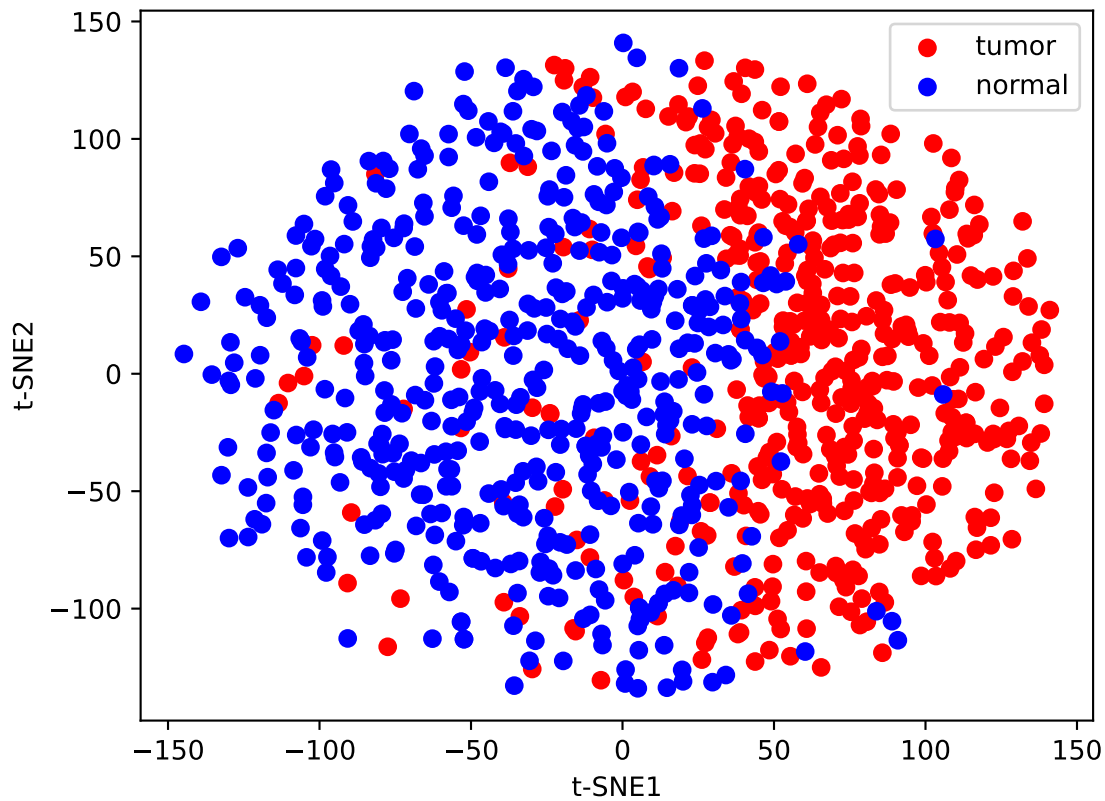

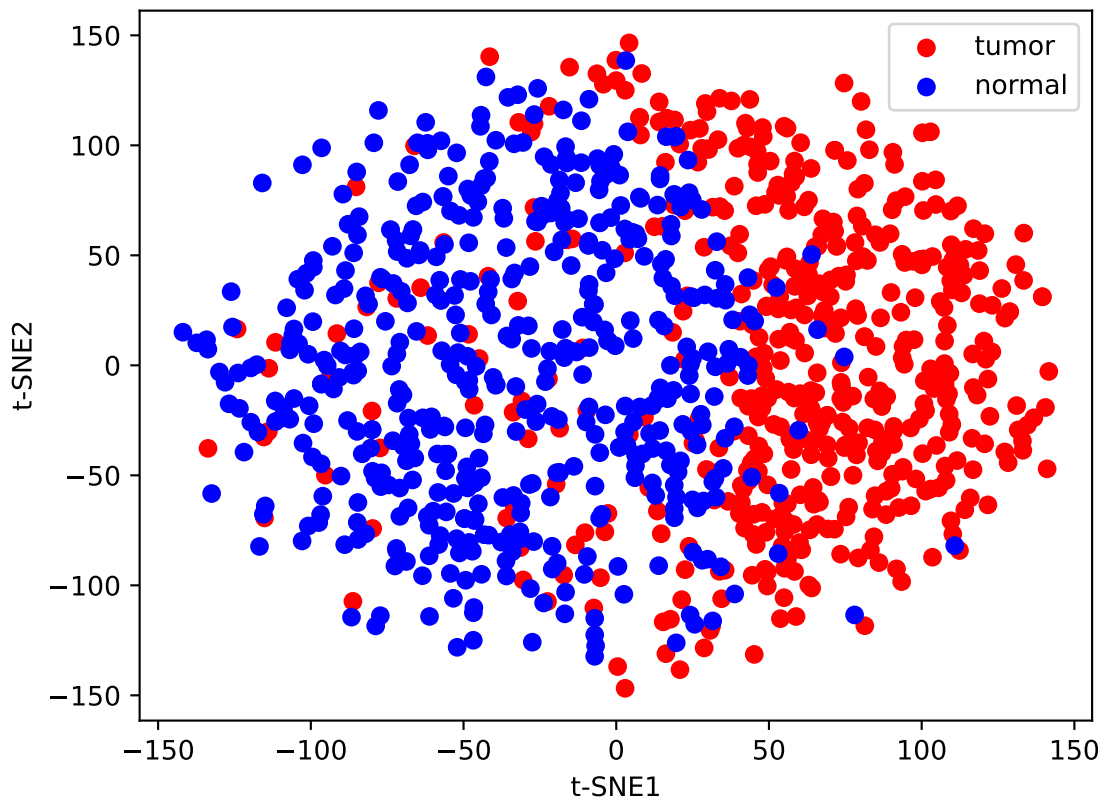

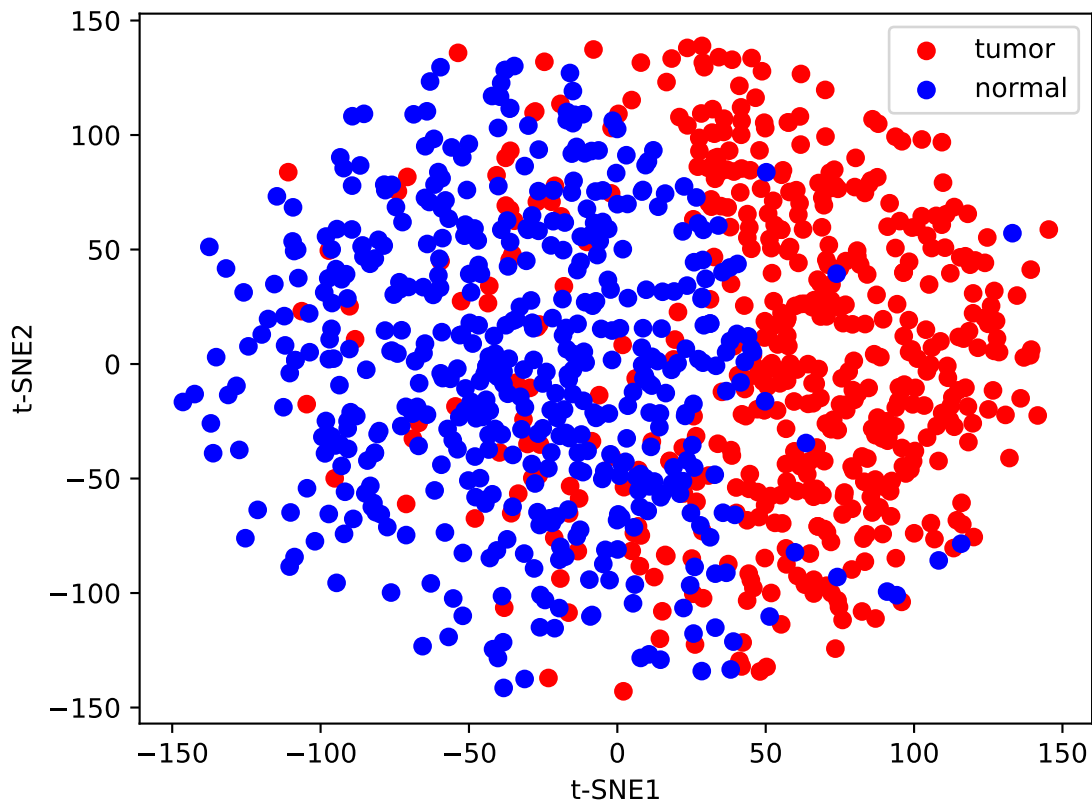

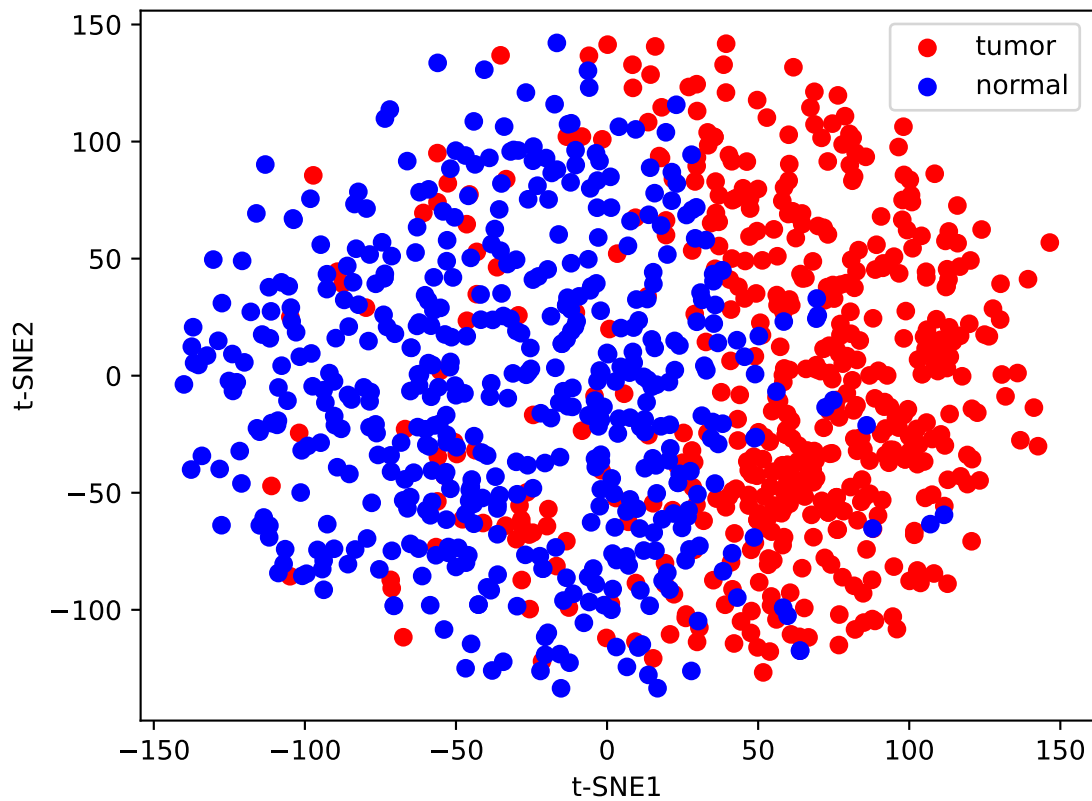

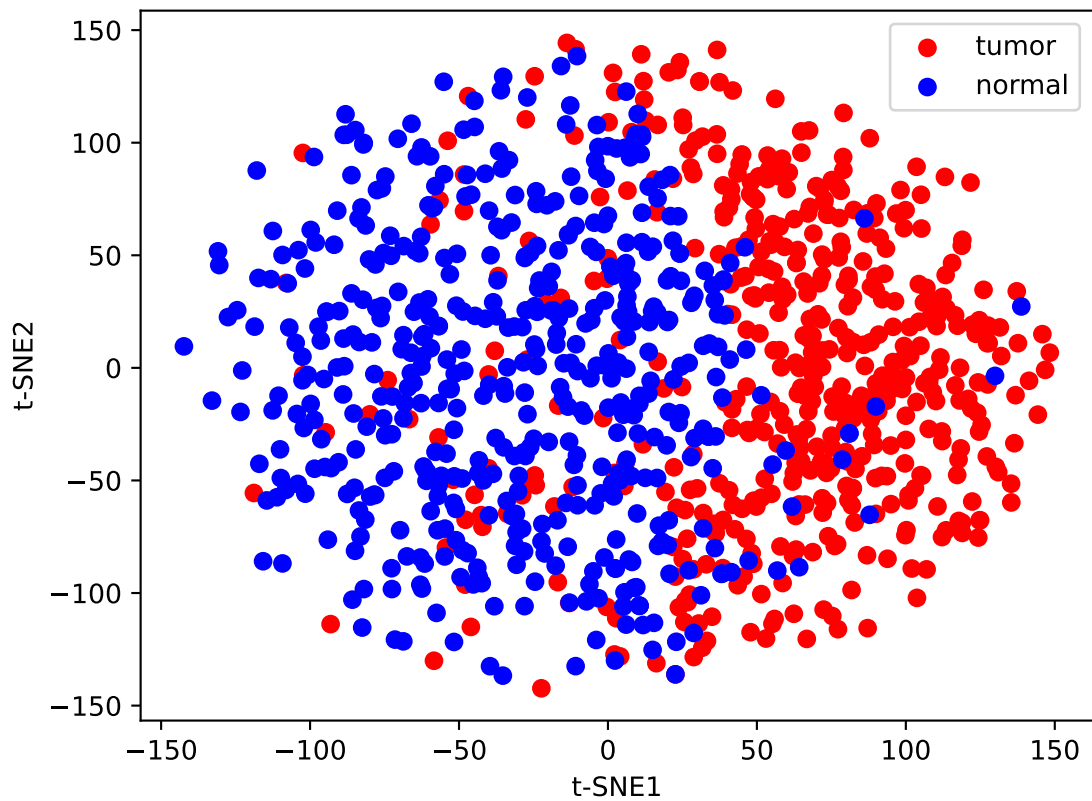

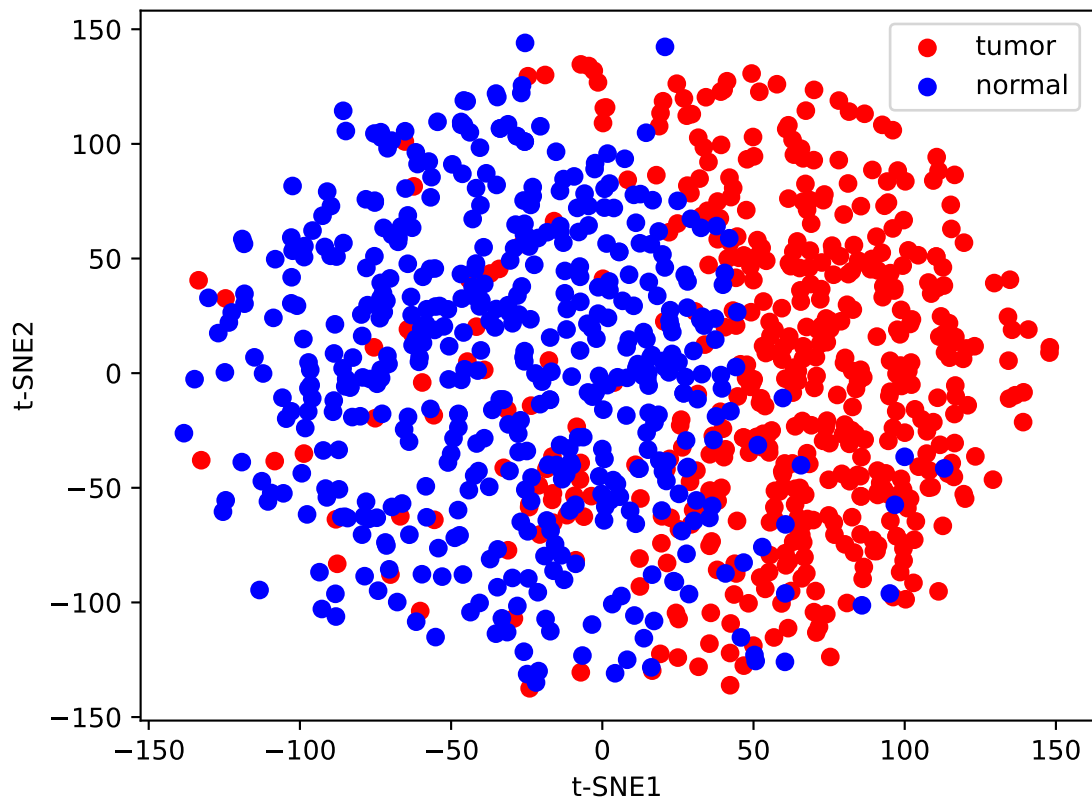

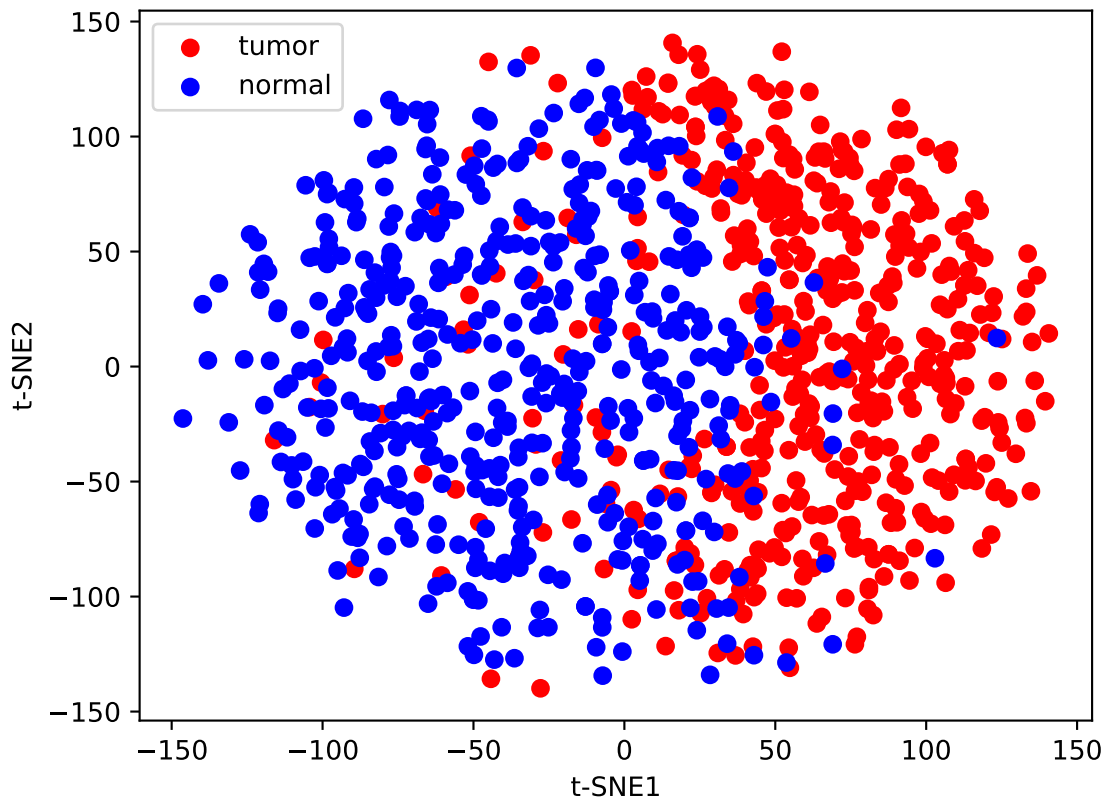

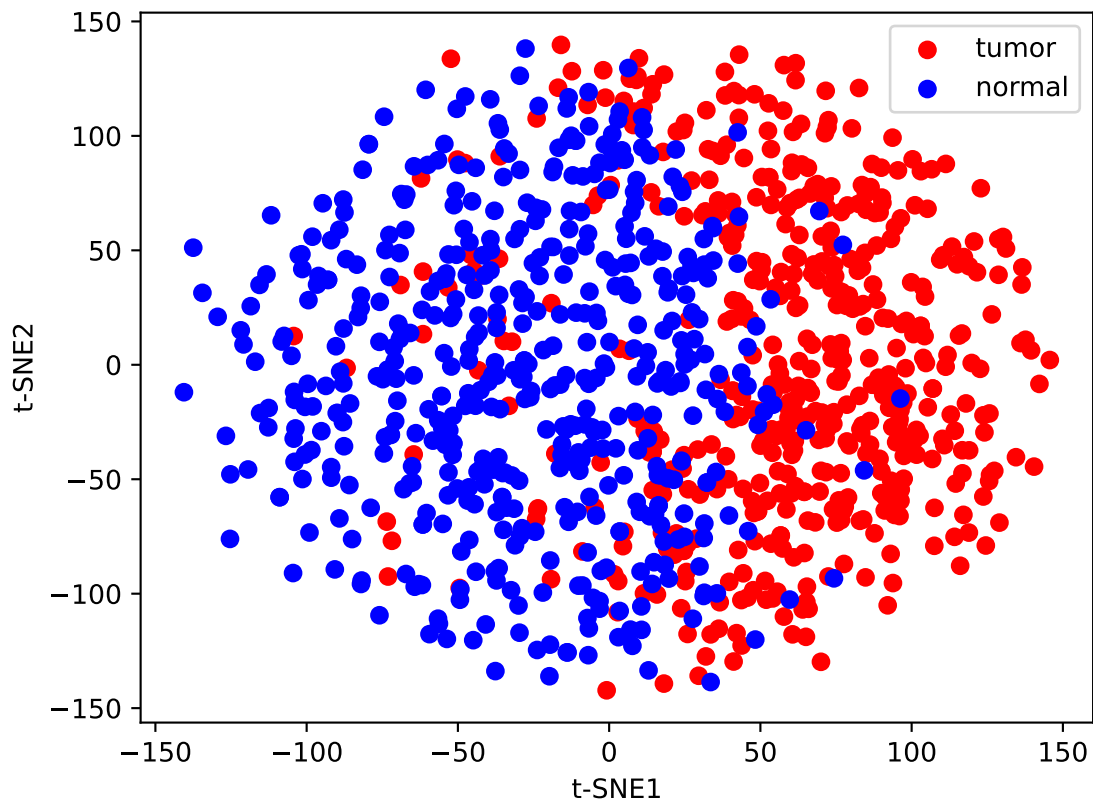

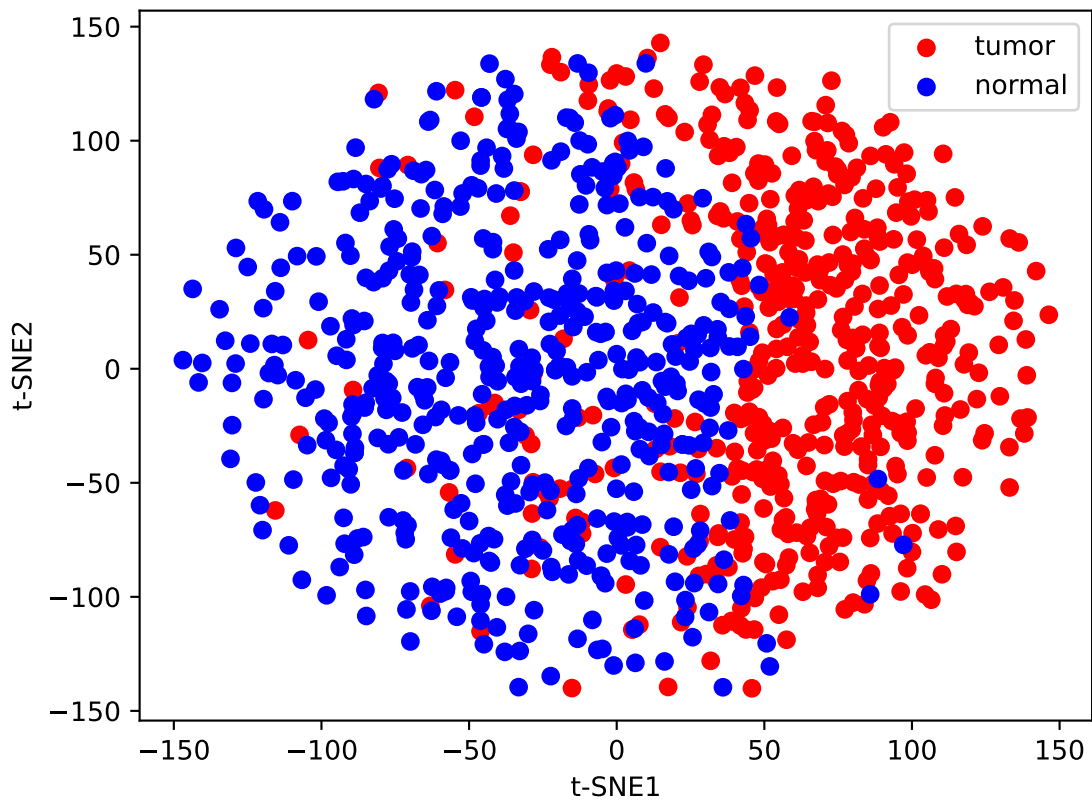

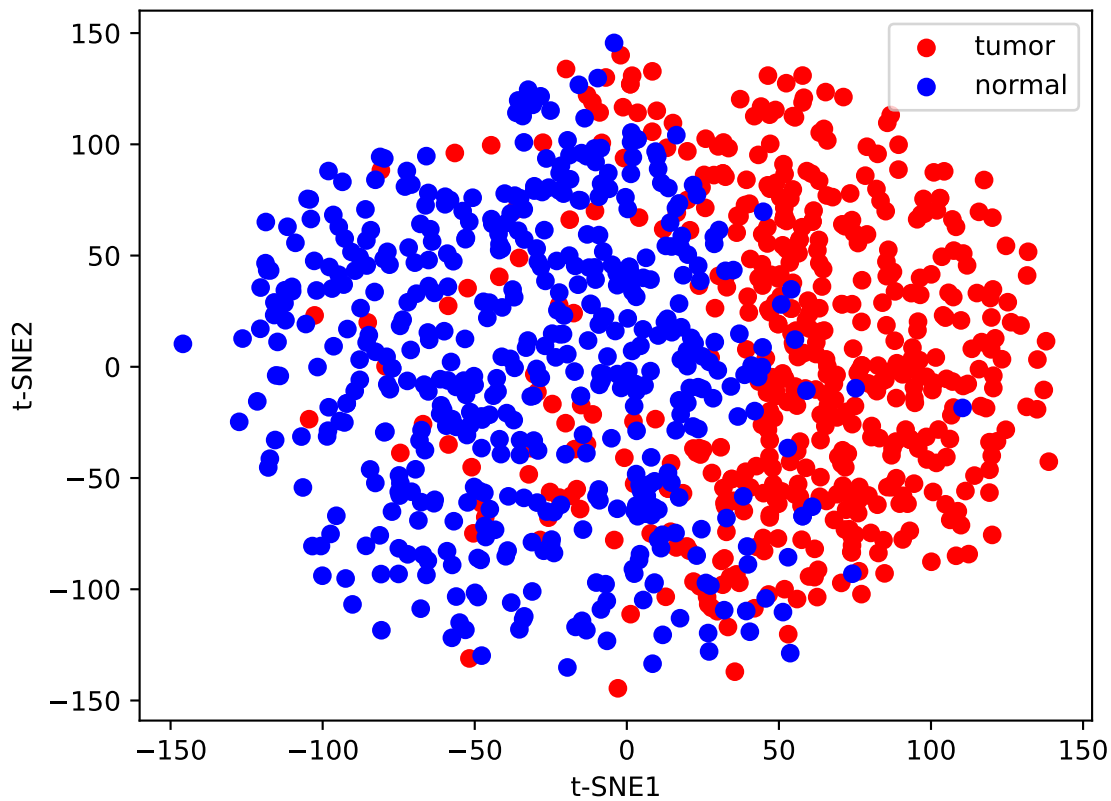

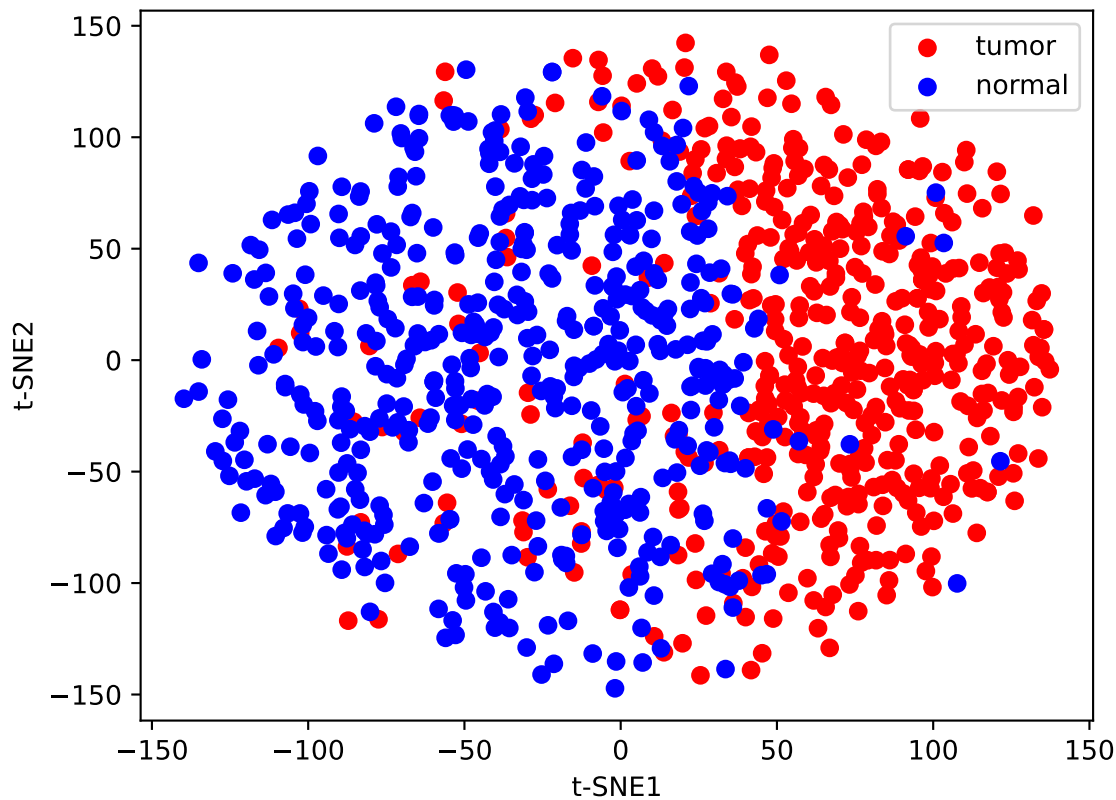

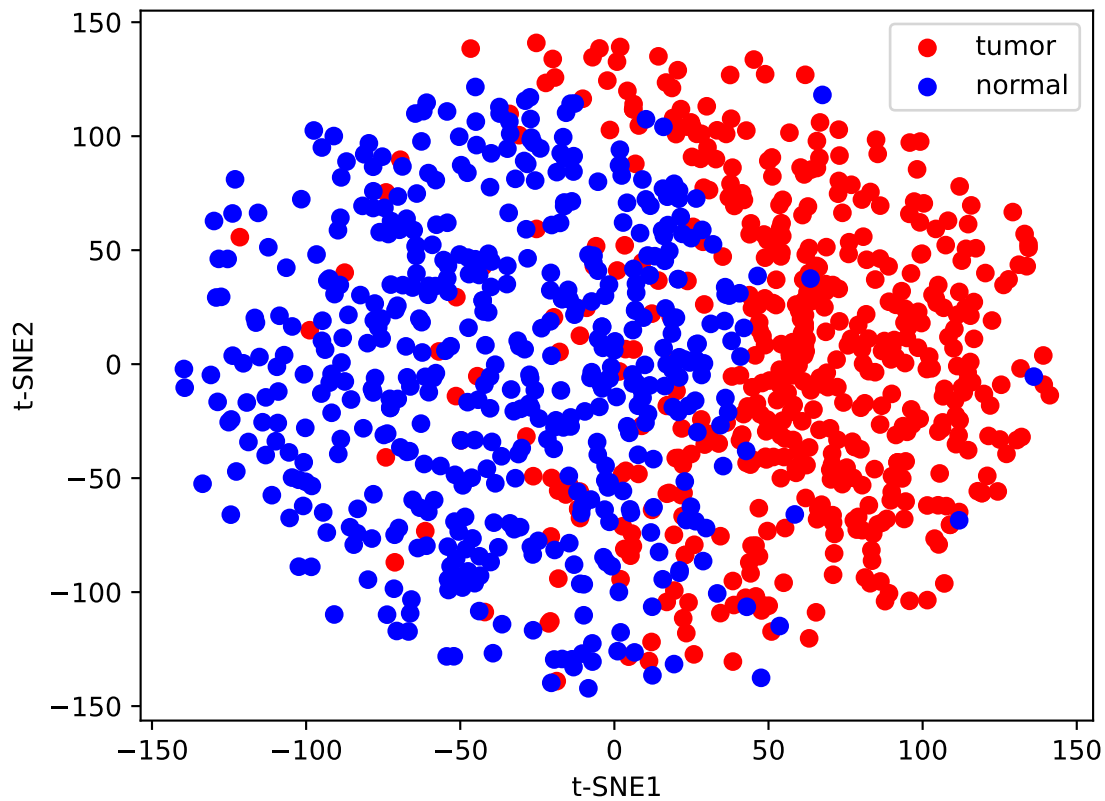

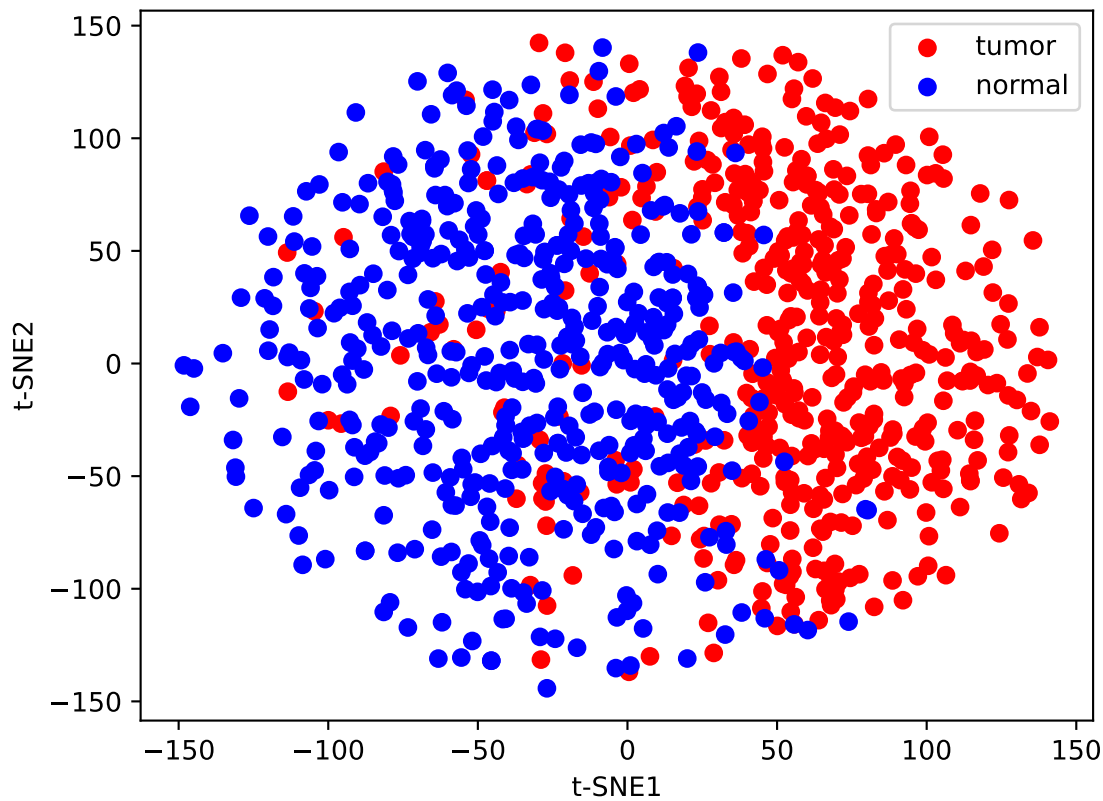

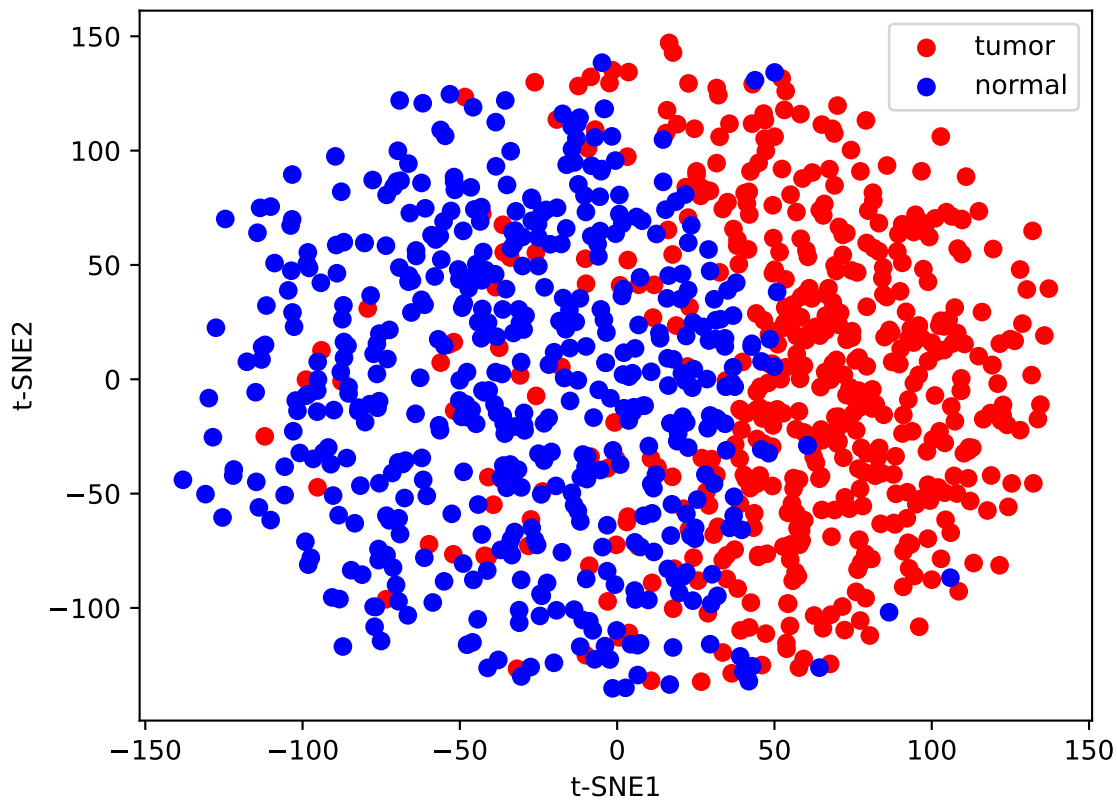

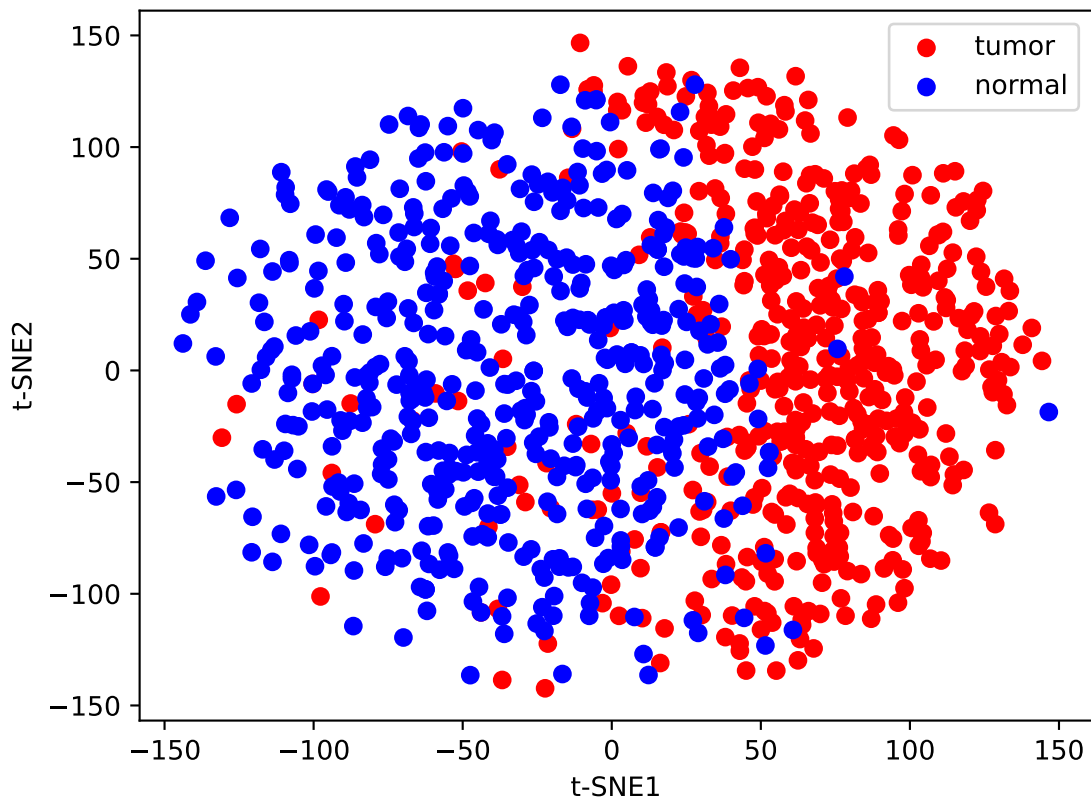

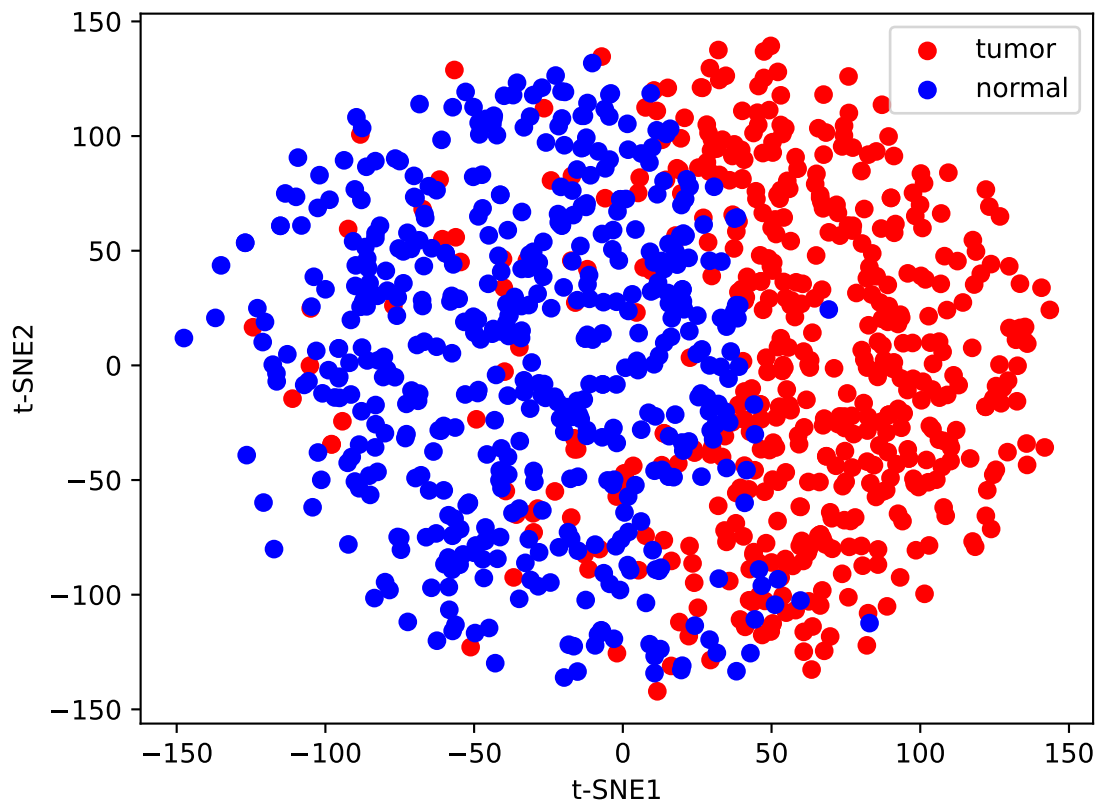

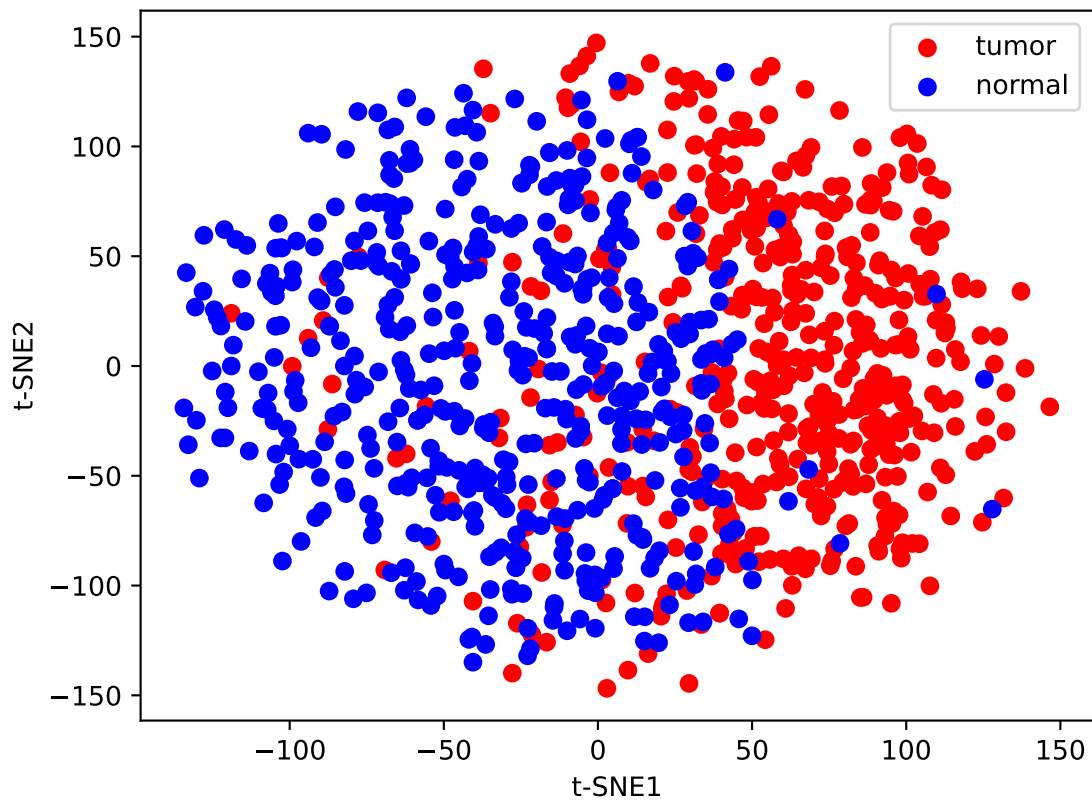

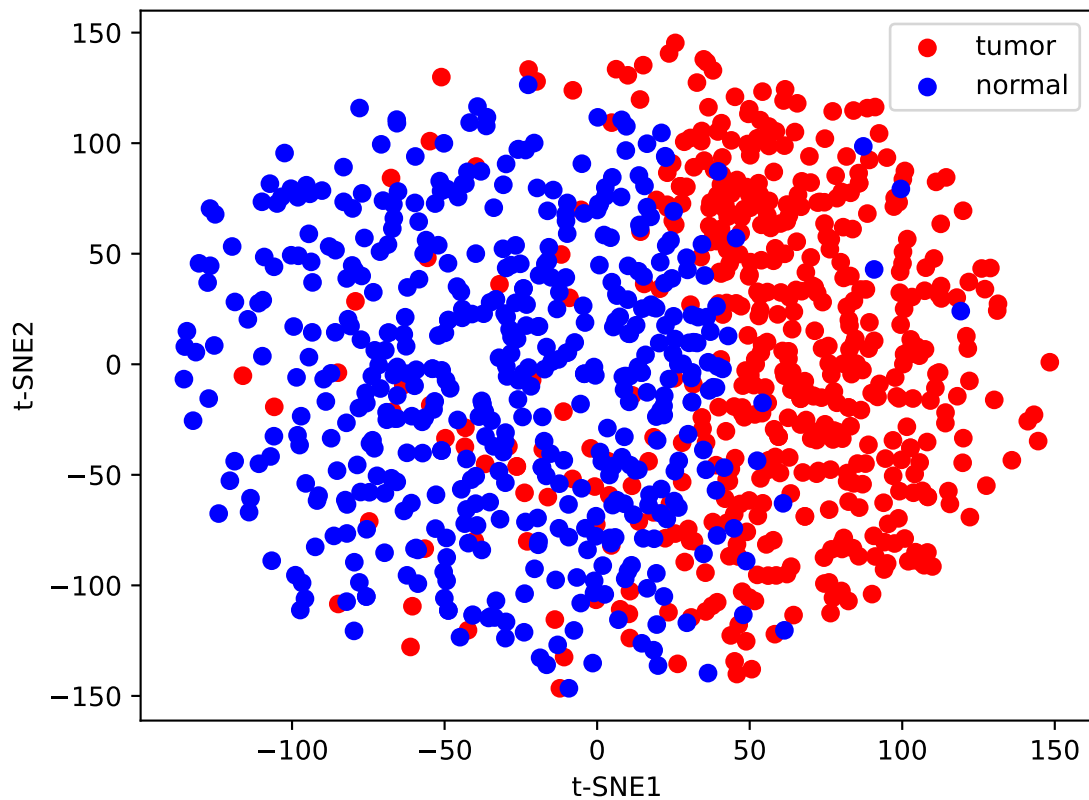

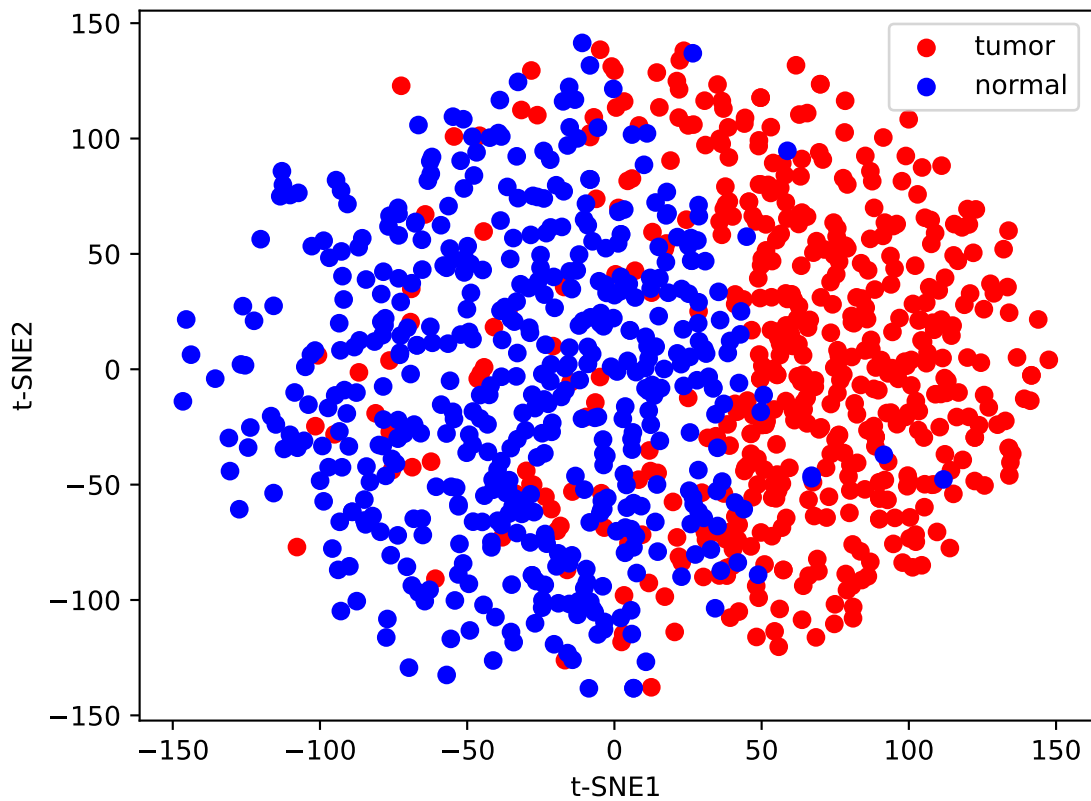

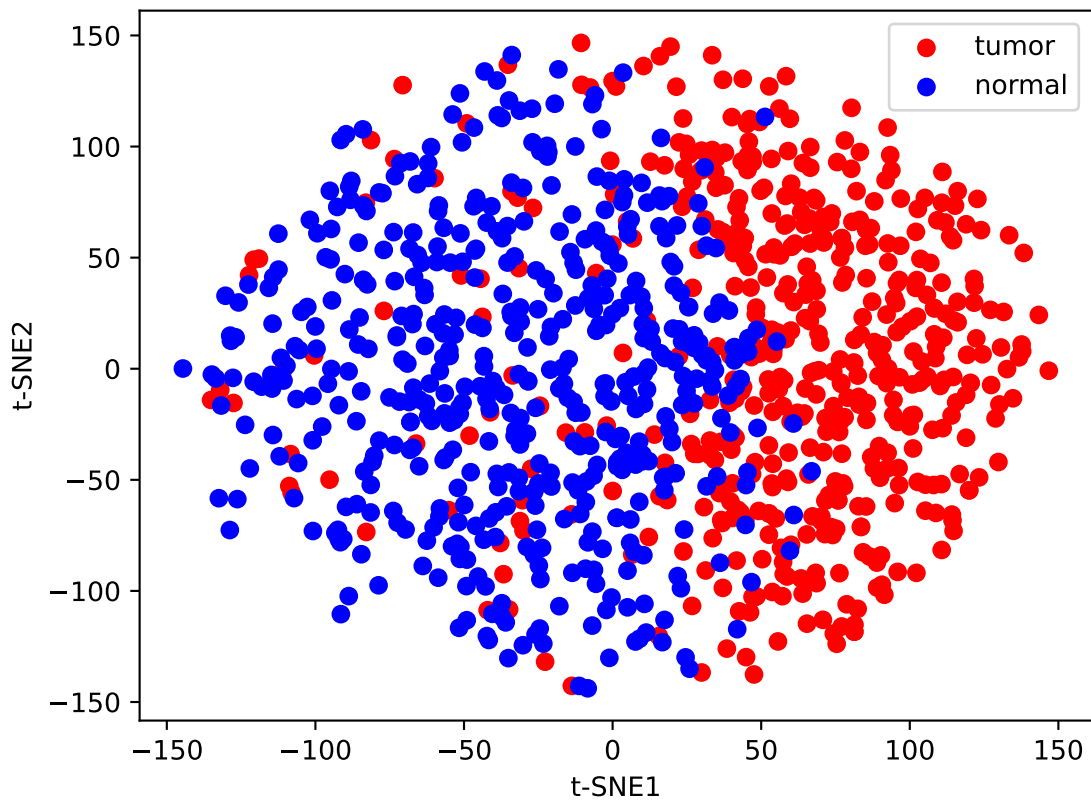

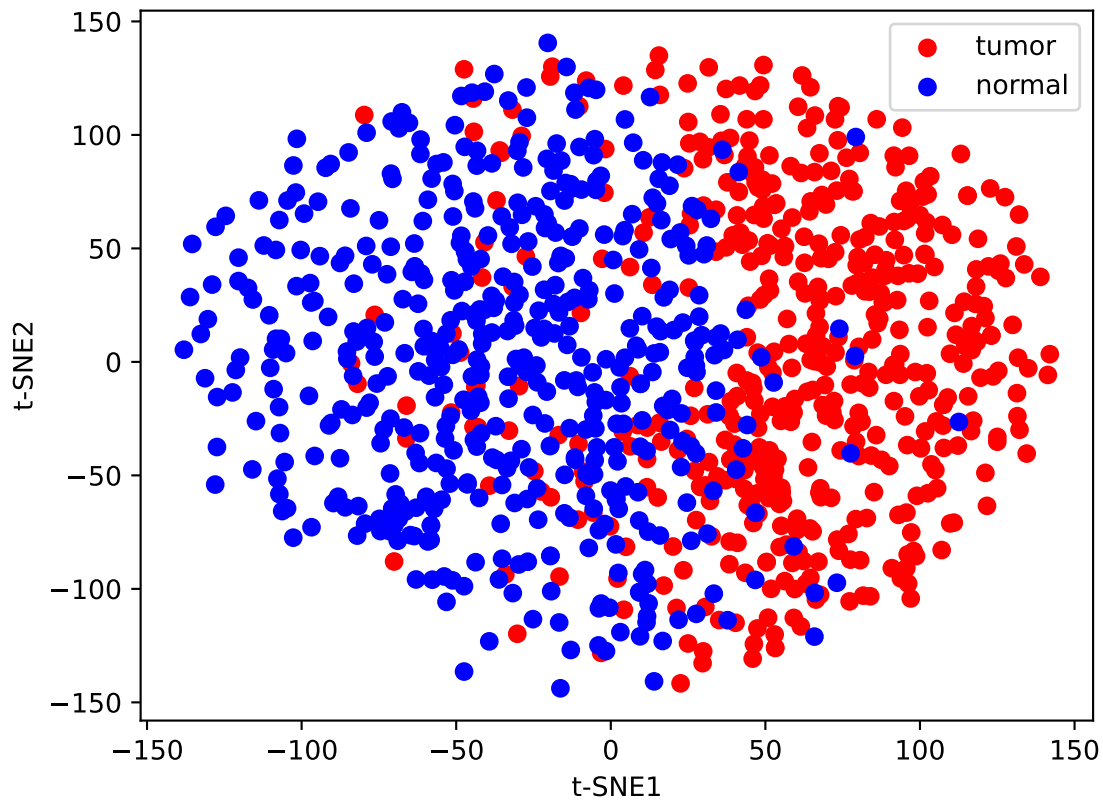

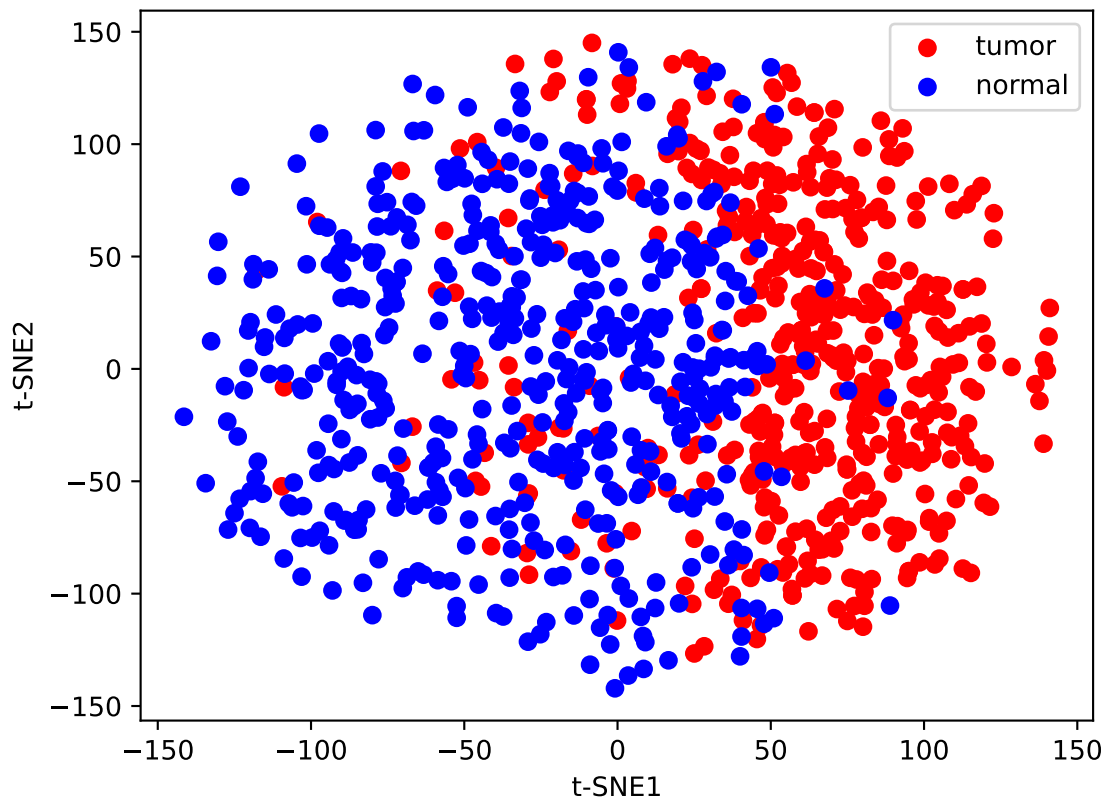

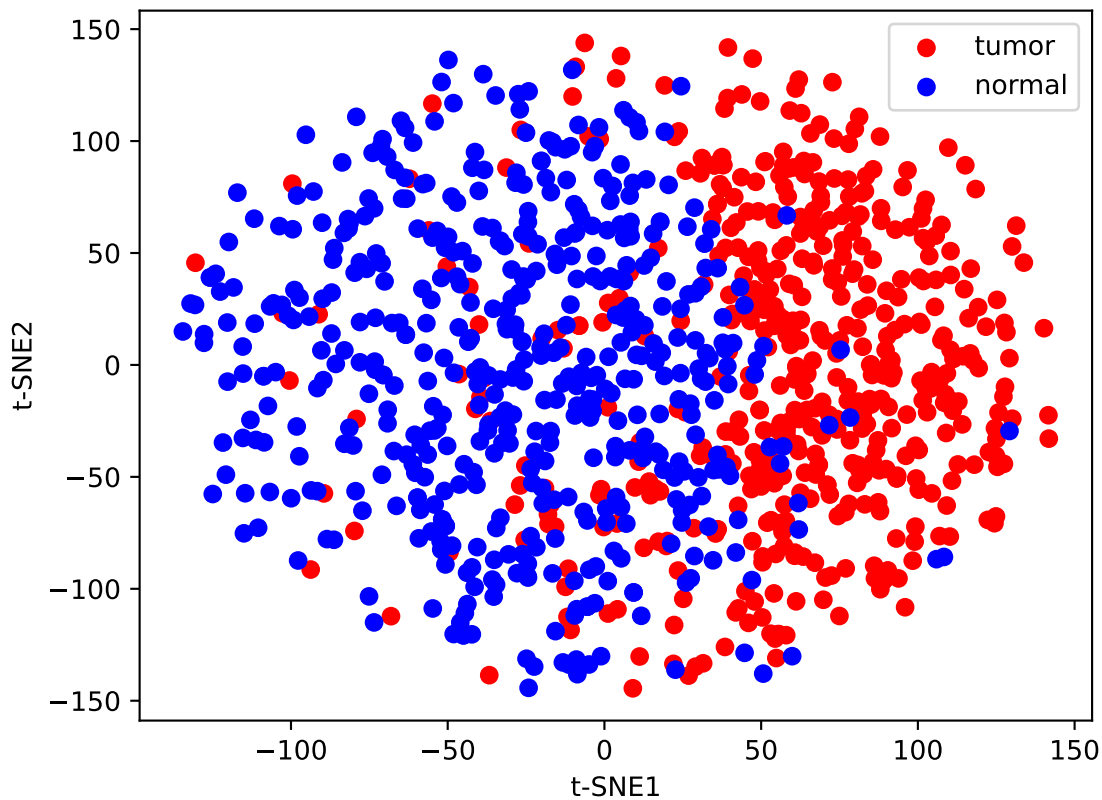

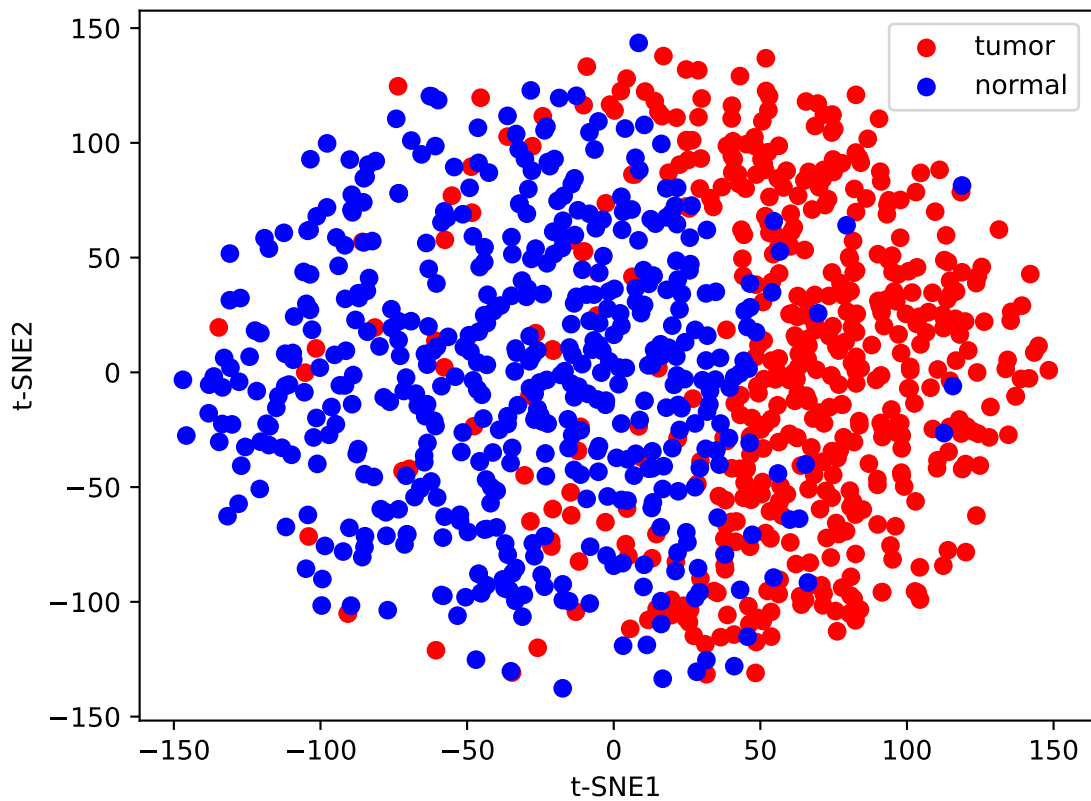

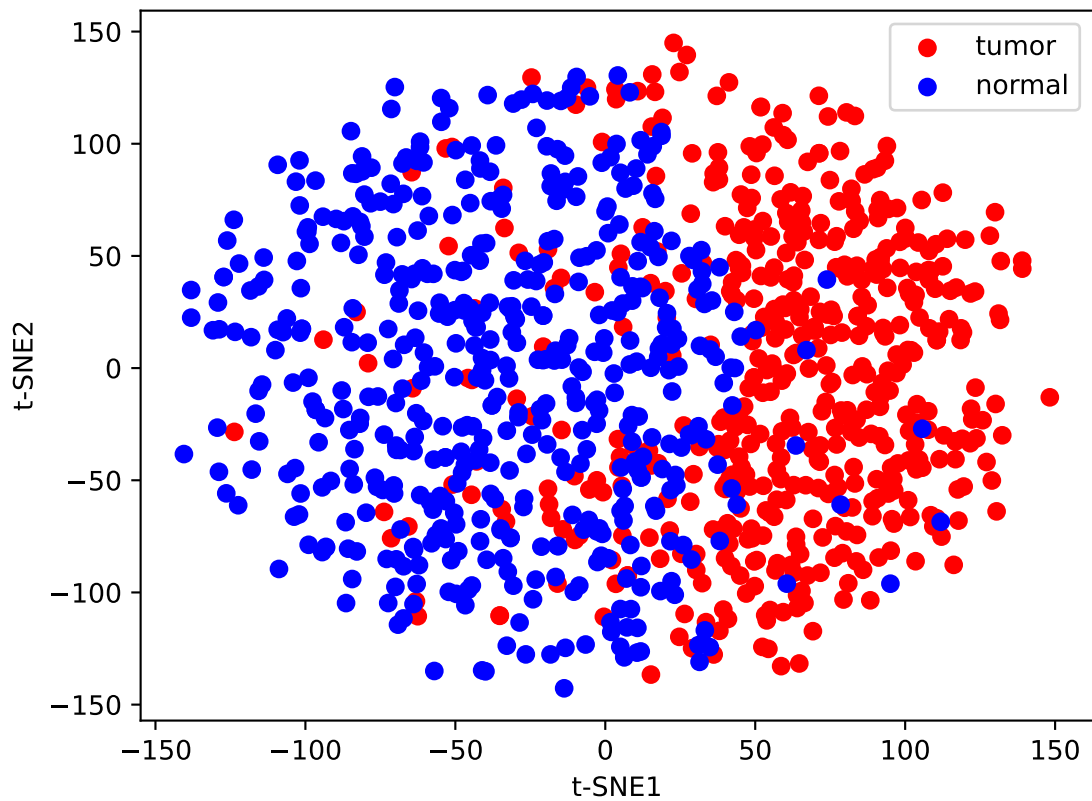

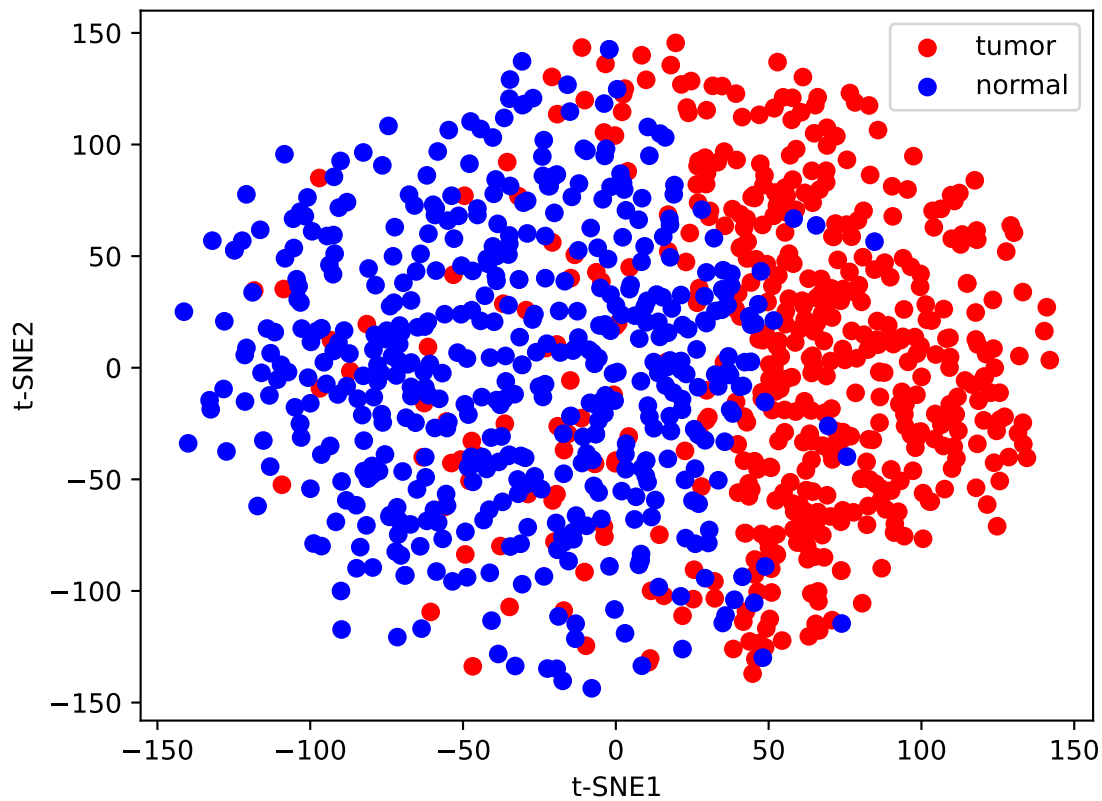

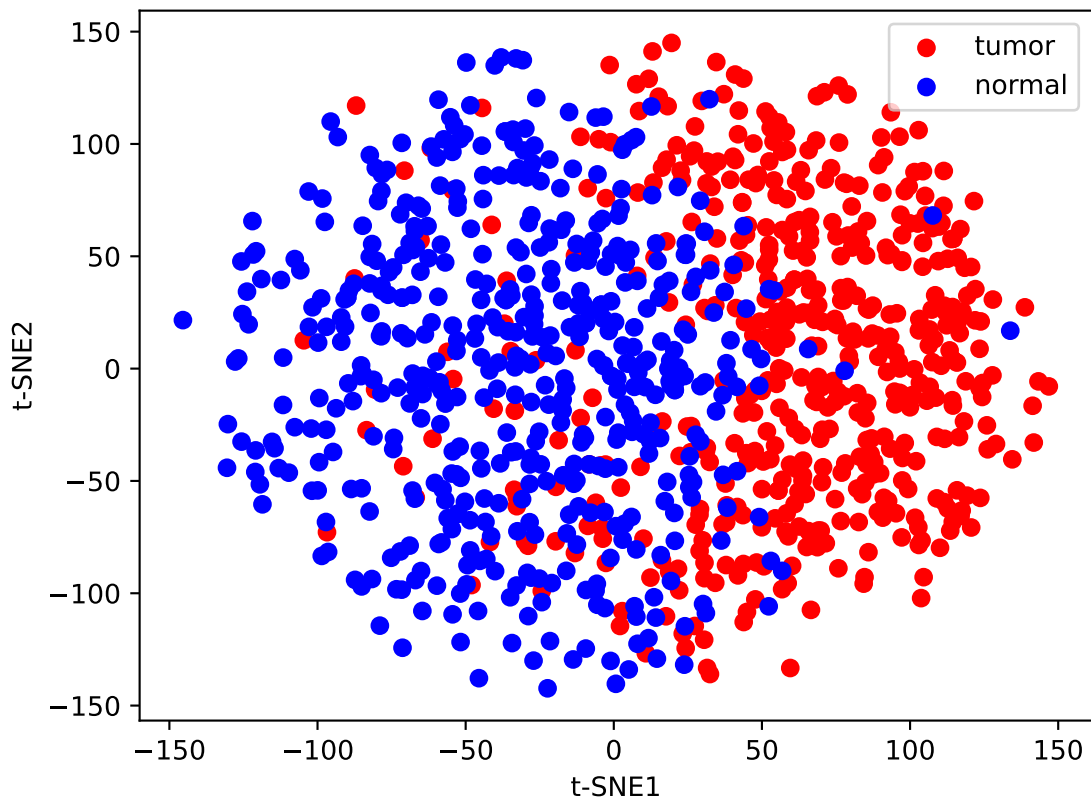

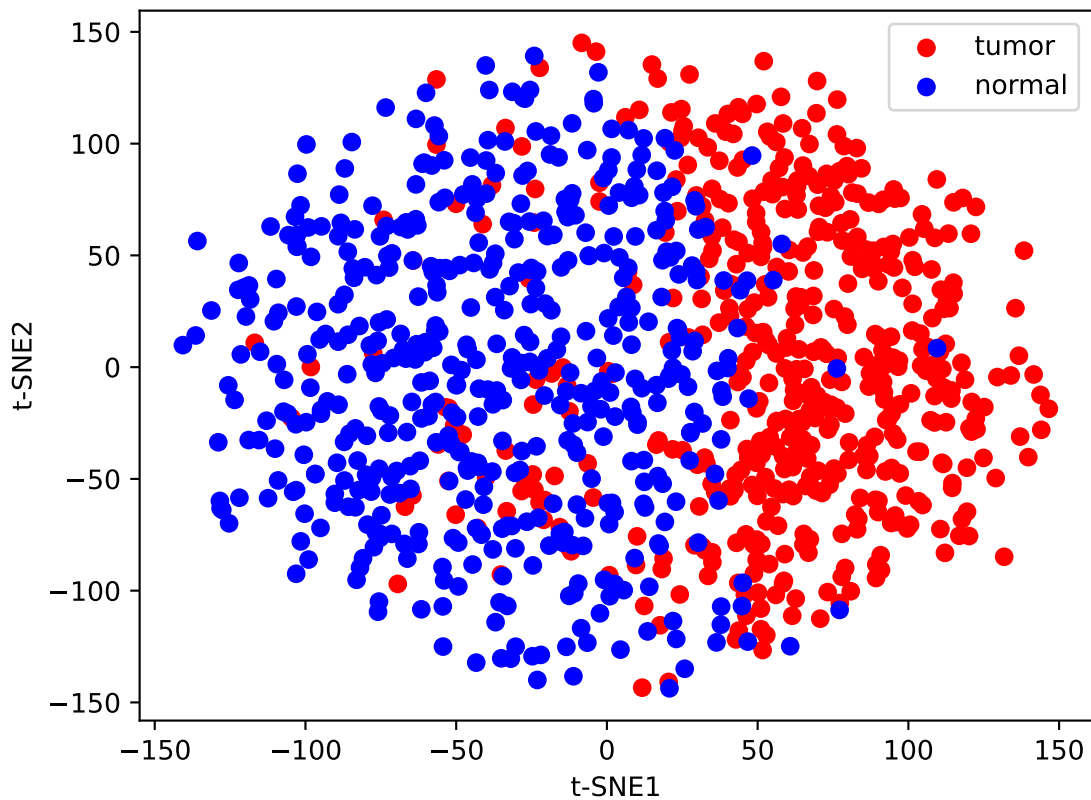

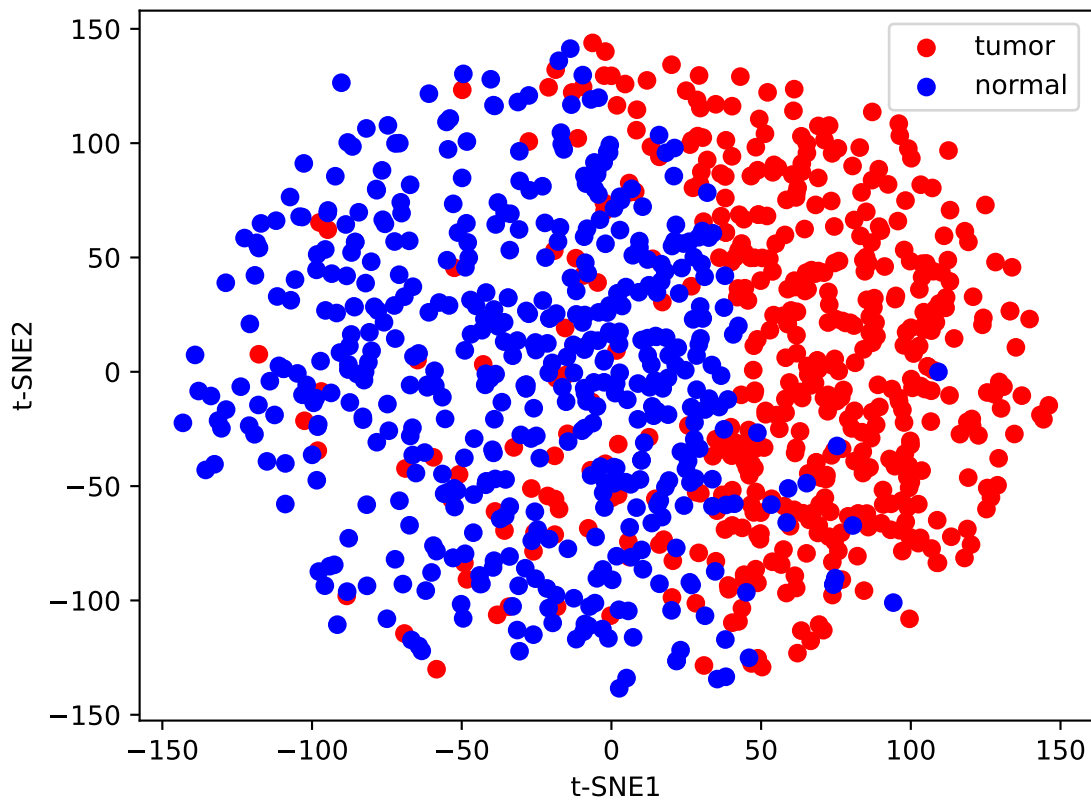

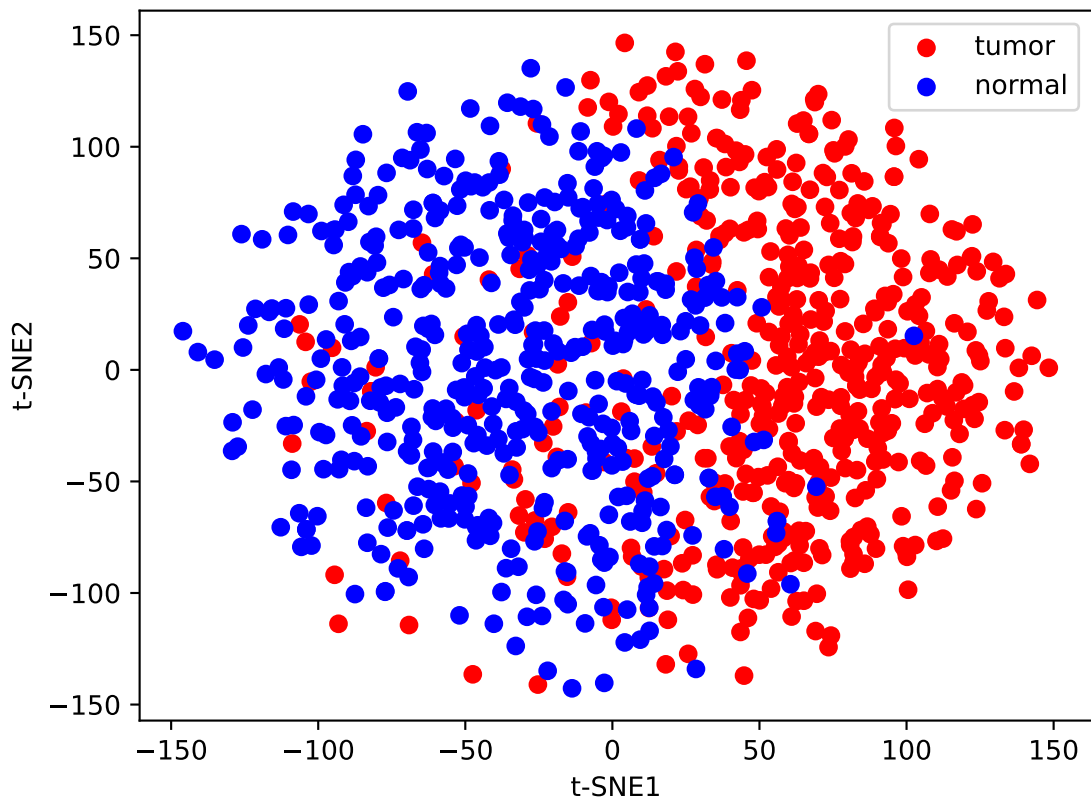

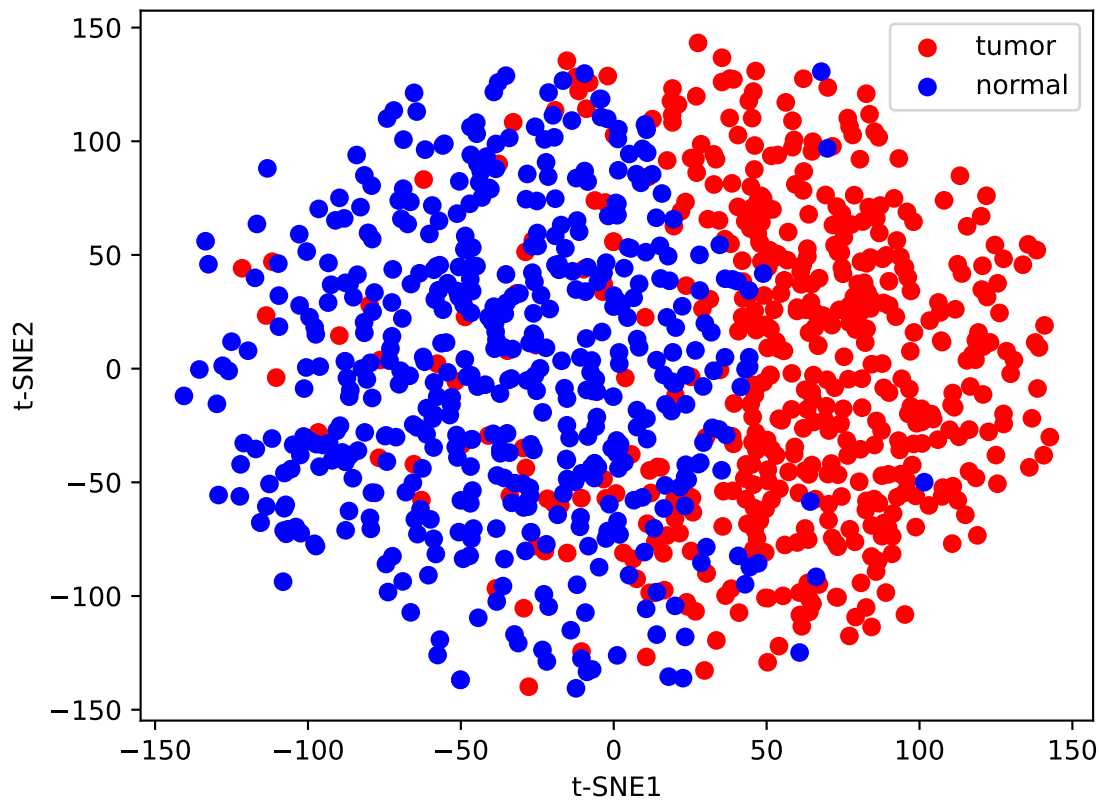

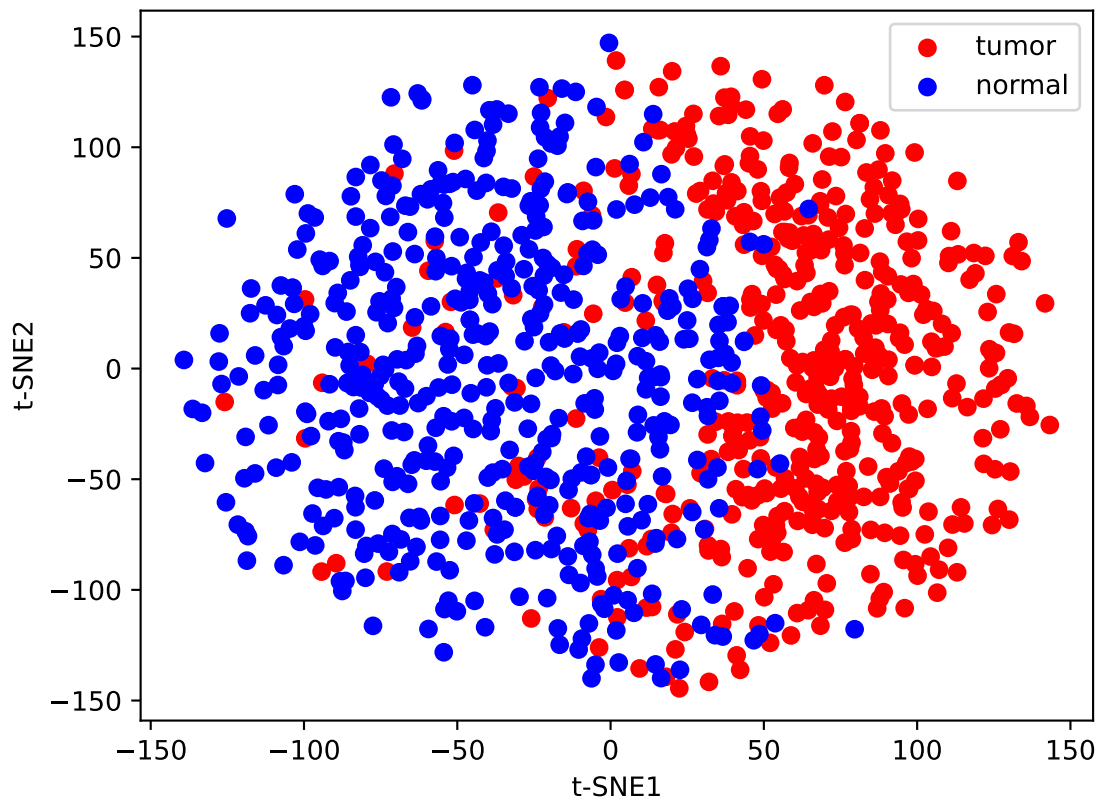

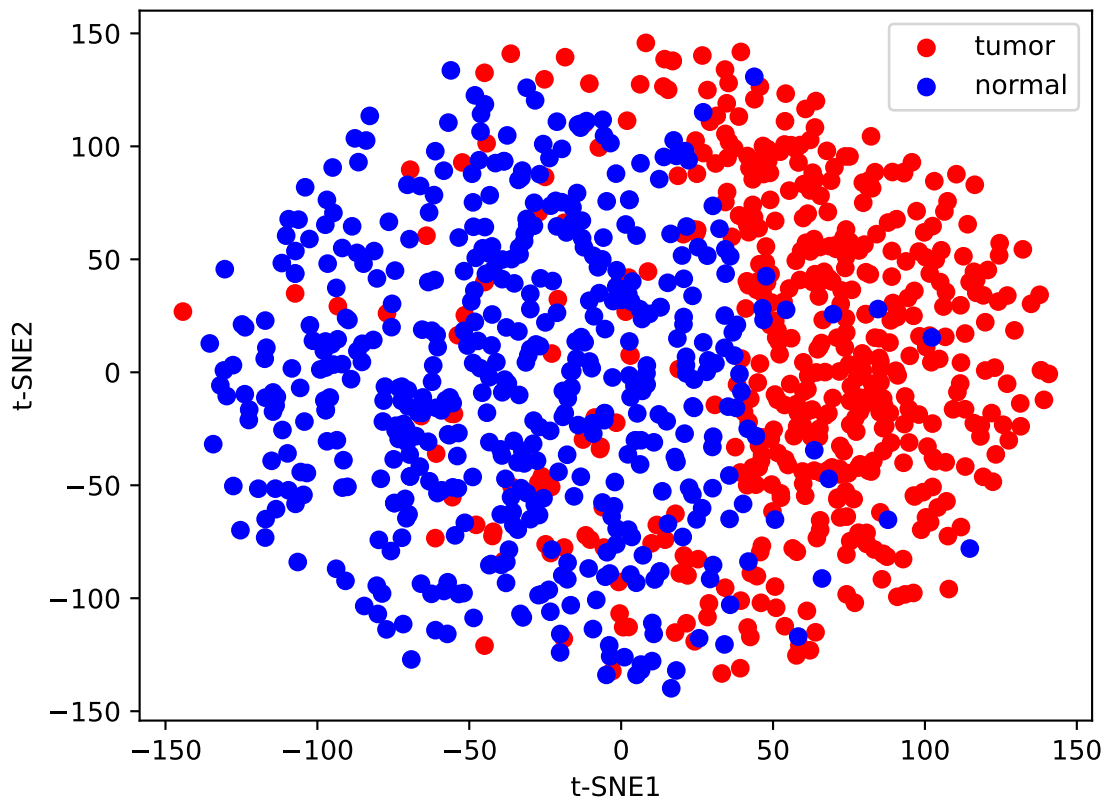

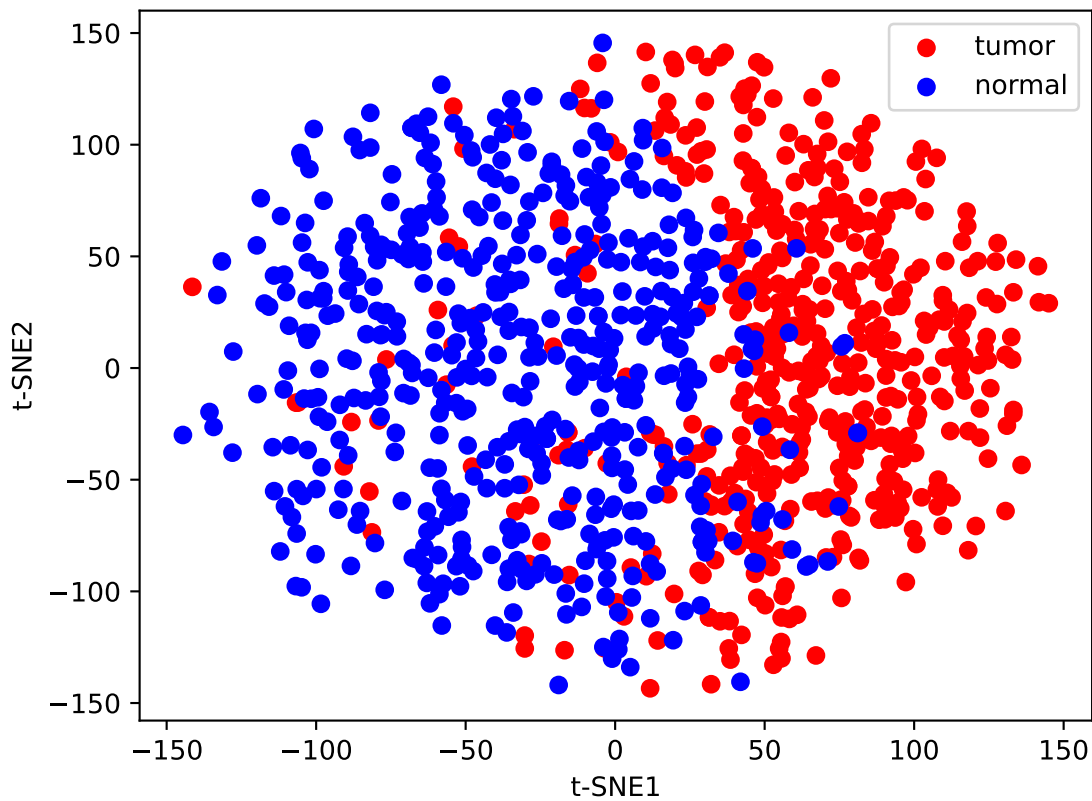

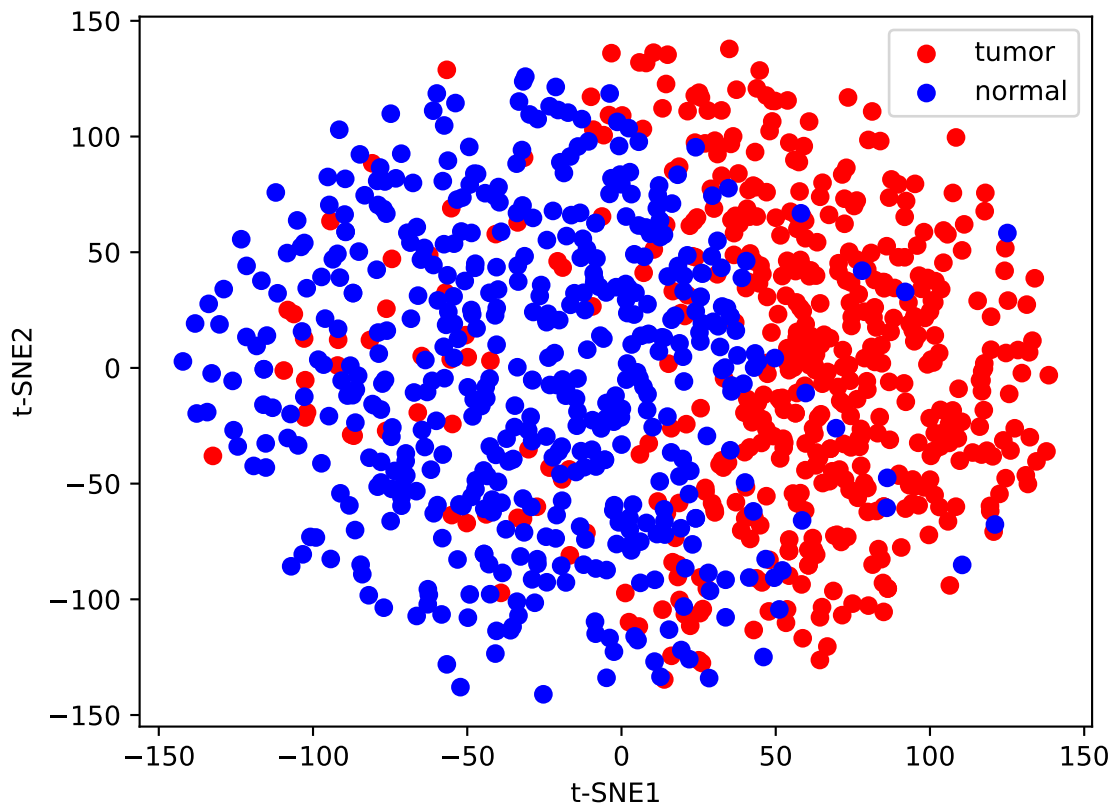

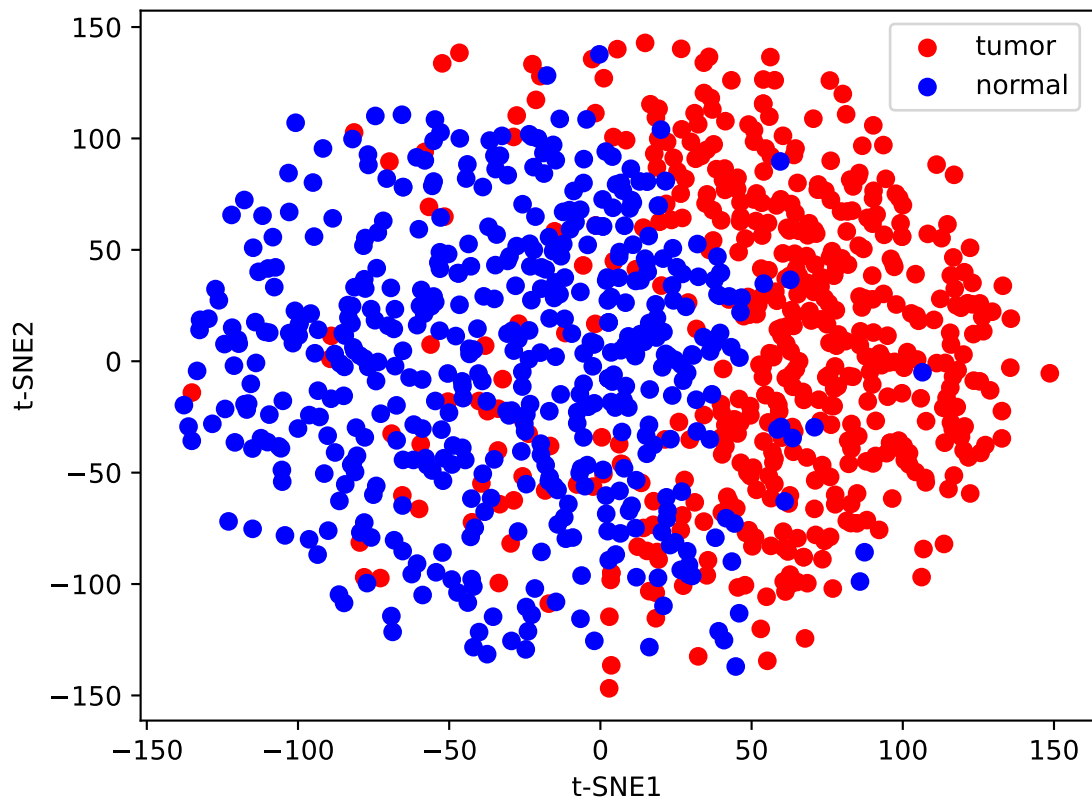

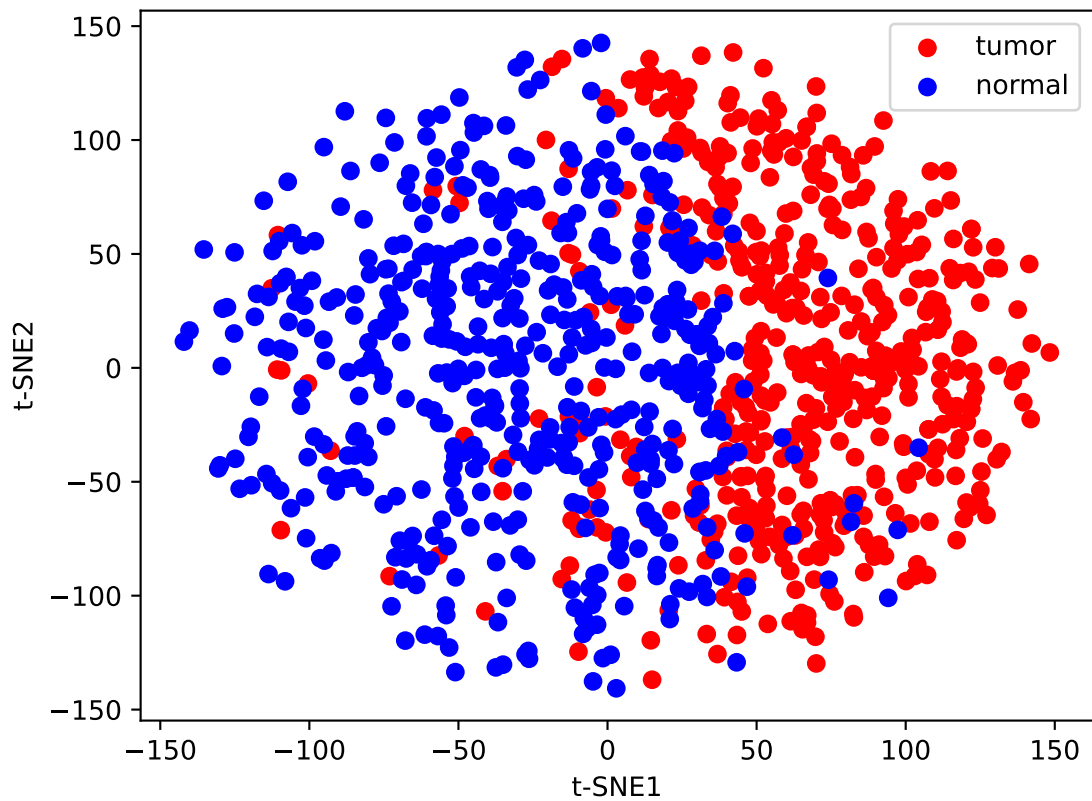

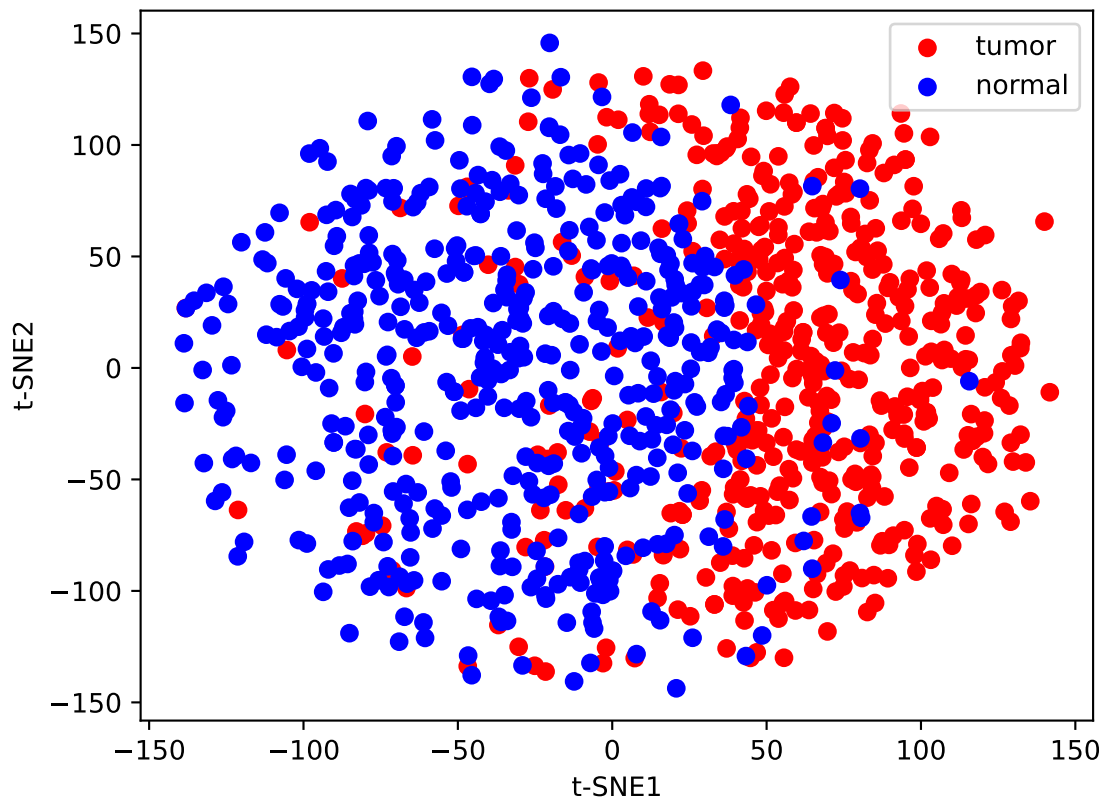

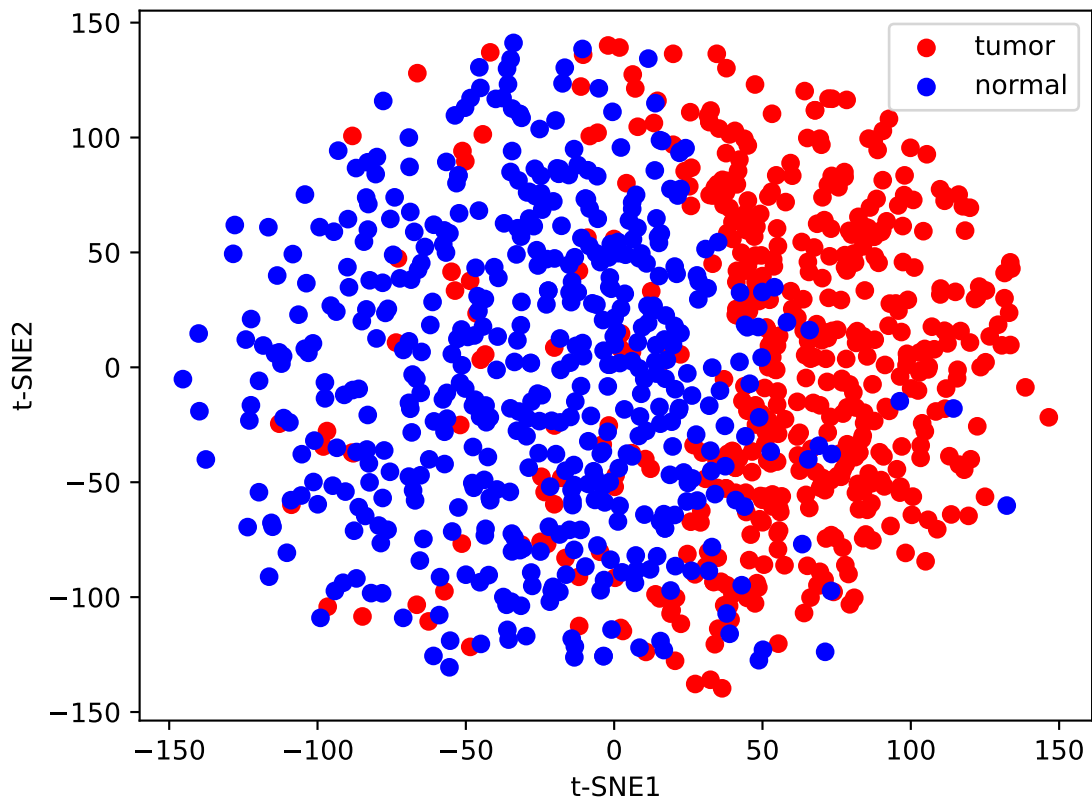

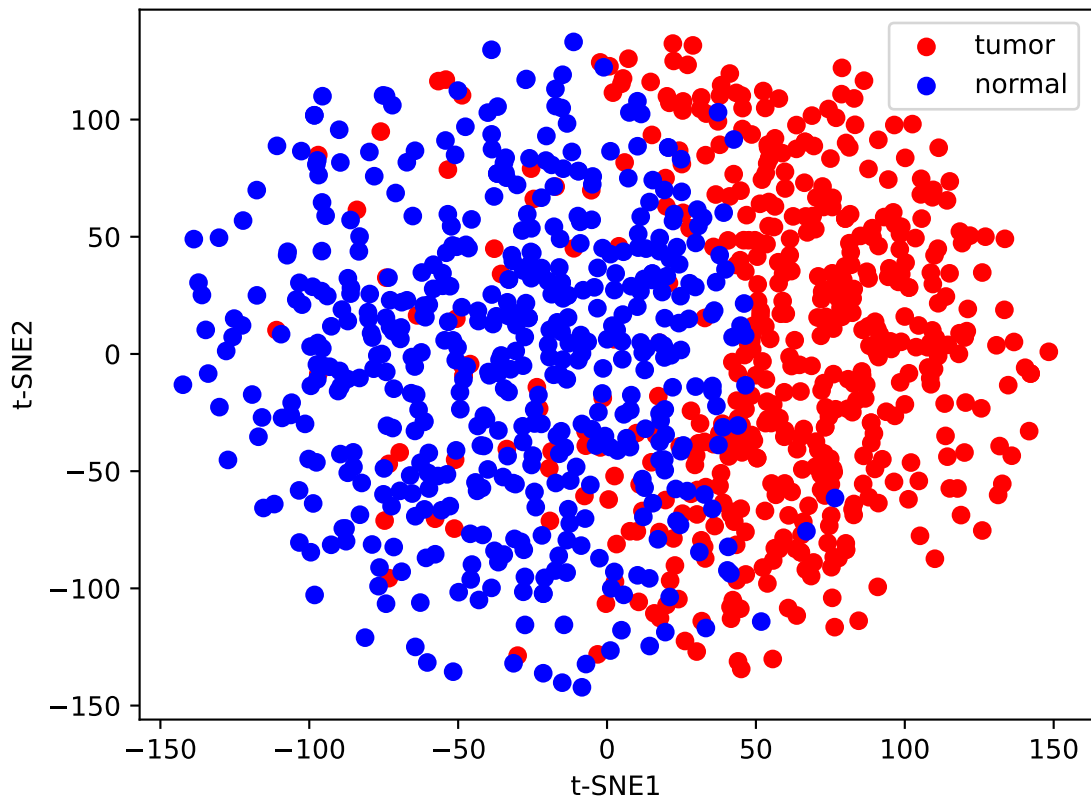

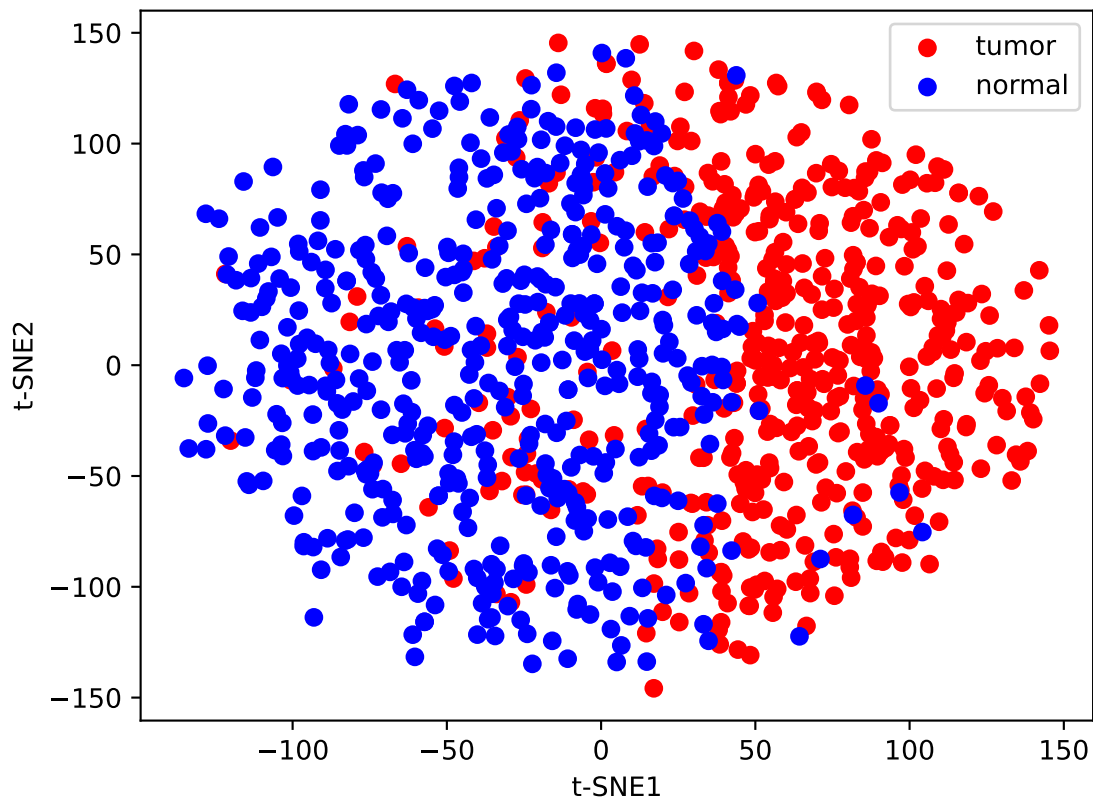

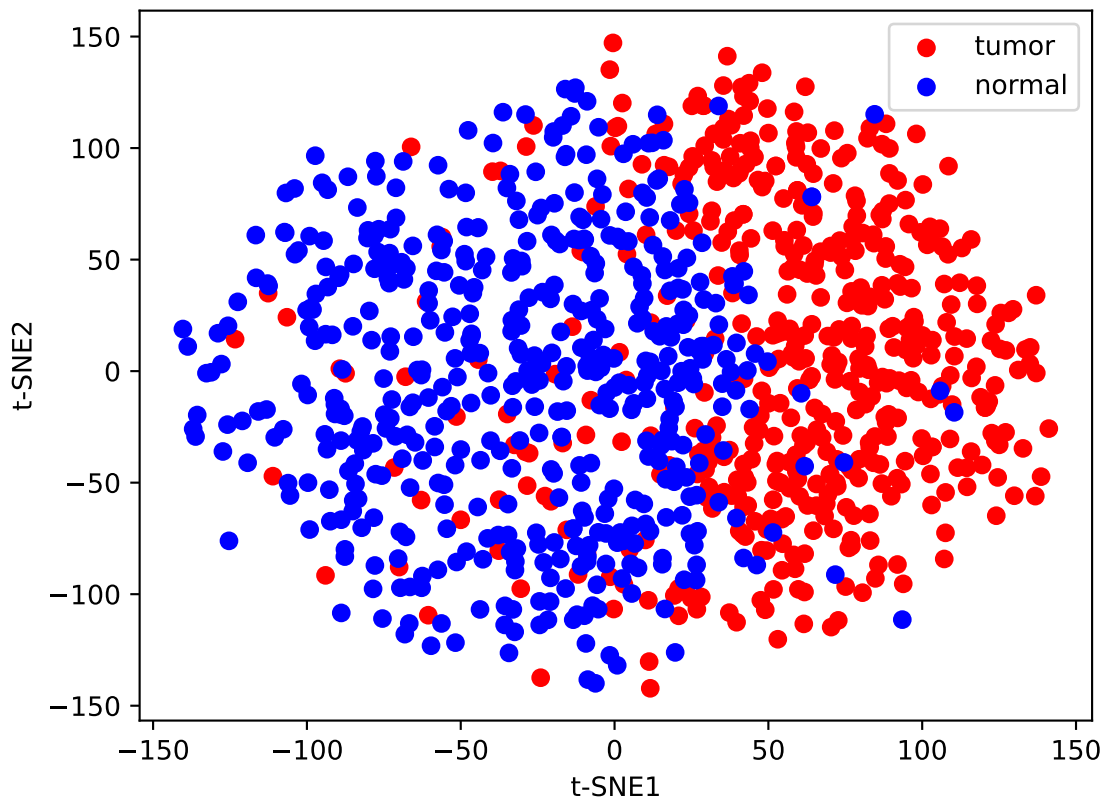

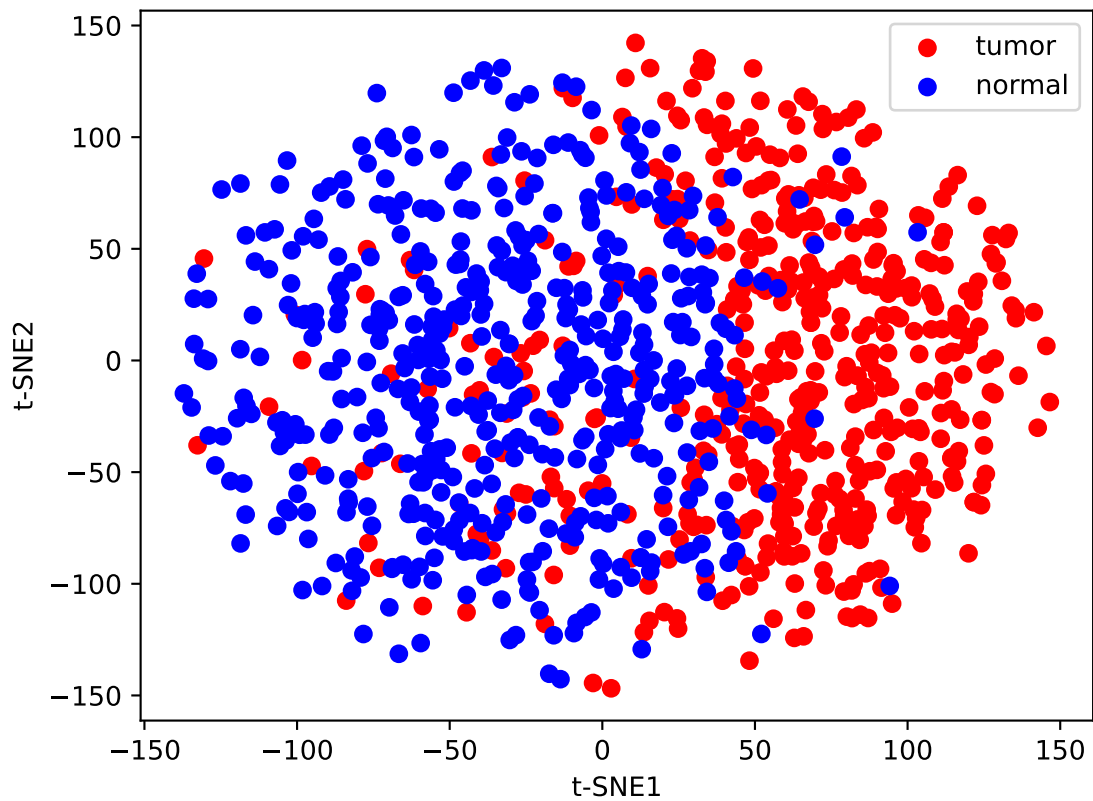

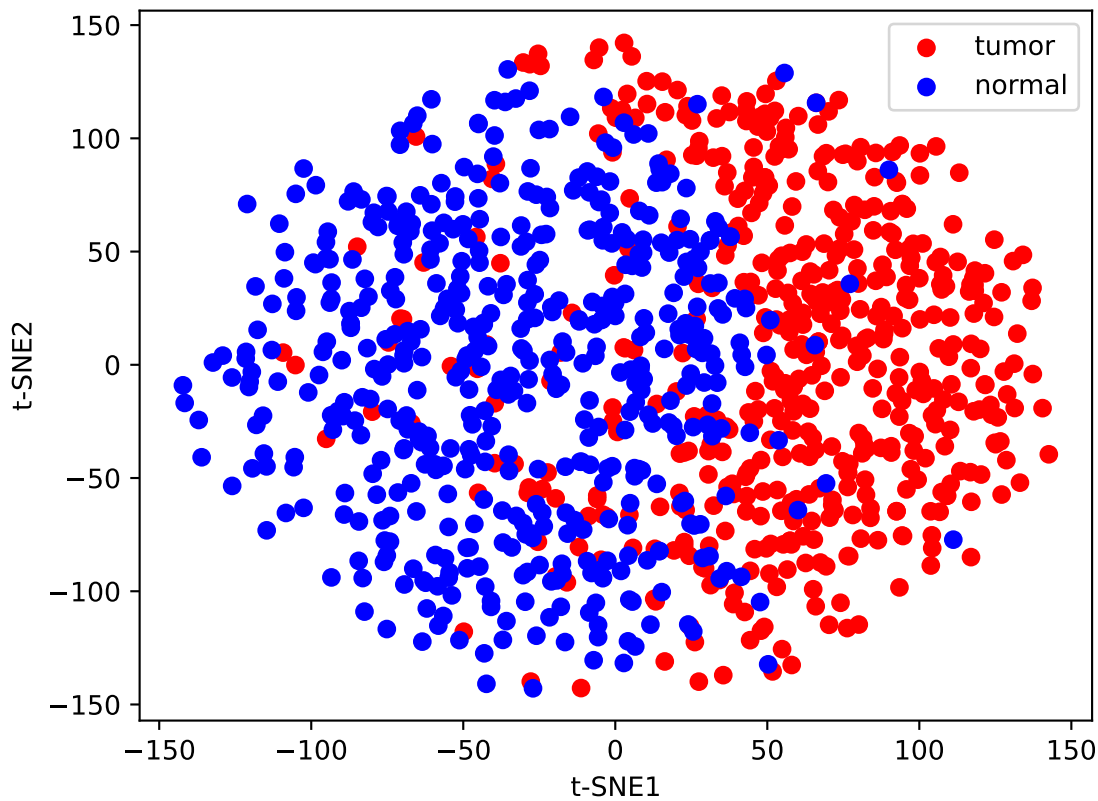

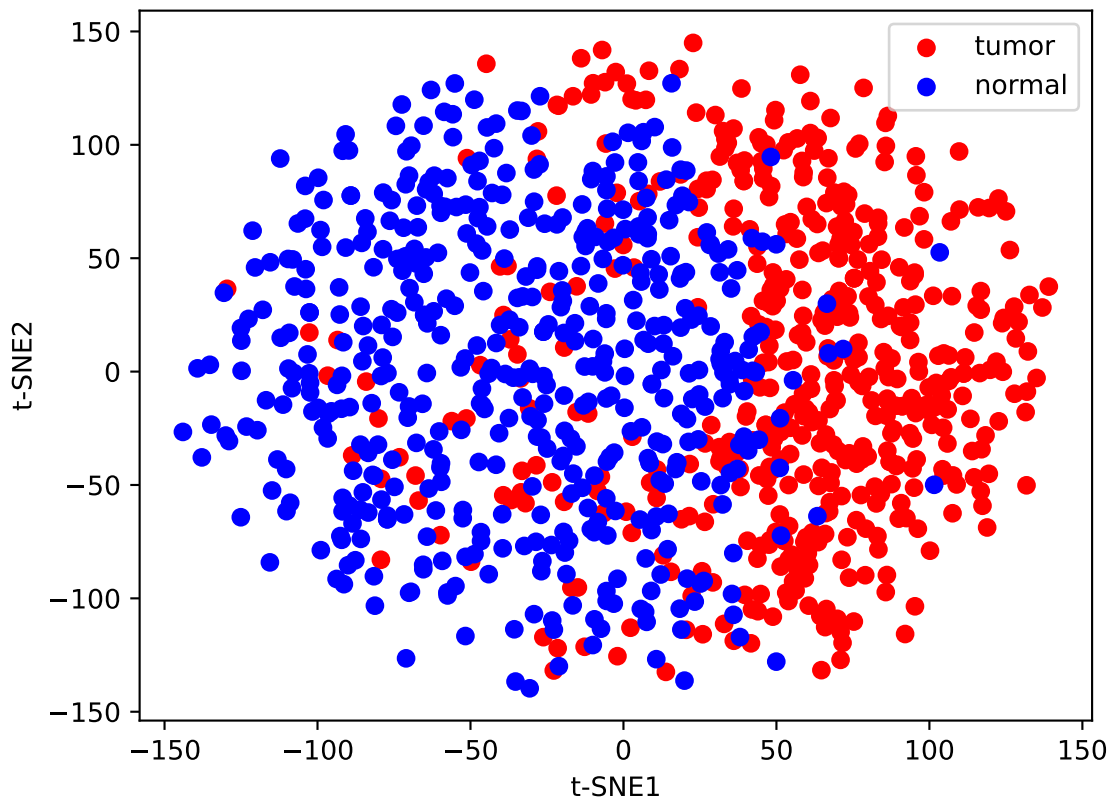

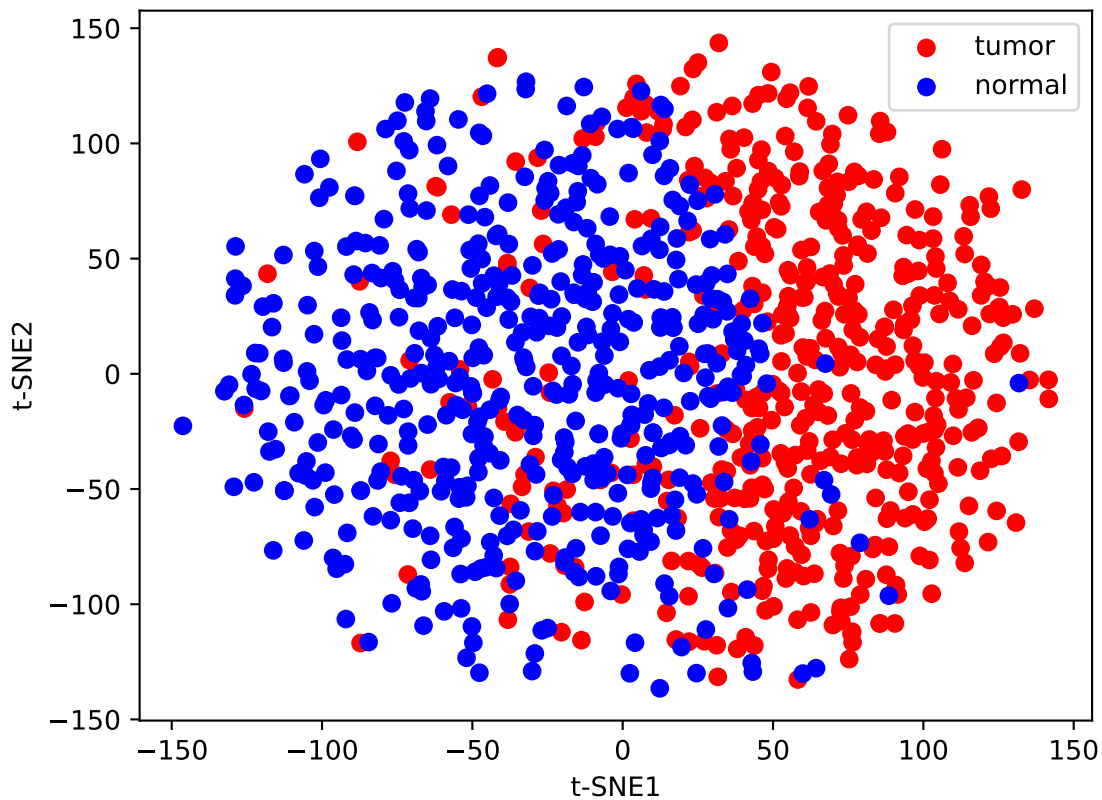

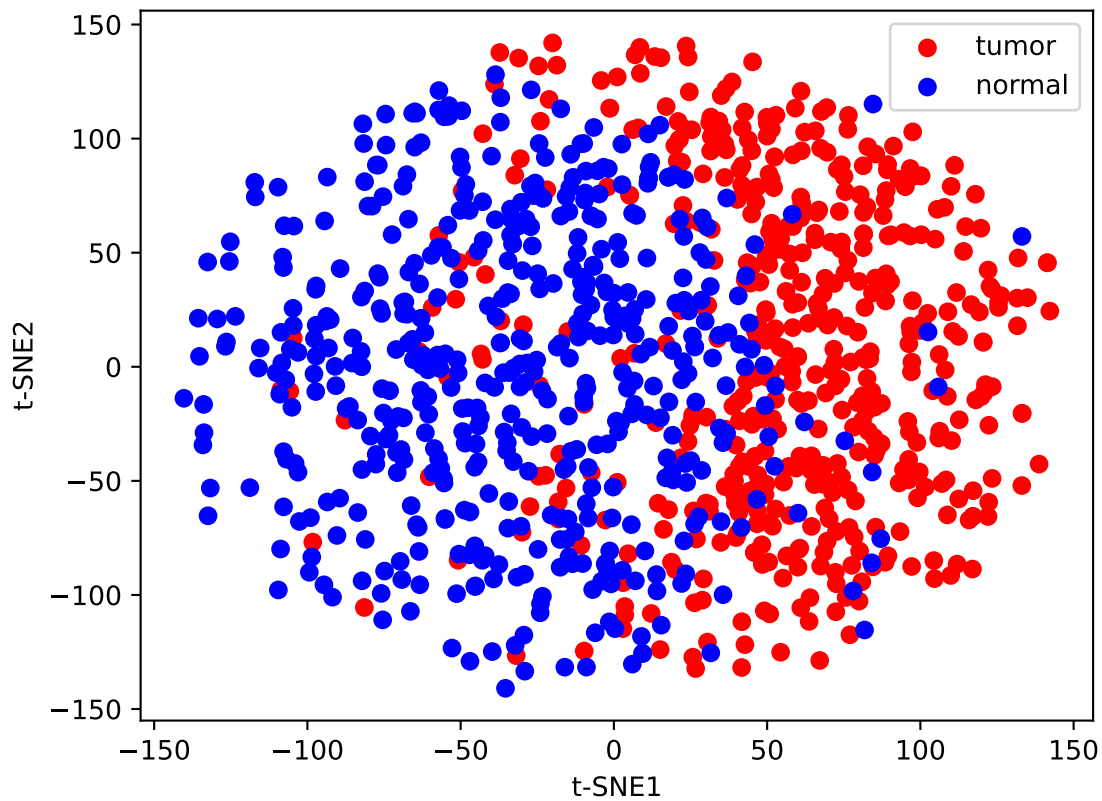

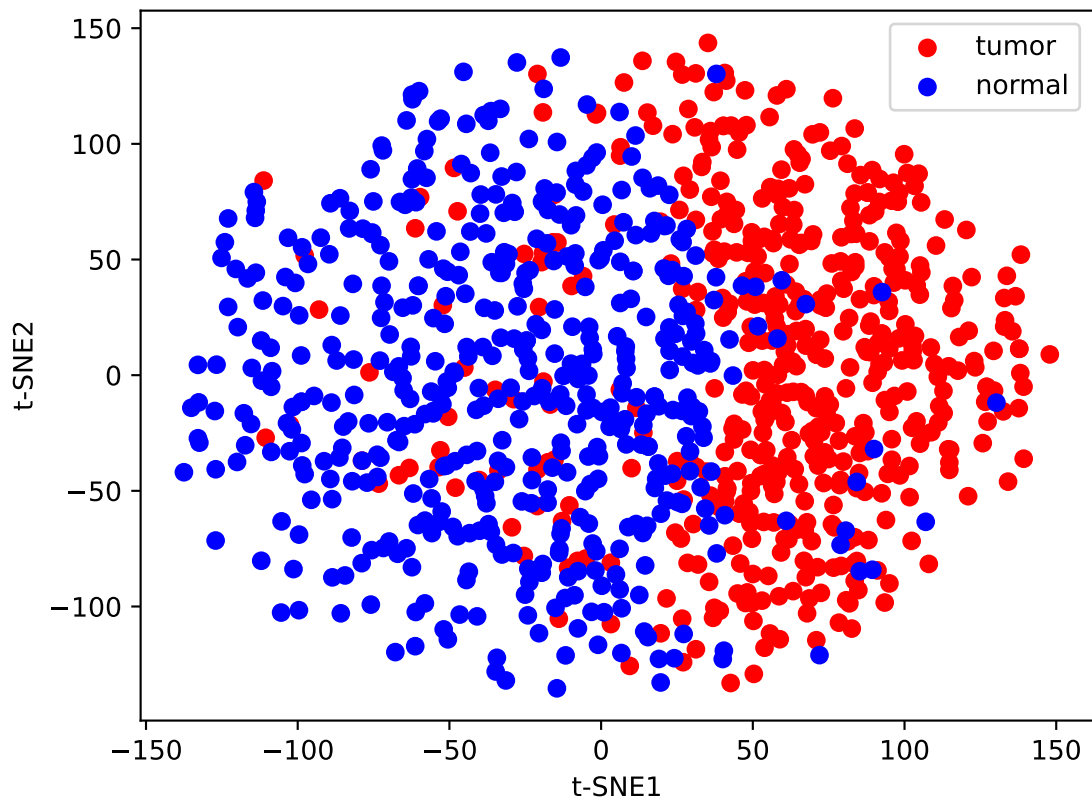

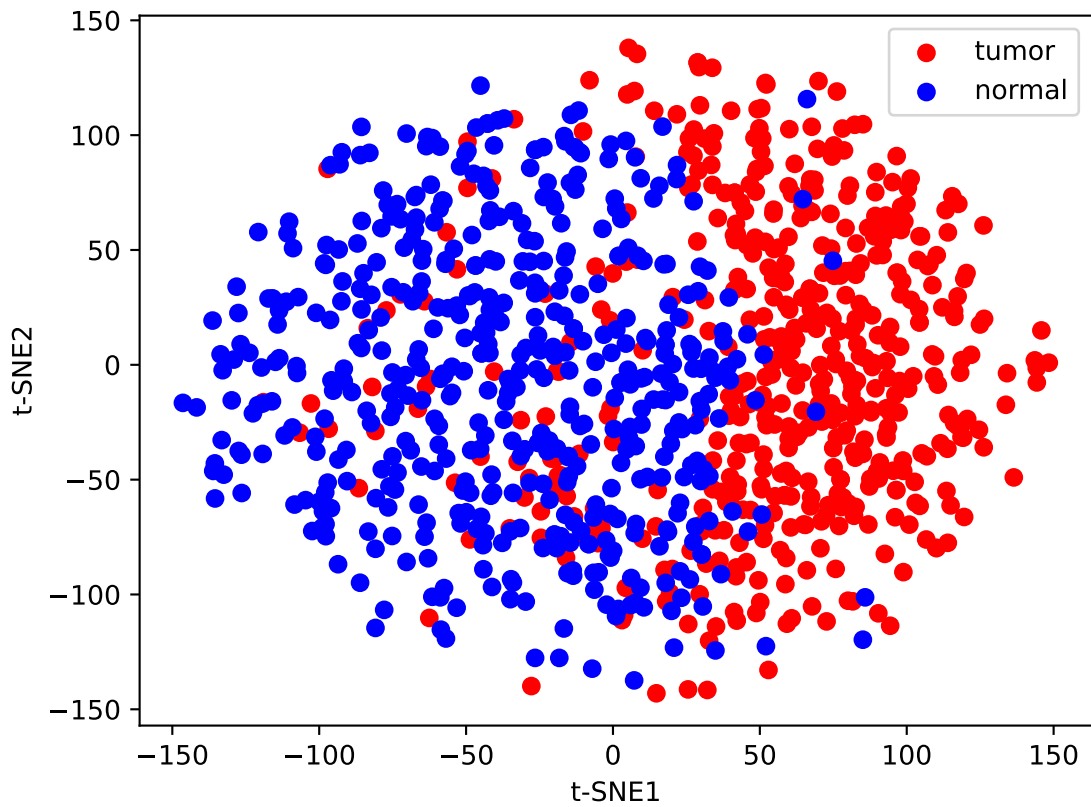

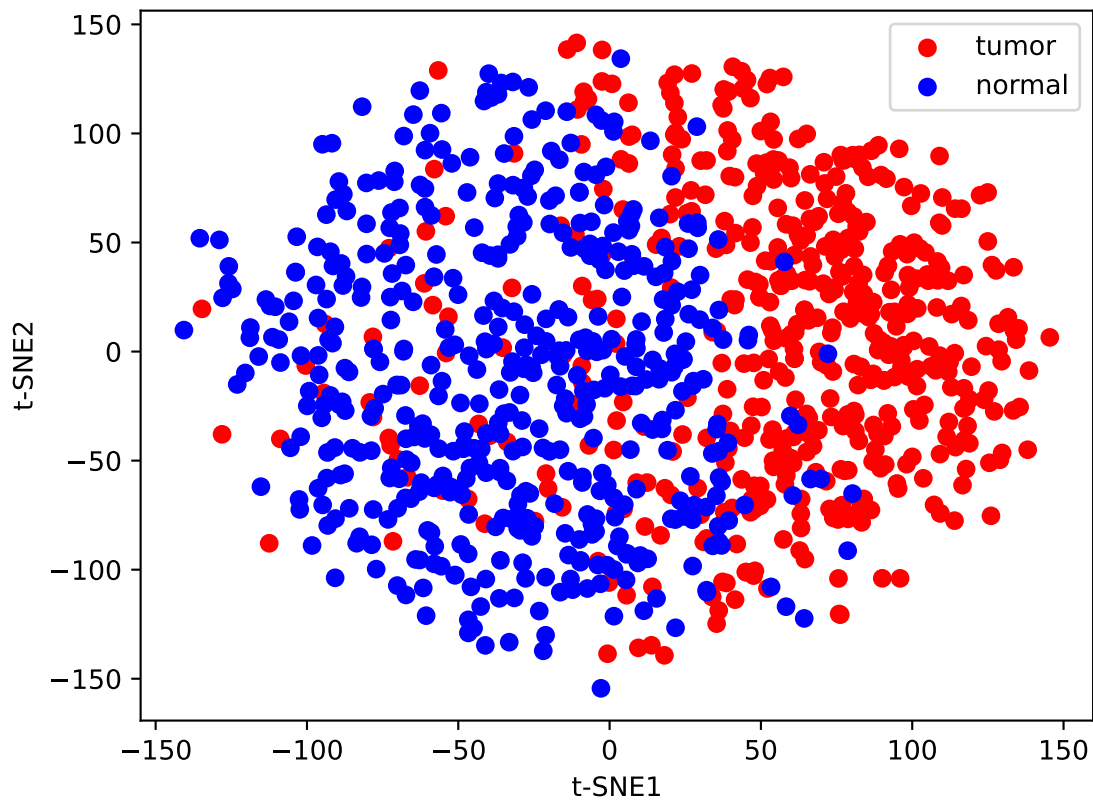

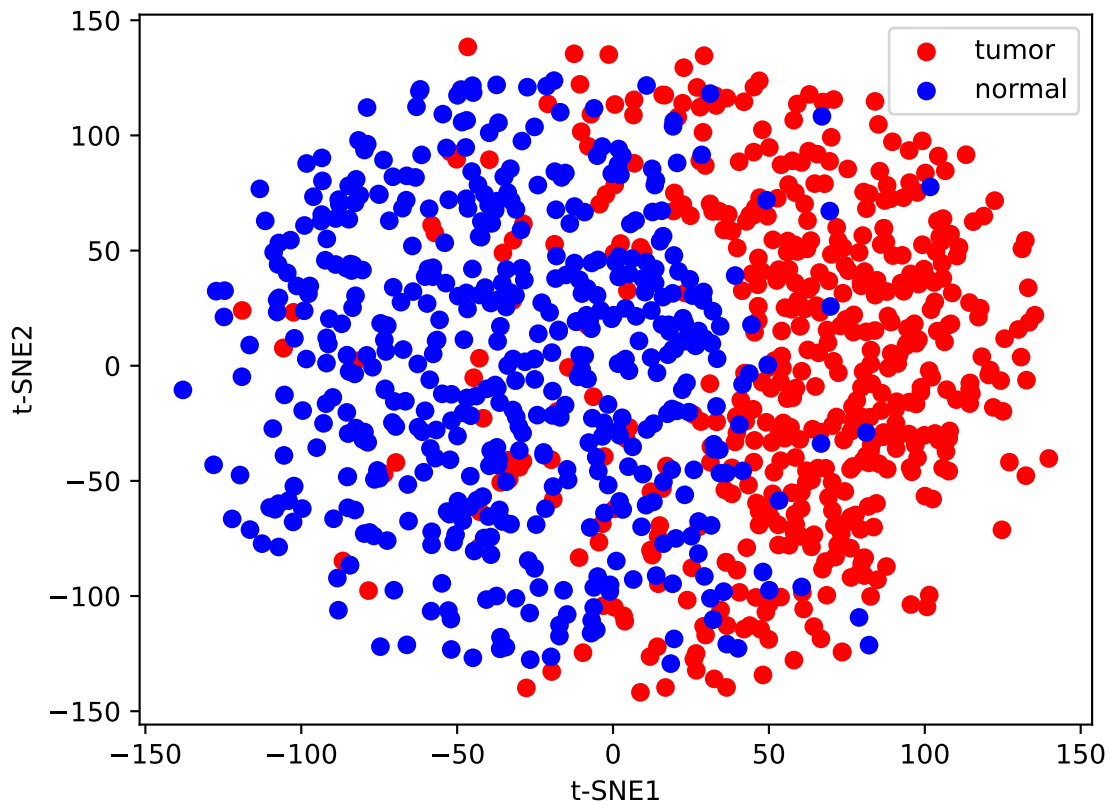

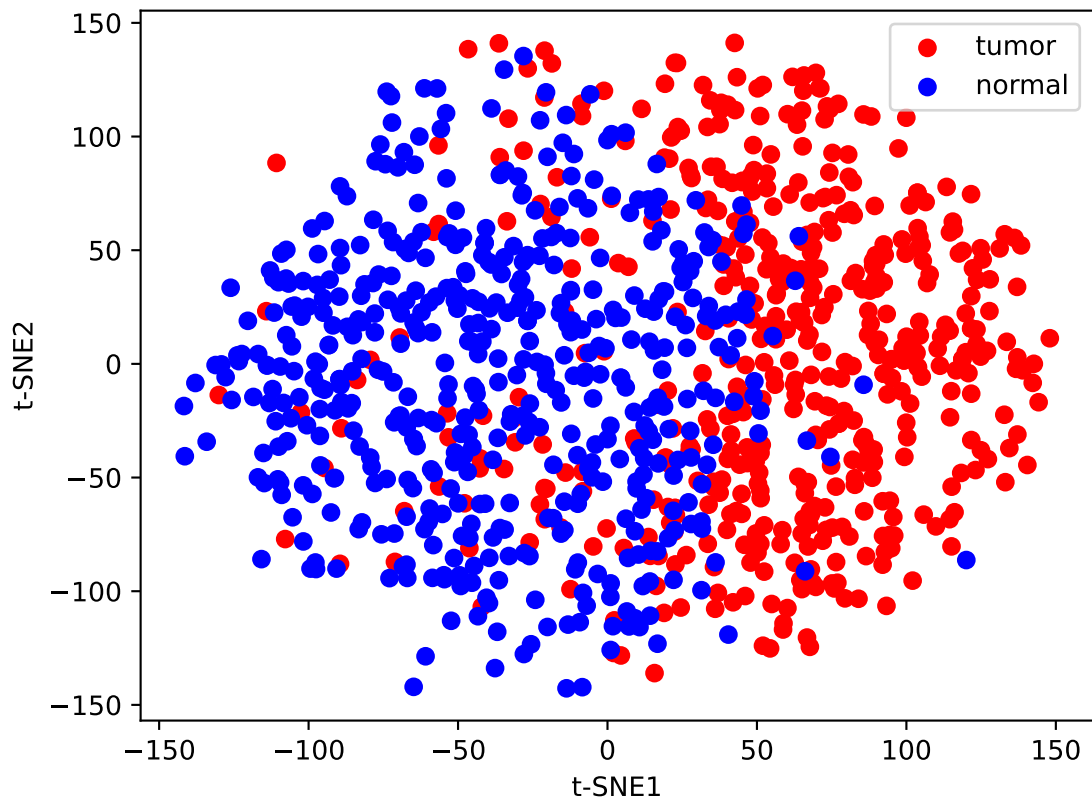

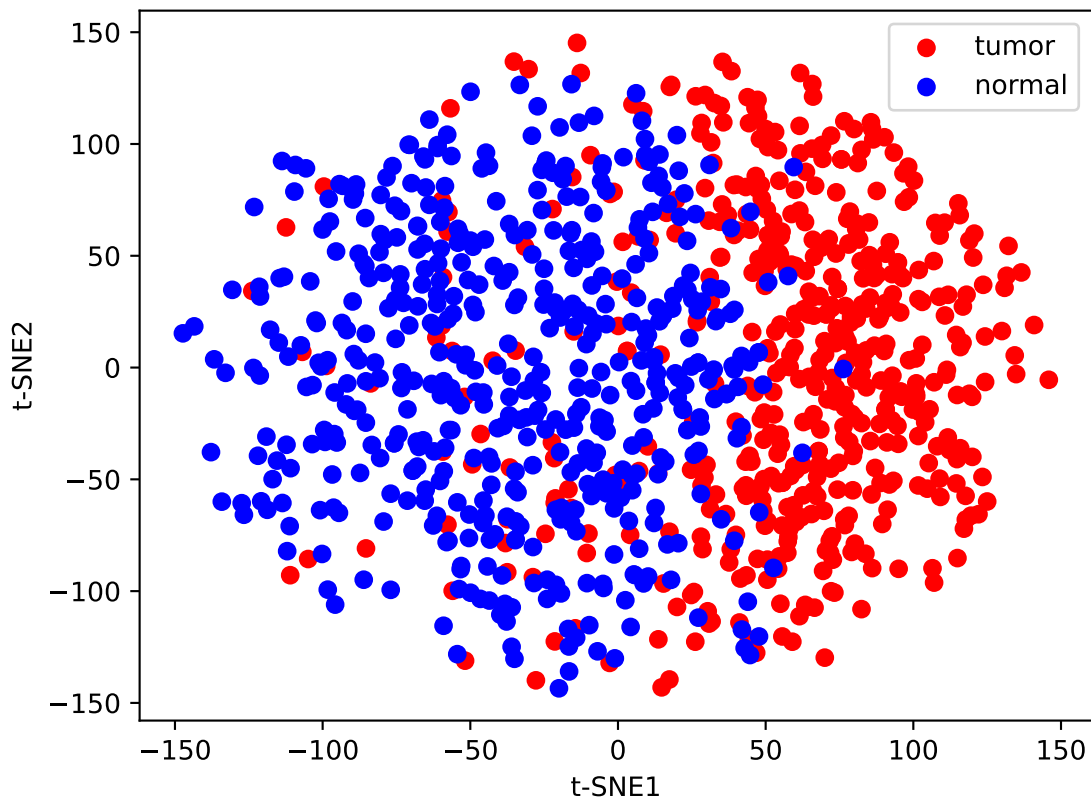

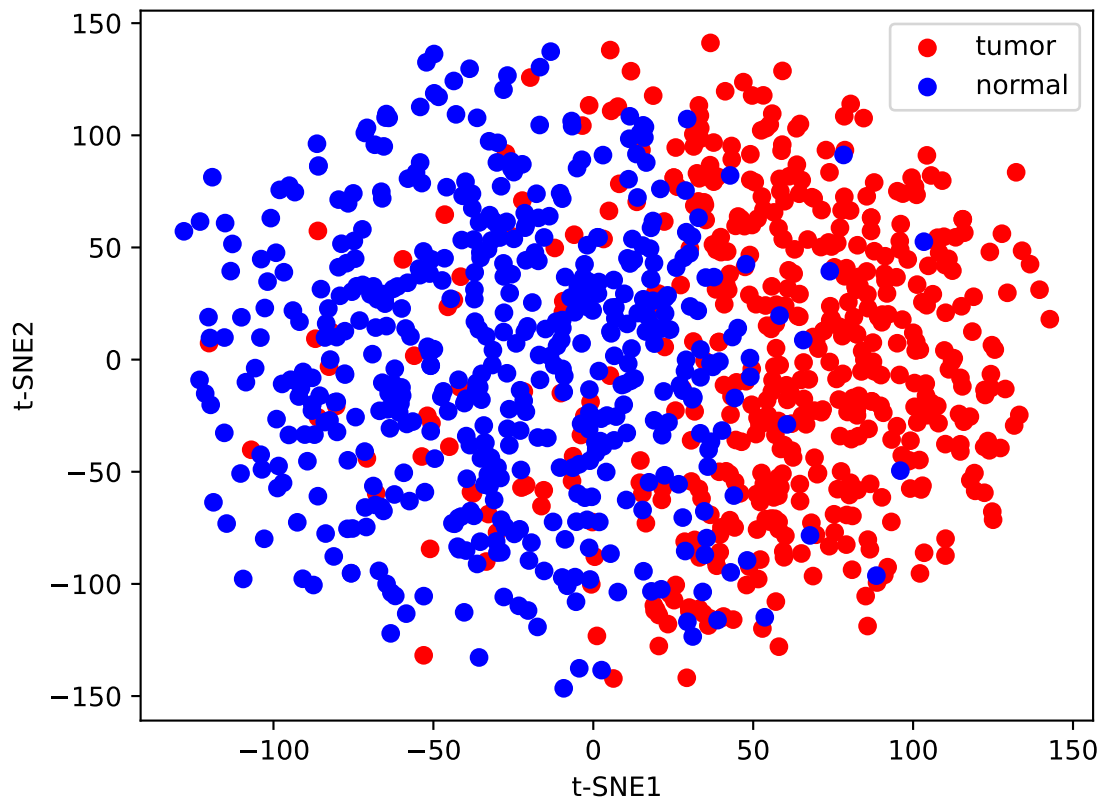

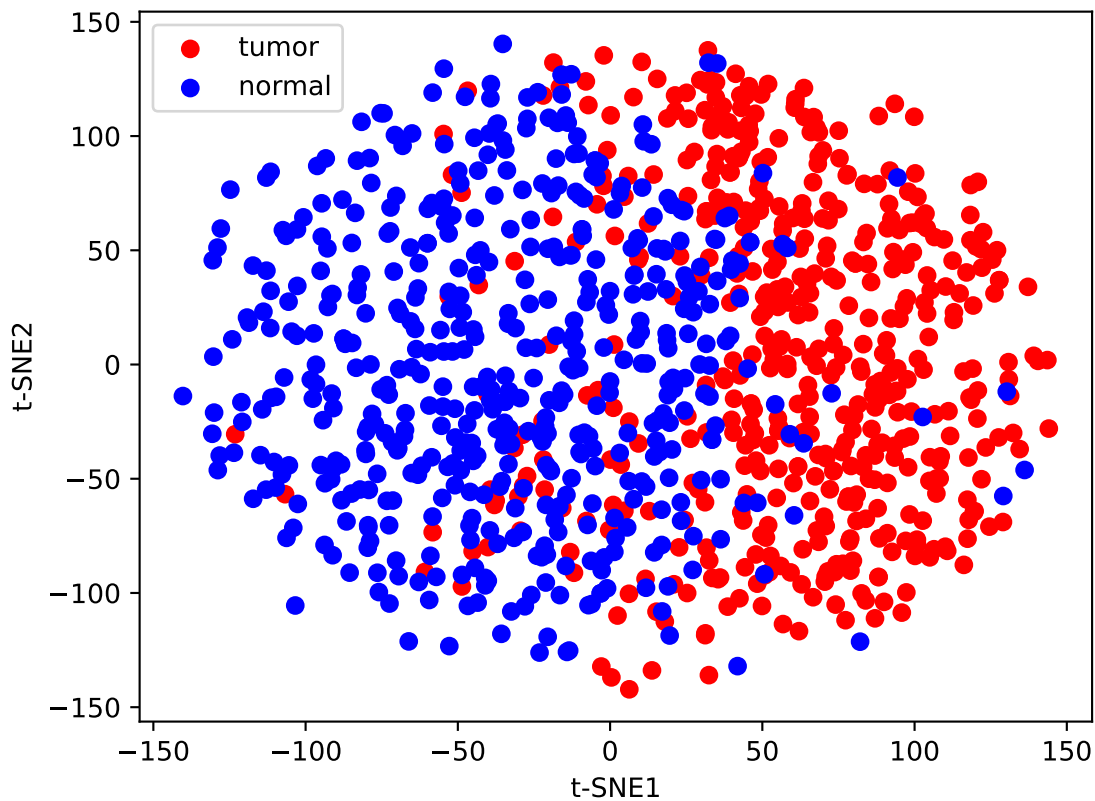

Supplement: Supplementary file 1 [file ijms-25-09827-s001.zip › Supplementary Materials S2.pdf]
